# Characterization of the Canine MHC Class I DLA-88*50101 Peptide Binding Motif as a Prerequisite for Canine T Cell Immunotherapy

**DOI:** 10.1371/journal.pone.0167017

**Published:** 2016-11-28

**Authors:** Sharon M. Barth, Christian M. Schreitmüller, Franziska Proehl, Kathrin Oehl, Leonie M. Lumpp, Daniel J. Kowalewski, Moreno Di Marco, Theo Sturm, Linus Backert, Heiko Schuster, Stefan Stevanović, Hans-Georg Rammensee, Oliver Planz

**Affiliations:** 1 Department of Immunology, Institute of Cell Biology, University of Tuebingen, Tuebingen, Germany; 2 Institute for Surgical Pathology, University Hospital Zurich, Zurich, Switzerland; 3 Immatics, Biotechnologies GmbH, Tuebingen, Germany; 4 Institute of Molecular Systems Biology, Department of Biology, ETH Zurich, Zurich, Switzerland; 5 Biomolecular Mass Spectrometry and Proteomics, Bijvoet Center for Biomolecular Research and Utrecht Institute for Pharmaceutical Sciences, Utrecht University, Utrecht, The Netherlands; 6 Applied Bioinformatics, Center of Bioinformatics and Department of Computer Science, University of Tuebingen, Tuebingen, Germany; Centro Nacional de Biotecnologia, SPAIN

## Abstract

There are limitations in pre-clinical settings using mice as a basis for clinical development in humans. In cancer, similarities exist between humans and dogs; thus, the dog patient can be a link in the transition from laboratory research on mouse models to clinical trials in humans. Knowledge of the peptides presented on MHC molecules is fundamental for the development of highly specific T cell-based immunotherapies. This information is available for human MHC molecules but is absent for the canine MHC. In the present study, we characterized the binding motif of dog leukocyte antigen (DLA) class I allele DLA-88*50101, using human C1R and K562 transfected cells expressing the DLA-88*50101 heavy chain. MHC class I immunoaffinity-purification revealed 3720 DLA-88*50101 derived peptides, which enabled the determination of major anchor positions. The characterized binding motif of DLA-88*50101 was similar to HLA-A*02:01. Peptide binding analyses on HLA-A*02:01 and DLA-88*50101 via flow cytometry showed weak binding of DLA-88*50101 derived peptides to HLA-A*02:01, and vice versa. Our results present for the first time a detailed peptide binding motif of the canine MHC class I allelic product DLA-88*50101. These data support the goal of establishing dogs as a suitable animal model for the evaluation and development of T cell-based cancer immunotherapies, benefiting both dog and human patients.

## Introduction

New animal models better reflecting human biology could significantly improve the treatment development process for human diseases [1]. Thus, new veterinary treatment strategies against infectious diseases and cancer are urgently needed. Immunotherapies have shown great promise in humans, but rely on a detailed understanding of the cellular immune response, particularly of CD8^+^ cytotoxic T-lymphocytes (CTL). Such detailed knowledge does not currently exist for dogs.

Infection or neoplastic transformation of cells can activate and alter the antigen processing and presenting machinery, potentially resulting in the presentation of altered peptides on MHC class I molecules to cytotoxic CD8^+^ T-lymphocytes [2–5]. The MHC class I heavy chain (α1- α3 subunit) forms a heterotrimeric complex with beta-2-microglobulin (β_2_M) and the bound peptide [6–9]. The heavy chain in canine MHC is called DLA (dog leukocyte antigen). Seven canine MHC class I loci have been identified. Six are located on chromosome 12 and one MHC class I-like gene is linked to chromosome 18 [10, 11]. Only four of these seven genes encode functional MHC-complexes, named DLA-12, -64, -79, -88 [12]. DLA-12, -64, and -79 do not show the typical MHC class Ia characteristics, and DLA-79 is currently considered a non-classical MHC molecule [10, 12, 13]. In contrast, DLA-88 is a highly polymorphic MHC class Ia gene which most likely encodes a classical MHC molecule [13, 14]. There are 59 DLA-88 alleles known to date [13–18]. All DLA-88 alleles show high polymorphism in exons 2 and 3, which consist of constant and hypervariable regions and code for the peptide-binding groove in the α1 and α2 domains [13, 19]. The human MHC has been an active field of research for many years. There is a wide range of knowledge regarding the identification, characterization and validation of peptides and their binding specificities on MHC class I molecules [20–24]. Previous studies have demonstrated the occurrence of peptide anchoring at specific positions, as well as the existence of allele specific binding motifs [22, 25]. In contrast, little is known about the peptide binding specificities of canine MHC class I molecules.

Investigation of the canine immune system with the aim of developing or modeling immunotherapeutic interventions is an expanding field of oncology research because the occurrence of many tumors is quite similar in humans and dogs [26, 27]. Considerable sequence homologies between HLA and DLA have been identified, and the dog is an obvious candidate to be a very important model for developing new cancer therapies in human and veterinary medicine [28]. Consequently, the identification and analysis of natural and possibly altered peptides, as well as the characterization of their binding specificities on MHC class I molecules, is of fundamental importance in human and veterinary medicine. It is the prerequisite for the development of new, highly specific T cell-based immunotherapies for treating cancer [29].

In the present study, we demonstrate a detailed binding motif for a dog MHC class I molecule based on extensive analyses of 2436 nonamers out of 3720 DLA-88*50101 derived peptides identified by mass spectrometry. For this characterization, two different human cell lines expressing DLA-88*50101 heavy chain were used. This approach demonstrates a powerful tool to determine binding motifs for canine MHC class I molecules.

## Material and Methods

### Cells

C1R cells, deriving from the human B lymphoblastoid line Licr.Lon.Hym2 [30–32], as well as K562 cells descended from human proerythroblastic leukemia cells [33], were used for transfection with the canine MHC class I allele DLA-88*50101. The Epstein-Barr virus-positive (EBV) B-lymphoblastoid cell line JY [34] was used for flow cytometric analyses as well as for peptide binding assays.

### Linearization of DLA-88* Plasmid Encoded with DLA-88*50101

For linearization, 30 μg pcDNA3.1(+) vector with DLA-88*50101 coding sequence insert (GeneArt) was mixed with 5 μl NEB3.1 Buffer (New England, BioLabs), 0.5 μl 100X BSA (New England, BioLabs) and 2 μl PvuI (New England, BioLabs). HPLC-H_2_O was added to a total volume of 50 μl, followed by incubation at 37°C for 16 h. Ethanol precipitation was used to concentrate and purify the plasmid DNA: 50 μl HPLC-H_2_O, 50 μl 7.5 M NHyOAC and 300 μl 100% ethanol were added to each sample. Samples were incubated for 30 min at -20°C, followed by centrifugation at 1400 rpm for 15 min at 4°C (Centrifuge 5415D, Eppendorf). Samples were washed twice with 70% EtOH, dried at 37°C, and the linearized plasmid DNA pellet was resuspended in 16 μl Elution Buffer (Buffer EB, Qiagen) prior to spectrophotometeric analysis (NanoDrop 1000 Spectrophotometer, Peqlab).

### Transfection and Cell Culturing

K562 cells were transfected with linearized plasmid DNA containing the coding sequence of DLA-88*50101 under the control of a cytomegalovirus (CMV) promoter DLA-88*50101 via nucleofection, 1.5 x 10^6^ cells/sample were centrifuged at 300 rpm for 10 min at 4°C (Centrifuge Heraeus Multifuge 1 S-R, Thermo Fisher Scientific) and the supernatant was discarded. 270 μl SolutionV and 60 μl supplement-mix (CLB-Transfection Kit, Lonza) were mixed, and 100 μl was added to the cell pellet. 2 μg linearized plasmid DNA, containing the coding sequence of DLA-88*50101, was added prior to nucleofection (CLB-Transfection^TM^Device, Lonza, programm: cell 8 (T-016)). Cells were resuspended in 2 ml RPMI (1640 1 x, Life Technologies, 10% FCS, 1 x P/S) and transferred into a 6-well plate.

Transfection of C1R cells with linearized plasmid DNA containing the DLA-88*50101 coding sequence was conducted using Lipofectamine® 3000 Reagent (Invitrogen, Life Technologies). Prior to transfection, 2 x 10^5^ cells were centrifuged at 1200 rpm for 7 min at 4°C and resuspended in 500 μl growth medium (RPMI 1640 1 x, Life Technologies; IMDM, Lonza) without antibiotics. 1 x 10^5^ cells/well were transferred into a 24-well plate. The next day, 3 μl Lipofectamine® 3000 Reagent was diluted in 50 μl medium. A master mix of DNA was then prepared by diluting 1 μg DNA in 50 μl medium and 2 μl P3000 TM Reagent. 50 μl diluted DNA was added to each tube of diluted Lipofectamine® 3000 Reagent and after incubation for 5 min at room temperature, 50 μl/well was added to the cells. After transfection, the cells were cultivated at 37°C, 5% CO_2_ and 95% relative humidity (Heracell, 150i, Thermo Scientific). IMDM medium (Lonza, 10% FCS, 1 x P/S) was used for transfected K562 cells, and RPMI (1640 1 x, Life Technologies, 10% FCS, 1 x P/S) was used for culturing JY cells and for transfected C1R cells. 24 h post transfection, G418 was added to the medium (1mg/ml) for selecting transfected from non-transfected cells during cultivation in 96-, 48- and 24-well plates, as well as in 25 cm^2^, 75cm^2^ and 175 cm^2^ bottles. Non-transfected K562 and C1R cells were cultured in 75 cm^2^ bottles without G418 treatment. In order to select cells showing a high MHC class I expression, transfected cells were clonally expanded by limiting dilution and screened via flow cytometry.

Selected clones of K562-DLA-88*50101 and C1R-DLA-88*50101 cells were cultivated in 2 L bottles to a cell count of 1.4 x 10^9^–3.5 x 10^9^ cells. MHC class I expression of each clone was monitored on a regular basis by flow cytometry.

### Flow Cytometric Analysis and Peptide Binding Assay

For flow cytometry analysis, transfected and non-transfected cells were counted and diluted to 5 x 10^5^ cells/ml with medium. 5 x 10^5^ cells were suspended in 0.2–4 ml FACS buffer (500 ml PBS, 2.4 g BSA, 1.6 ml 0.5 M EDTA) and centrifuged at 1200 rpm for 7 min at 4°C (Centrifuge Heraeus Multifuge 1 S-R, Thermo Fisher Scientific). After discarding the supernatant, two washing steps with FACS buffer followed. The in-house produced primary antibodies BBM.1 (anti-β_2_-microglobulin, 1 mg/ml), W6/32 (Antigenic Determinant: HLA-A, B, C, 1 mg/ml), and BB7.2 (anti-HLA A2, Aw69, 1 mg/ml) were diluted 1:10 in FACS buffer, added to the cells and incubated for 30 min at 4°C. In order to analyze the auto-fluorescence of the cells, a sample without primary antibody treatment was retained in every experiment. Two washing steps with FACS buffer followed to remove unattached antibodies, after which the cells were resuspended in 100 μl FITC-conjugated F(ab‘)2-fragment goat anti-mouse IgG antibody (Jackson ImmunoResearch) which was diluted 1:100 with FACS buffer. After 20 min incubation at 4°C, cells were centrifuged at 1200 rpm for 7 min at 4°C, (Centrifuge Heraeus Multifuge 1 S-R, Thermo Fisher Scientific) followed by two washing steps. Finally, the cell pellet was resuspended in 0.2–1 ml FACS buffer and analyzed by flow cytometry (BD FACSCalibur, Becton Dickinson).

For peptide binding analysis, JY cells, as well as transfected and non-transfected C1R cells, were diluted to 2 x 10^6^ cells/ml and plated into 96-well plates using 2 x 10^5^ cells/well. 100 μl culture medium in DMSO (with 1% DMSO) for determining the auto-fluorescence of the cells, or 100 μl of each peptide dilution (5 μl peptide stock: 1 mg/ml in 100% DMSO, 495 μl medium), was added to the cells. The following FITC-labeled peptides were used: pol HIV-1 reverse transcriptase 476–484, ILK(FITC)EPVHGV [35]; S-adenosylmethionine synthase 220–227 DALK(FITC)EKVI; peptides Spatacsin 1296–1304 SVAEK(FITC)LSKL and mediator of RNA polymerase II transcription subunit28 110–118 VIK(FITC)EDVSEL; all were synthesized in house using Fmoc chemistry and verified by mass spectrometry.

After 90 min of incubation at 37°C in a 5% CO_2_ atmosphere, cells were centrifuged at 1200 rpm for 7 min at 4°C (Centrifuge Heraeus Multifuge 1 S-R, Thermo Fisher Scientific). Cells were washed twice with FACS buffer and resuspended in 500 μl FACS buffer prior to measurement via flow cytometry (BD FACS Calibur, Becton Dickinson), through which the median fluorescence of each sample was determined. For data analysis, the ratio of the samples to the negative control (peptide DALKEKVI) was calculated separately for each cell line. The ratio of the negative control DALKEKVI was subtracted from the ratio of the samples. For the determination of peptide binding on DLA-88*50101, data deriving from C1R cells were subtracted from those of C1R-DLA-88*50101 cells. For the data analysis of HLA-A*02:01 binding, the intensity of peptide binding of ILKEPVHGV on HLA-A*02:01 was set to 100%; for the analysis of DLA-88*50101 binding, SVAEKLSKL binding on DLA-88*50101 was set to 100%.

### MHC Preparation and Data Analysis

A total amount of 1.4 x 10^9^–3.5 x 10^9^ cells were harvested, after which, MHC class I molecules were isolated by using immunoaffinity purification [36]. One volume of solubilisation buffer (PBS, 1.2% CHAPS and Roche complete protease inhibitor) was added to the cell pellet. Samples were homogenized at 2000 rpm for 2 min (bench drill RB 18 Vario Rotwerk GmbH), followed by the additon of two volumes of solubilisation buffer and shaking of the sample at 200 rpm for 60 min at 4°C. After sonification (Analog Cell Disrupter 250, Branson Ultrasonics Corporation), the lysate was incubated for 1 h at 200 rpm at 4°C, and then centrifuged at maximum speed for 45 min at 4°C (Centrifuge Heraeus Multifuge 1 S-R, Thermo Fisher Scientific).

The supernatant was immunoprecipitated overnight (flow rate 2 ml/min) using the BBM.1 antibody (anti-β_2_-microglobulin, 1 mg/ml, University of Tuebingen) coupled to CNBr sepharose (GE Healthcare Europe GmbH). 8 mm I.D. Econo-columns (Bio-Rad Laboratories) were used. MHC class I complexes were eluted from CNBr columns with 0.2% TFA. For peptide filtration, the eluate was transferred into a thoroughly washed 0.5 ml 3 kDa Amicon filter (Merck Millipore Ltd.) and centrifuged at 13000 rpm (Heraeus Biofuge 13) for 15 min at 4°C. The sample was finally concentrated to a volume of 40 μl by vacuum centrifugation (Bachofer Vacuum Concentrator) and desalted using C_18_ ZipTip®pipette tips (Millipore).

All samples were subjected to three LC-MS measurements. For each replicate, 20% of the vacuum concentrated C_18_ ZipTip eluate was loaded on a C_18_ 2 cm x 75 μm trapping column (Thermo Fisher Scientific). Peptides were separated on a 25 cm x 50 μm nano-HPLC column with C_18_ beads of 2 μm diameter and 10 nm pore size (Thermo Fisher Scientific), using a 90 min gradient ranging from 2.4% to 32% CH_3_CN in 0.15% formic acid. An UltiMate 3000 RSLCnano nano-HPLC system coupled online to an LTQ Orbitrap XL mass spectrometer (both Thermo Scientific) was applied. Parent ions were recorded in the range from 400 Th to 650 Th at a resolution of 60,000 (at m/z = 400 Th). The five most abundant doubly and triply charged precursors from each MS1 scan were selected for collision-induced dissociation and dynamically excluded from repeated fragmentation for 3 s. Fragment ions were recorded in the linear trap quadrupole.

MS data were analysed using the ProteomeDiscoverer version 1.4 (Thermo Scientific) as a search engine for peptide identifications [37]. The search was performed against the 20,279 human proteins of the Swiss-Prot database (www.uniprot.org, release 27 September, 2013) and against a separate decoy database consisting of the reversed forms of these proteins. The allowed mass errors were 5 ppm for MS1 and 0.5 Da for MS2. Oxidation of methionine (+15.99492 Da) was set as variable modification. Peptide lists obtained from experiments 2–4 were filtered to contain only 8- to 12-mers with a Mascot score ≥20 and to meet a false discovery rate of 5% as determined by Percolator [38]. To allow for a less biased analysis of the peptide length distribution presented by DLA-88*050101, experiment 1 was filtered to a target FDR of 5% without peptide length restriction.

To determine the DLA-88*50101 binding motif, the analysis of nonamers with regard to the homology of amino acids at specific positions was conducted using an in-house excel data sheet as well as Weblogo (http://weblogo.berkeley.edu/logo.cgi).

### Alignments

For the comparison of DLA-88*50101 and HLA-A*02:01 amino acid sequences, as well as certain residues within the binding pockets, the following reference sequences were used: HLA-A*02:01 (AAA76608.2, [39–41]) and DLA-88*50101 (AF100577, AF101496, [19]). The binding motif of HLA-A*02:01 was previously published [42, 43] and the web illustration at NetMHC-4.0 Server was modified by S. Barth.

For the comparison of HLA-A*02:01 with other species, the following sequences were used: human (*Homo sapiens;* AAA76608.2), tree shrew (*Tapaia belangeri;* AFM54604.1), giant panda (*Ailuropoda melanoleuca;* ABY27206.1), donkey (*Equus asinus;* ADE61511.1), horse (*Equus caballus;* BAI45218.1), wild pig (*Sus scrofa;* ACA33856.1), black rhinoceros (*Diceros bicornis minor;* AAD02690.1), porpoise (*Neophocaena asiaeorientalis;* ALB25881.1), cow (*Bos taurus;* AFS51741.1), dog (*Canis lupus familiaris;* NP_001014767.1), sheep (*Ovis aries;* AGN98174.1), and cat (*Felis catus;* ACK99133.1).

## Results

### Transfection of Human Cells with DLA

**Fig 1:**
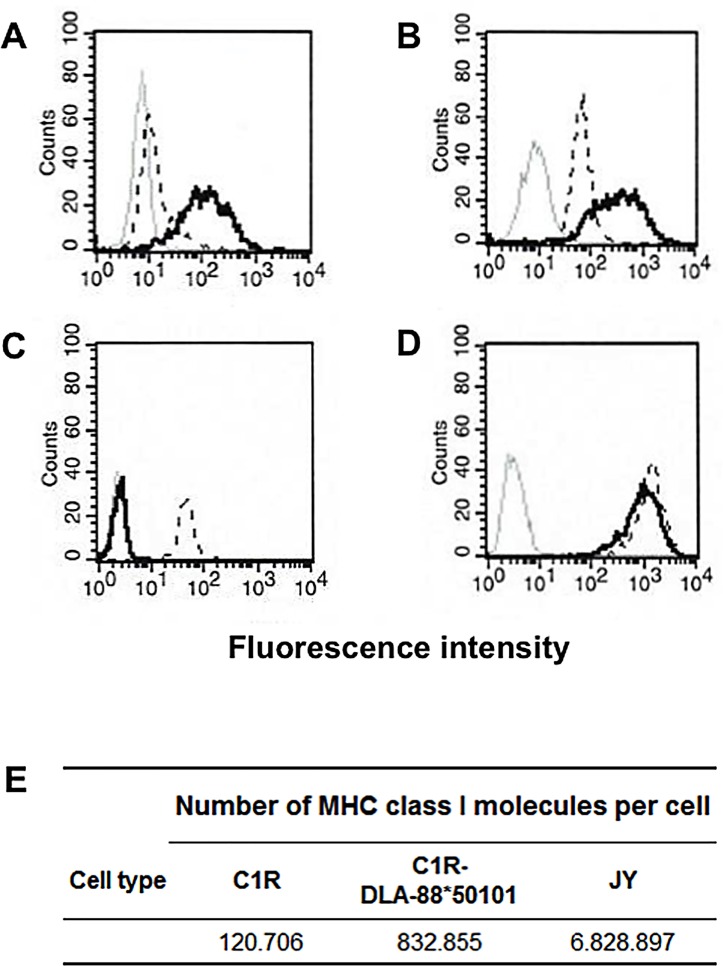
K562 and C1R cells expressing DLA-88*50101. DLA and HLA class I expression of transfected and non-transfected K562 **(A)** and C1R cells **(B)** was determined by flow cytometry using BBM.1 antibody (anti-β_2_-microglobulin). Staining with W6/32 antibody (anti-HLA-A, -B, -C) and BB7.2 (anti HLA-A2,) characterizes the HLA expression of C1R **(C)** and JY cells **(D)**. **(A)** non-transfected K562 cells unstained (grey line); non-transfected K562 cells stained with BBM.1 antibody (black dotted line), and transfected K562 cells stained with BBM.1 antibody (black line). **(B)** non-transfected C1R cells unstained (grey line), non-transfected C1R cells stained with BBM.1 antibody (black dotted line), and transfected C1R cells (black line) stained with BBM.1 antibody. **(C)** non-transfected C1R cells unstained (grey line), C1R cells stained with W6/32 antibody (black stippled line), or stained with BB7.2 antibody (black line). **(D)** JY cells unstained (grey line), stained with W6/32 antibody (black dotted line), or stained with BB7.2 antibody (black line). **(E)** MHC class I expression on JY cells, DLA-88*50101 transfected and non-transfected C1R cells. QIFIKIT (Dako) and BBM.1 antibody were used in flow cytometry for absolute quantification of MHC class I molecules per cell.

In order to determine the MHC class I binding motif of DLA-88*50101, human C1R and K562 cells were transfected with DLA-88*50101 heavy chain. Stable transfection was controlled by flow cytometric analysis using the human anti-β_2_M antibody (Fig 1A, Fig 1B). The use of an anti-human β_2_M antibody allows the simultaneous detection and immunoaffinity-based extraction of human and canine MHC, since only the canine MHC heavy chain is transfected and pairs with the human β_2_M light chain naturally expressed in these cell lines. Non-transfected K562 cells do not express any human MHC (Fig 1A black dotted line). C1R cells express HLA-B*35:03 and HLA-C*04:01 detected by anti-human β_2_M antibody (Fig 1B black dotted line) [22, 30]. When DLA-88*50101 transfected human K562 (Fig 1A black line) or C1R (Fig 1B black line) cells were analyzed, an increased fluorescence intensity could be detected. Comparing the normalized HLA and DLA class I expression of C1R cells to MHC I/II ^high^ JY cells, we observed a 6.9-fold increase of DLA-88*50101 expression (roughly 830,000 MHC class I molecules/cell) on transfected compared to non-transfected C1R cells (roughly 120,000 MHC class I molecules/cell). Although DLA-88*50101 expression on C1R cells was 8.2-fold lower than HLA-expression on JY cells (Fig 1E), it proved sufficient for further analyses.

### Characterization of the MHC Class I Binding Motif

In order to identify the peptide binding motif of DLA-88*50101, C1R or K562 cells stably transfected with DLA-88*50101 were used for MHC-immunoprecipitation and the eluted peptides were identified by tandem mass spectrometry. A total of four MHC-preparations were performed: three using C1R transfected cells and one using K562 transfected cells (Table 1). A total of 2436 nonamers out of 3720 DLA derived peptides were used to analyze the binding motif (S1 Table). Peptides presented by HLA-B*35:03 (0.4%) and HLA-C*04:01 (5.9%) that are co-expressed on C1R cells were subtracted (as annotated by NetMHC-3.4 and SYFPEITHI) from the analyses (Table 1).

**Table 1:** Summary of MHC class I immunoaffinity purification assay of C1R and K562 cells transfected with DLA-88*50101.

Exp.	Transfectant	Cell count	Peptides^a^	Peptides^b^ HLA / DLA	%HLA-B*35:03^c^ SYFPEITHI / NetMHC-3.4	%HLA-C*04:01^c^ SYFPEITHI / NetMHC-3.4	%Duplicates^d^	Peptides^e^ (DLA)
1	C1R-DLA-88*50101	1.9 x 10^9^	1184	73 / 1093	0.8	5.4	4.8	1036
2	C1R-DLA-88*50101	2.1 x 10^9^	1095	94 / 1001	0.2	8.4	5.5	941
3	C1R-DLA-88*50101	3.5 x 10^9^	1214	97 / 1117	0.4	7.6	5.4	1051
4	K562-DLA-88*50101	1.4 x 10^9^	709	- / 709	-	-	2.4	692

^a^The total number of peptides identified. Peptides >13 residues in lenghth (*n* = 18) were not considered in further analyses.

^b^The number of identified peptides eluted from human and canine MHC class I molecules.

^c^Percentage of peptides deriving from HLA-B*35:03 and HLA-C*04:01 molecules was predicted by Ligandosphere using SYFPEITHI (data >50% of allotype-specific maximal score) and NetMHC-3.4 (IC_50_ <500nM).

^d^Percentage of deleted peptide duplicates originating from methionine residues of different oxidation states.

^e^The total number of peptides deriving from DLA-88*50101. Duplicates within each experiment were substracted.

**Fig 2:**
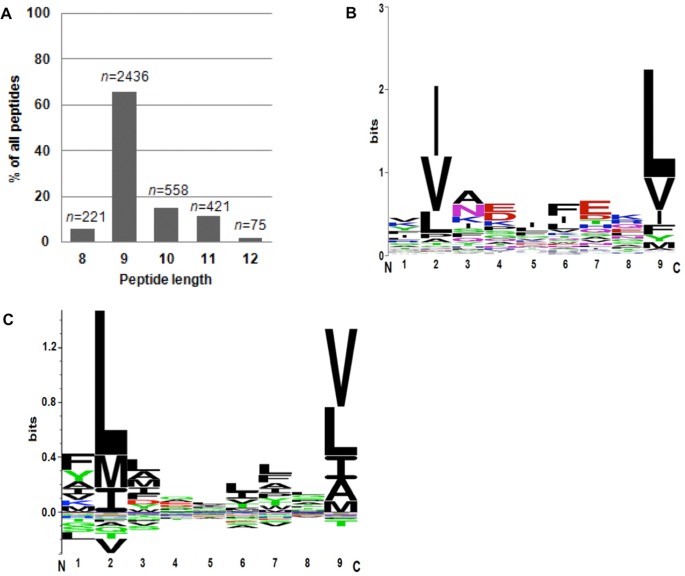
The MHC binding motif of peptides deriving from C1R-DLA-88*50101 and K562-DLA-88*50101 cells. **(A)** The average length distribution of MHC peptide ligands deriving from C1R and K562 cells transfected with DLA-88*50101 shows that the majority of peptides are 9 residues in length (*n* = 2436; 65.5%). **(B)** Summary binding motif of DLA-88*50101. Nonamers deriving from C1R-DLA-88*50101 cells, (Exp. 1–3) as well as peptides eluted from K562-DLA-88*50101 cells (Exp. 4), were characterized and revealed dominant anchor residues at position 2 and 9. **(C)** Binding motif of HLA-A*02:01. Derivation of data is described in the “Materials and Methods” section.

Comparing the peptide length distributions of all four experiments, the majority (65.5%) of the peptides were nonamers. This is consistent with the canonical size of most human and non-human primate MHC class I ligands characterized to date (Fig 2A). A detailed analysis of the length distribution from each experiment separately indicated no differences in the length distribution of peptides derived from transfected C1R cells compared to transfected K562 cells (S1 Fig). Peptides of 8 (5.9%), 10 (15.0%), 11 (11.3%), and 12 (2.0%) amino acid residues were also identified, but with lower frequencies. In experiment 1, we demonstrated that peptides with 13 or more amino acid residues were of even lower abundance than the 12-mers (S1A Fig).

When analyzing nonamers for a possible binding motif, the majority of peptides contained a cluster of amino acids with the aliphatic, hydrophobic residues isoleucine (I = 41.4%), leucin (L = 13.0%), and valine (V = 32.4%) in position 2. A similar cluster of aliphatic, hydrophobic amino acids was found at the C-terminus: leucine (L = 58.1%) and valine (V = 17.6%). Since no other positions presented an increased frequency of particular amino acids or groups of amino acids, these data suggest that positions 2 and 9 serve as the main anchors conferring DLA-88*50101 binding specificity. Other notable, but less distinct patterns were noted in positions 5 and 6, and again, amino acids with hydrophobic residues clustered in these positions (Table 2). A separate analysis of amino acid frequencies in each of the four individual experiments showed no differences between transfected C1R and K562 cells (S2 Table). Taken together, these data delineate a putative binding motif for DLA-88*50101, characterized by a predominant length of 9 residues, and anchored by hydrophobic residues I,L,V in position 2, and L and V at the C-terminus (Fig 2B).

**Table 2:** Quantitative binding motif for DLA-88*50101.

Position									
Residue	1	2	3	4	5	6	7	8	9
A	5.42	2.59	21.06	5.13	4.15	3.00	5.62	5.67	1.35
C	0.08	0.08	0.00	0.04	0.00	0.00	0.00	0.04	0.00
D	0.49	0.33	0.37	16.17	4.27	0.86	8.91	0.86	0.04
E	1.19	0.53	1.31	17.24	4.39	3.41	26.15	10.26	0.37
F	9.48	0.70	7.02	1.48	11.82	24.34	1.31	5.17	5.54
G	2.87	0.53	2.18	7.22	7.22	2.55	1.48	6.98	0.16
H	2.30	0.04	2.71	1.31	2.75	2.09	8.05	3.16	0.12
I	8.74	***41*.*42***	8.33	2.26	16.05	13.75	3.20	0.70	7.51
K	11.21	0.45	9.98	9.89	6.03	5.05	2.42	12.73	0.45
L	6.73	***13*.*01***	2.26	1.97	13.18	11.70	3.74	7.92	***58*.*05***
M	2.75	0.70	2.22	0.21	2.18	1.97	0.82	1.15	3.53
N	1.03	0.12	18.68	3.86	3.90	3.33	4.80	7.47	0.45
P	0.33	2.96	0.62	8.74	2.01	2.42	1.77	0.53	0.21
Q	3.82	0.90	6.49	5.62	3.00	3.98	7.96	10.43	0.29
R	6.65	0.37	0.94	3.78	2.26	2.30	2.01	10.67	0.25
S	6.57	1.15	7.10	7.55	2.26	3.57	6.49	5.99	0.12
T	4.76	1.60	2.87	3.90	3.20	4.35	8.09	4.15	0.21
V	12.40	***32*.*39***	2.18	2.91	6.28	6.81	5.38	2.01	***17*.*61***
W	2.13	0.04	0.08	0.21	1.93	3.49	0.25	0.00	0.00
Y	11.04	0.08	3.61	0.49	3.12	1.03	1.56	4.11	3.74

Analysis of the nonamers characterized in experiments 1–4 with regard to the homology of amino acids at specific positions. A total of *n* = 2436 nonamers were isolated as described in the “Materials and Methods” section, and primary anchor positions were defined.The most preferred residues for the anchor positions are highlighted in italic bold type.

### Comparison of DLA-88*50101 with HLA-A*02:01

**Fig 3:**
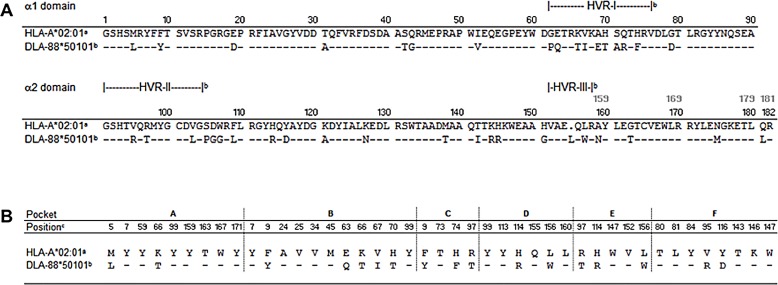
Alignment of MHC class I α1 and α2 domain amino acid sequences of HLA-A*02:01 and DLA-88*50101. **(A)** Alignment of MHC class I α1 and α2 domain amino acid sequences of human (HLA-A*02:01; AAA76608.2^a^) and dog (DLA-88*50101; AF100577, AF101496, [19]^b^). Identity of amino acid sequences to human HLA-A*02:01 is shown by dashes, disposability of data is illustrated by a dot. Position numbers for DLA-88*50101 are indicated in black above the sequences. Deviating position numbers for HLA-A*02:01 resulting from the amino acid deletion are given in grey bold type on top of the corresponding DLA-88*50101 position. Residues allocated to hypervariable regions I-III are indicated above the alignment ([19]^b^). **(B)** Comparison of selected residues of HLA-A*02:01 (AAA76608.2^a^) and DLA-88*50101 (AF100577, AF101496, [19]^b^) with reference to pockets A-F. The indicated positions refer to HLA-A*02:01 ([39–41]^c^). For DLA-88*50101, corresponding positions are shifted by one amino acid starting with the C-terminal of position 154, due to an amino acid insertion [44].

The DLA-88*50101 binding motif reveals a striking similarity to the peptide binding motif of HLA-A*02:01 (Fig 2C). Thus, to exclude the presence of HLA-A*02:01 on C1R cells, we performed flow cytometric analysis with a HLA-A*02:01 specific antibody, demonstrating no expression of HLA-A2 on C1R cells (Fig 1C) compared to HLA-A*02:01 positive JY cells (Fig 1D). This is consistent with previous results [22, 30]. To further investigate the basis of the similarities of the two binding motifs, we next performed an amino acid sequence alignment of the MHC class I α1 and α2 domains of HLA-A*02:01 and DLA-88*50101. The overall amino acid sequence homology of HLA-A*02:01 and DLA-88*50101 is 79%, with a slight tendency of amino acid changes to cluster in the hypervariable regions (Fig 3A).

Specific pockets in the MHC alpha chain have a key role in mediating peptide anchoring. For HLA-A*02:01, six pockets have been identified (A-F). Pockets B and F are important for P2 and P9 anchor residues, respectively. Thus, in addition to comparing the amino acid sequences of complete MHC class I α1 and α2 chains, a comparison of selected residues within the binding pockets is of special relevance. Regarding the DLA F pocket, HLA-A*02:01 and DLA-88*50101 differ by only two amino acid changes (V95R; Y116D); the C-terminal amino acid of the peptide anchors in this pocket. The B pocket consists of 11 amino acids, of which five positions are altered in DLA compared to HLA (F9Y; E63Q; K66T; V67I; H70T). Further differences were detected in the other pockets (Fig 3B). These results demonstrate similarities between DLA-88*50101 and HLA-A*02:01 regarding the amino acid sequences responsible for building the pockets, but they also highlight distinct differences.

### Comparison of HLA-A*02:01 Alpha Chains with Other Species

The question that arises from the DLA-88*50101 amino acid sequence analysis discussed above is whether the degree of amino acid homology to HLA-A*02:01 is also found for other animal species, or whether this is exclusive to canine MHC, potentially based on selective pressures shaped by the long record of human-canine co-evolution. We conducted an alignment of MHC class I α1 and α2 domain amino acid sequences of a number of species that showed a high homology to HLA-A*02:01 according to NCBI (BLASTP). Primates and unclassified-primates were excluded from the selection.

**Fig 4:**
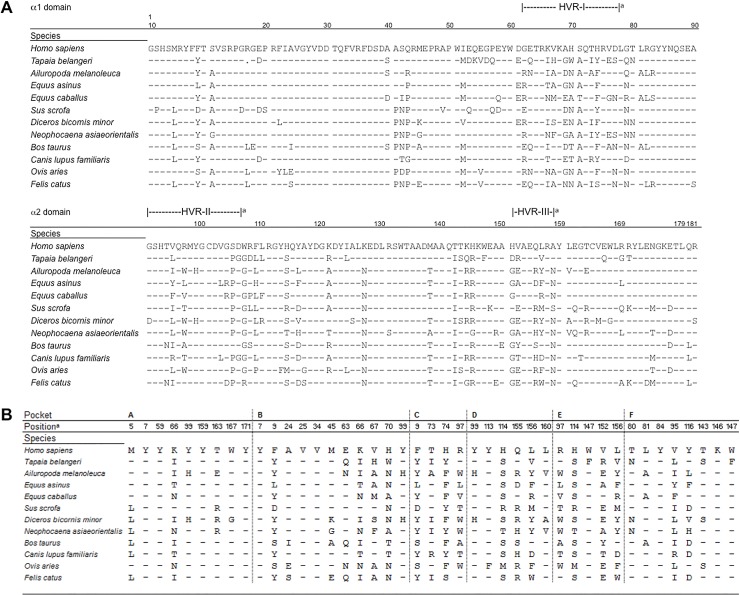
Alignment of MHC class I α1 and α2 domain amino acid sequences of selected species. **(A)** Alignment of MHC class I α1 and α2 domain amino acid sequences of human (*Homo sapiens;* AAA76608.2), tree shrew (*Tapaia belangeri;* AFM54604.1), giant panda (*Ailuropoda melanoleuca;* ABY27206.1), donkey (*Equus asinus;* ADE61511.1), horse (*Equus caballus;* BAI45218.1), wild pig (*Sus scrofa;* ACA33856.1), black rhinoceros (*Diceros bicornis minor;* AAD02690.1), porpoise (*Neophocaena asiaeorientalis;* ALB25881.1), cow (*Bos taurus;* AFS51741.1), dog (*Canis lupus familiaris;* NP_001014767.1), sheep (*Ovis aries;* AGN98174.1) and cat (*Felis catus;* ACK99133.1). Identity of residues to human HLA-A*02:01 (*Homo sapiens;* AAA76608.2) is shown by dashes, disposability of data by dots. Residues allocated to hypervariable regions I-III are indicated above the alignment ([19]^a^). **(B)** Same as **(A)** but focusing on the MHC class I residues with reference to pocket A-F. The indicated positions refer to HLA-A*02:01 [39–41]^a^).

The highest amino acid sequence homologies from the non-primate animals aligned to HLA-A*02:01 were found for the donkey (*Equus asinus*, 81%), dog (*Canis lupus familiaris*, *80%*), giant panda (*Ailuropoda melanoleuca*, 79%), horse (*Equus caballus*, 78%), and tree shrew (*Tapaia belangeri*, 76%), as well as for the wild pig (Sus scrofa), porpoise (*Neophocaena asiaeorientalis*), cow (*Bos taurus*) and sheep (*Ovis aries*). Similarily, 72% homology to HLA-A*02:01 was reported for the cat (*Felis catus*). Again, the majority of the amino acid changes occur in the hypervariable regions (Fig 4A).

Comparing selected residues that constitute the HLA-A*02:01 peptide binding pockets revealed rather few changes in the F pocket from the selected non-primates. The C-terminus of the peptide binds to the F pocket (Fig 4B). The majority of amino acid substitutions in all species occur in pocket C; there are fewer changes in pockets A and F in comparison to HLA-A*02:01. There are certain positions where none of the selected species possess amino acids identical to HLA-A*02:01: pos. 9 in pockets B and C, pos. 70 in pocket B, pos. 74 in pocket C, pos. 95 in pocket F and position 114 in pockets D and E. Position 9 in pockets B and C is of fundamental importance for peptide binding and CTL recognition [41, 45]. At this position, 7 out of 12 selected species possess a Y instead of F (F9Y). Taken together, high similarities of the amino acids responsible for building the pockets are not only found between HLA-A*02:01 and DLA-88*50101, but also in other species, arguing against a preferred co-evolution of MHC molecules of humans and dogs.

### Binding Analysis of HLA-A*02:01 Derived Peptides into DLA

Having demonstrated the similar binding motifs of HLA-A*02:01 and DLA-88*50101, we now questioned whether peptides known as strong ligands for HLA-A*02:01 would also fit into the DLA-88*50101 binding groove. Pulsing JY cells that are homozygous for HLA-A*02:01 with FITC-labeled peptide ILKEPVHGV (HIV p24) revealed strong fluorescence intensity, which was set to 100% for comparison purposes. ILKEPVHGV is known to be a strong binder to HLA-A*02:01 with a SYFPEITHI score of 30, representing 83% of the optimal binding score (Table 3). The FITC-labeled DLA-88*50101 ligands SVAEKLSKL (75% normalized binding score to HLA-A*02:01) and VIKEDVSEL (72% normalized binding score to HLA-A*02:01) revealed 38% and 52% normalized fluorescence intensity on JY cells, indicating weak binding. FITC-labeled DALKEKVI, a ligand of HLA-B*51, showed no binding and served as a negative control.

**Table 3:** Bioinformatic MHC binding scores for peptides used for experimental binding analysis into HLA and DLA.

Peptide	Score HLA-A*02:01	% of max. Score HLA-A*02:01 (n = 36)	Score DLA-88*50101	% of max. Score DLA-88*50101 (n = 30)	%Rank HLA-A*02:01	%Rank HLA-B*35:03	%Rank HLA-C*04:01
	SYFPEITHI@home	SYFPEITHI@home	SYFPEITHI@home	SYFPEITHI@home	NetMHCpan-3.0	NetMHCpan-3.0	NetMHCpan-3.0
ILKEPVHGV	30	83	19	63	0.7	34.0	10.0
DALKEKVI*	-	-	-	-	90.0	49.0	90.0
SVAEKLSKL	27	75	26	86	4.0	7.0	3.5
VIKEDVSEL	26	72	25	83	7.5	6.5	6.5

Peptides used for binding analysis of HLA-A2 derived peptides into DLA (ILKEPVHGV, DALKEKVI, University of Tuebingen). Scores for peptides deriving from DLA-88*50101 selected for peptide synthesis (SVAEKLSKL, VIKEDVSEL, University of Tuebingen) and subsequent peptide binding analysis.

* For HLA-A*02:01, there is no matrix available for octamers with reference to SYFPEITHI@home.

**Fig 5:**
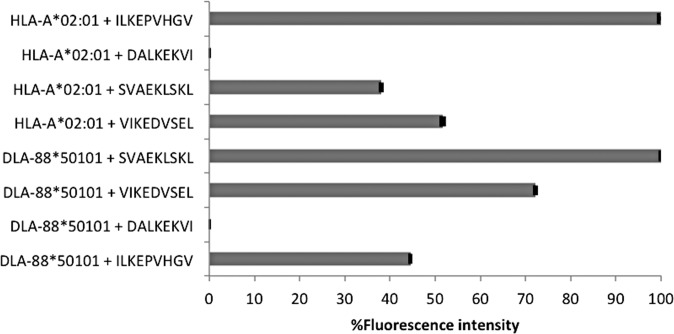
Peptide binding on HLA-A*02:01 versus DLA-88*50101. The cells were incubated with peptides pol HIV-1 reverse transcriptase 476–484, ILKEPVHGV; S-adenosylmethionine synthase 220–227 DALKEKVI; Spatacsin 1296–1304 SVAEKLSKL and mediator of RNA polymerase II transcription subunit28 110–118 VIKEDVSEL for 1 h 30 min at 37°C in a 5% CO_2_ atmosphere. DMSO was used as negative control to determine the intrinsic fluorescence. Samples were analyzed by flow cytometry and data derived from two biological experiments and three technical replicates was analyzed as described in the “Materials and Methods”. The standard deviations of the analyzed data are shown in bars. For HLA-A*02:01 loading, the fluorescence intensity of peptide binding regarding ILKEPVHGV on HLA-A*02:01 was set to 100%, whereas for DLA-88*50101 loading, SVAEKLSKL binding on DLA-88*50101 was set to 100%.

When DLA-88*50101 was pulsed with these FITC-labeled peptides, the DLA-88*50101 ligand SVAEKLSKL (86% normalized SYFPEITHI binding score for DLA-88*50101) showed the strongest fluorescence intensity, which was again set to 100%. The normalized fluorescence intensity of VIKEDVSEL (83% normalized SYFPEITHI score for DLA-88*50101) was 72% when compared to SVAEKLSKL. The fluorescence intensity of the HLA-A*02:01 ligand ILKEPVHGV (63% normalized SYFPEITHI score for DLA-88*50101) was less than 50% and no binding was detected when cells were pulsed with HLA-B*51 specific peptide DALKEKVI, which served as a negative control (Fig 5). These results strongly indicate allotype-specific binding of peptides derived from HLA-A*02:01 and DLA-88*50101, in spite of the fact that bioinformatic epitope predictions showed high binding scores for both, suggesting cross-specific binding.

## Discussion

The importance of cancer immunotherapies has increased dramatically in recent years [46, 47]. Nevertheless, a crucial problem is that animal models are limited in their relevance for humans. Inbred mouse strains, or “artificial” induced disease animal models are not able to mirror human tumor growth over a long period of time. Neither characteristics of minimum residual disease nor heterogeneous macro- and microenvironment can be addressed as in spontaneously occurring human cancer. A more suitable animal model might be the domestic dog, where cancer has increased in recent years due to an increased life expectancy [48]. Moreover, tumor initiation and progression in the dog are influenced by similar factors as in humans, including age, nutrition, sex, reproductive status and environmental exposures [49–51]. Dogs are relatively outbred compared with laboratory animals, and the inclusion of dogs from different breeds in investigations could provide a value higher than that achieved in studies of inbred laboratory animals. They not only develop a similar range of naturally occurring cancers as humans, they also share deadly features with human cancers, such as resistance to therapy, recurrence, and metastasis [26]. Thus, all of these features of canine cancer can uniquely contribute to our understanding of cancer pathogenesis, progression, and therapy, and make the pet dog a very interesting model for cancer therapy [26]. Until now, however, the dog has not been suitable for T cell immunotherapy because only a small amount of data has been available on canine MHC binding motifs, a prerequisite for this kind of therapy. In the current work, we present for the first time the exact peptide-binding motif for the major DLA allotype DLA-88*50101, which demonstrates similarities to the binding motif of HLA-A*02:01.

At this time, it is known that among classical dog MHC class I proteins, DLA-88 is polymorphic and represents 59 alleles [13–18]. We investigated the binding motif of DLA-88*50101, which is common to the popular Boxer and Golden Retriever breeds [18]. Using two human cells lines (C1R and K562) transfected with DLA-88*50101, we were able to isolate 3720 peptides from DLA-88*50101. Peptide elution from MHC molecules was performed after MHC immunoprecipitation. Unfortunately, no currently available antibodies detect DLA. We took advantage of the fact that the MHC in the transfected cells harbored human β_2_M, since only the DLA alpha chain was transfected. Thus, the human β_2_M antibody BBM.1 [52] was successfully implemented for MHC immunoprecipitation. A possible pitfall of the utilized system would be the fact that we made use of the human antigen processing machinery instead of using canine cells. A species-specific proteolytic and peptide transporter machinery could influence the selection of peptides presented by MHC molecules at the cell surface. The following three aspects might be especially relevant in our setting: (1) certain predispositions for generating peptides that are enriched for particular hydrophobic or basic residues at the C-terminal anchor positions, (2) differential N-terminal trimming, and (3) a bias towards peptides of certain lengths. The human antigen processing machinery generates a wider repertoire of C-terminal peptide residues in the ER than, for example, the mouse antigen processing machinery [53, 54]. Consequently, it should be suitable for establishing the binding motifs of DLAs.

Nonamers represent the majority of eluted peptides (65.5%). These peptides were used for peptide alignment and frequency matrix generation to determine the binding motif (Table 2). At the C-terminus hydrophobic amino acids Leu (58.1%) Val (17.6%) were dominant. These two amino acids were found at this position in 75.7% of all peptides. Peptides with basic residues at the C-terminus had a frequency of less than 1% (Arg 0.3%; His 0.1%; Lys 0.5%; Table 2). This clearly demonstrates an absolute and restrictive dominance of DLA-ligands with aliphatic, hydrophobic residues at the C-terminus, even though the presence of the human proteasome and transporter associated with antigen processing (TAP) would fulfill the requirement to load peptides with basic residues at the C-terminus.

Ross and colleagues suggested a possible binding motif for DLA-88*50801. Their analysis was based on sequence comparison of 36 peptides and modeling analyses [55]. They proposed positions 2 and 3 as preferred hydrophobic residues, while the C-terminal residue of the peptide preferred a basic amino acid. We believe that it is worth performing investigations using the transfection model described here to further explore the binding motif of this DLA-88 allele.

The binding motif of DLA-88*50101 is strikingly similar to the binding motif of human HLA-A*02:01. Thus, we first verified that the human cells used for transfection did not express HLA-A*02:01 (Fig 1C). This was also supported by published data [22, 30]. Next, we performed an alignment of the amino acid sequence of the HLA-A*02:01 peptide-binding groove α1 and α2 domains with different non-primate species to investigate a general homology of this part of the MHC molecule. The homology of DLA-88*50101 and HLA-A*02:01 was only 79% in this area, leading us to specifically focus on the homology of the amino acids in the binding pockets. In the F pocket, positions 95 and 116 harbor highly polymorphic residues (Fig 3B). Two changes were found in DLA-88*50101 compared to HLA-A*02:01 (V95R and Y116D). It is unlikely that changes of these two residues would lead to weaker CTL-recognition [41]. Comparison of the B pocket revealed a more heterogenic picture among different species. DLA-88*50101 compared to HLA-A*02:01 demonstrated five substitutions (F9Y, E63Q, K66T, V67I and H70T). All positions are known to be important for peptide binding [41]. In addition, the crystal structure of DLA-88*50801 shows that the Leu insertion of residue 154, which also exists in DLA-88*50101, does not influence the peptide binding [44]. From this data it was therefore not clear whether DLA-88*50101 and HLA-A*02:01 would share the same peptides based on a similar binding motif. A rationale for this hypothesis may be the co-evolution dog and human since the beginning of domestication, suggested by molecular dating to be between 18,800 to 32,100 years ago, [56]. Note that recent evidence suggests that East Asian and Western Eurasian dogs might have been domesticated separately 14,000 to 6,400 years ago [57]. Since both species were exposed to the same pathogens, one might presume such a phenomenon based on immunological co-evolution [58]. This view is supported by high sequence homology, not only of humans and dogs, but also of human MHC class I with other non-primate mammals. The results of our peptide binding assays, however, did not support this hypothesis, since a well-established HLA-A*02:01 binding peptide showed weak binding on DLA-88*50101. In this line, peptides known as ligands for DLA-88*50101 featured weak binding on HLA-A*02:01. One explanation for this might be the different amino acid in pocket F at position 166 (Y116D). Aspartic acid at this position creates a larger space to accommodate the large amino acids Y or F, which are not found as Pc anchors for HLA-A*02:01. In addition, the mutation F9Y in pocket B might also be responsible for our observation, because it dramatically decreases the peptide binding capacity [41, 45]. Position 9 in pocket B is located in the bottom of the binding groove of HLA-A*02:01, and might therefore have a strong influence on the P2 anchor position of the bound peptide [9, 45].

The present report is the first to identify a DLA-88 binding motif in detail based on the analysis of 3720 peptides eluted from DLA-88*50101. The primary objective of this study was to gain a better understanding of the canine cellular immune response by identifying DLA binding motifs. This will aid the future development of vaccines against canine infectious diseases and T cell-dependent immunotherapies against canine cancer, as well as the development of the pet dog as a useful animal model. The strategy presented for the identification of binding motifs proved to be a powerful tool for the characterization of MHC allotypes, and thus may be of general interest for immunological research across species boundaries.

## Supporting Information

S1 Fig Peptide length distribution of peptides with reference to experiments 1–4. C1R-DLA-88*50101 experiments 1–3 (A-C); length distribution of peptides deriving from K562-DLA-88*50101 (D).
(TIFF)

S1 Table Sequences of peptides deriving from DLA-88*50101 with reference to experiments 1–4. (DOCX)

S2 Table Quantitative binding motif for DLA-88*50101 with reference to experiments 1–4 (A-D) (DOCX)
