# Supplementary figures and images for: Characterization of the Canine MHC Class I DLA-88*50101 Peptide Binding Motif as a Prerequisite for Canine T Cell Immunotherapy
Source: PLoS One. 2016 Nov 28;11(11):e0167017. doi: 10.1371/journal.pone.0167017 (PMC5125661; doi:10.1371/journal.pone.0167017)

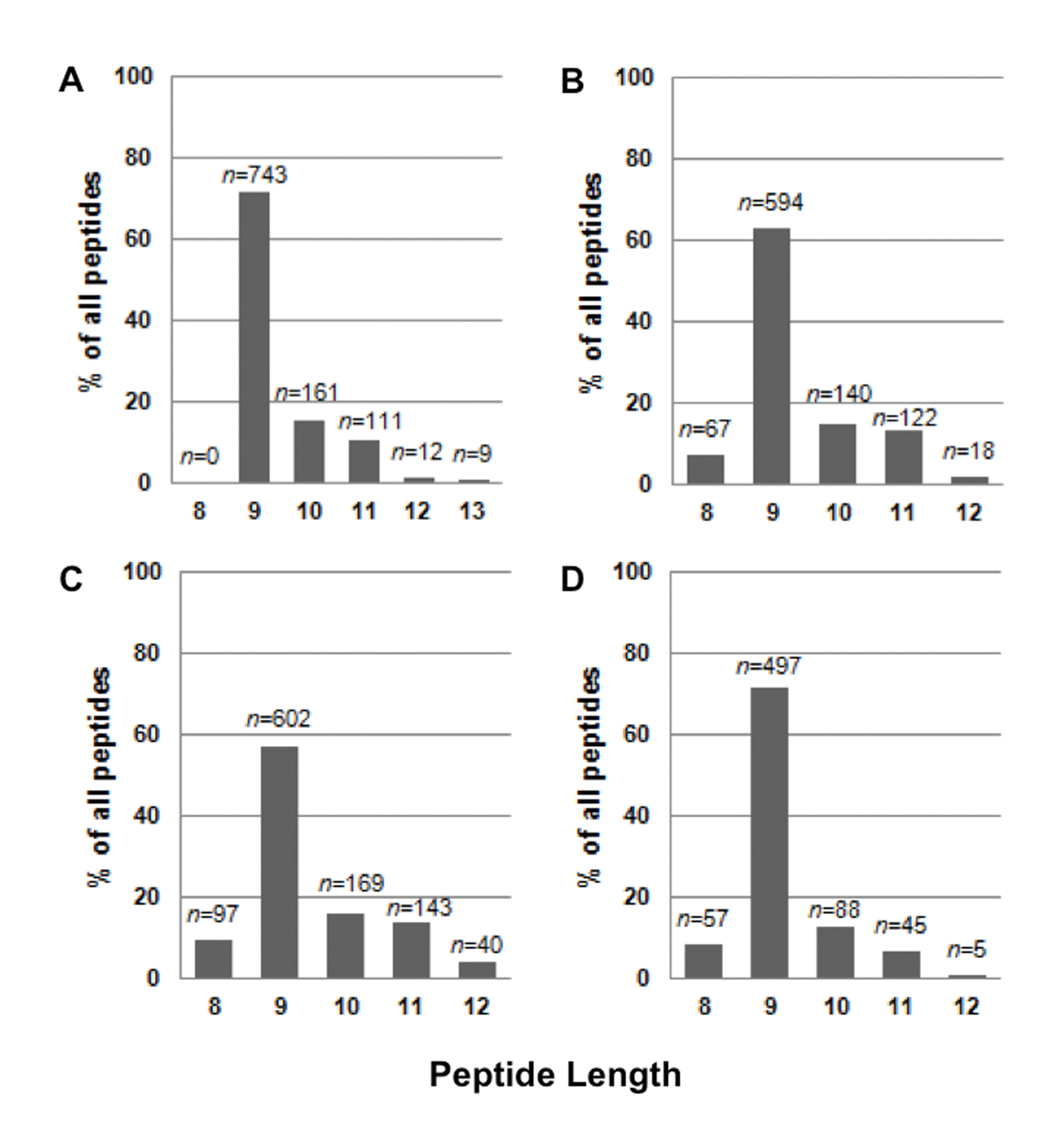

Supplement: S1 Fig — C1R-DLA-88*50101 experiments 1–3 (A-C); length distribution of peptides deriving from K562-DLA-88*50101 (D). (TIFF) [file pone.0167017.s001.tiff]
