# Supplementary material for: Characterization of the Canine MHC Class I DLA-88*50101 Peptide Binding Motif as a Prerequisite for Canine T Cell Immunotherapy
Source: PLoS One. 2016 Nov 28;11(11):e0167017. doi: 10.1371/journal.pone.0167017 (PMC5125661; doi:10.1371/journal.pone.0167017)
Supplement: S1 Table — (DOCX) [file pone.0167017.s002.docx]

|  | **Peptide length** | | | | | |
| --- | --- | --- | --- | --- | --- | --- |
|  | **8** | **9** | **10** | **11** | **12** | **13** |
| Total | *n=221* | *n=2436* | *n=558* | *n=421* | *n=75* | *n=9* |
| No. |  |  |  |  |  |  |
| 1 | AAVLNKEE | ADFGLSRNI | AAMLDTVVFK | ADIDGDGQVNY | ADVYRDGTGVVE | DRPARHPQEQPLW |
| 2 | ADEFGYSR | ADLRKTLIL | AAVAIKAMAK | AIFGATGQTGL | ADVYRDGTGVVE | ESVGKGAVHDVKD |
| 3 | ADEFGYSR | AFADIIHSL | AAVAIKAMAK | AINVEQAFQTI | AINQQTGAFVEI | GLRSFSGIKGLQL |
| 4 | ADKNVPNL | AFKTEWEAL | AFFSKTWIEV | AINVEQAFQTI | AINQQTGAFVEI | GSGSGWSSSRGPY |
| 5 | AINSISKL | AIAEAWARL | AFVQPGPVSL | AINVEQAFQTI | AKDVKFGADARA | KEIKILNIFGVIK |
| 6 | AINSISKL | AIAEAWARL | AGDDAPRAVF | AIVEAGGMQAL | AKDVKFGADARA | PEPAKSAPAPKKG |
| 7 | AINSISKL | AIAEFARSL | AGDDAPRAVF | AKADGIVSKNF | ALTNGIYPHKLV | PGLARQAPKPRKQ |
| 8 | AIRNDEEL | AIAPIIAAV | AHESVVKSED | AKHAVSEGTKA | APPGKPQGPPQQ | RIYDYDPLYKEAL |
| 9 | ASRMEEVD | AIAPIIAAV | AHESVVKSED | AKVDATEESDL | ATTEDAQAAVDY | SPPGKPQGPPPQG |
| 10 | ATVEDYLH | AIAPIIAAV | AHLDNLKGTF | ALTNGIYPHKL | AYQKVVAGVANA |  |
| 11 | AVAFIEKL | AIAPNTNNL | AIAEFARSLL | ANVGWNNSTFA | CVAMALFAPLVH |  |
| 12 | AVAPIERV | AIAPNTNNL | AIFDRVLTEL | ANVGWNNSTFA | DALDVANKIGII |  |
| 13 | AVFPFQQV | AIAPNTNNL | AIFDRVLTEL | APPGKPQGPPQ | DALDVANKIGII |  |
| 14 | AVIKFLEL | AIARLKEVL | AIFDRVLTEL | ASEAIKGAVVG | DYDFQRDYYDRM |  |
| 15 | AVNKIKEI | AIFQIIQSI | AImGSGILGL | ASEAIKGAVVG | EIKILNIFGVIK |  |
| 16 | AVNKMDQV | AIHHFIEGV | AIVGINLTEM | ASLDKFLASVS | FADKVPKTAENF |  |
| 17 | AVNYFSKV | AIHNFVEVL | AIVGINLTEM | ATAKITAQEAV | FETTDESLRSHF |  |
| 18 | AVTFIERL | AIHNFVEVL | AIVGINLTEM | ATAKITAQEAV | FKNMTPSPFLRF |  |
| 19 | DDLGKDEL | AIIALFHLL | AKDFLAGGVA | AVANEEGFVRL | FLVGGASLKPEF |  |
| 20 | DKFLASVS | AIIALFHLL | ALFPKLWTEL | AVANEEGFVRL | FPSEHFGKLFGL |  |
| 21 | DKFLASVS | AIIALFHLL | ALFPKLWTEL | AVANEEGFVRL | FVFGDENGTVSL |  |
| 22 | ESVVKSED | AIIEYMPLL | ALGQDKVALQ | AVANEEGFVRL | GAVHDVKDVLDS |  |
| 23 | FENYIEEL | AIITGFRNV | ALLATVLGRF | AVAVAAAVGVL | GAVHDVKDVLDS |  |
| 24 | FIFSMVGL | AIKKEMQEL | AQATGKPPQY | AVIDINAFPGY | GEVQIPTDEQTR |  |
| 25 | FIGFFVKL | AIKSPTNTV | ASLGKNPTDE | AVIDINAFPGY | GGGGSYNDFGNY |  |
| 26 | FIGFFVKL | AIMKIISAL | AVAKQGFDFL | AVIDINAFPGY | GGGGSYNDFGNY |  |
| 27 | FIHIITGV | AINAFGESL | AVFDKTLAEL | AVKKWQFVEKL | GGGYDNYGGGNY |  |
| 28 | FIHIITGV | AINELNIKL | AVFGGSFSGY | AVKKWQFVEKL | GSGSGWSSSRGP |  |
| 29 | FIHIITGV | AINKFAETM | AVFGGSFSGY | CVMLILAAPMM | GSVGRMGMGNNY |  |
| 30 | FINFIDKL | AINKFAETM | AVNMVPFPRL | DAVEDLESVGK | GSVGRMGMGNNY |  |
| 31 | FINFIDKL | AINKFAETM | AVRLLLPGEL | DEVGGEALGRL | HVATLSGHSQEV |  |
| 32 | FINRVNEV | AINPKLLQL | AWVNRSSMTF | DEVGGEALGRL | IALYMASQPKNK |  |
| 33 | FINRVNEV | AINSMLDQI | CILAGILVVV | DKFLASVSTVL | IEKLLNYAPLEK |  |
| 34 | FIQDFQKL | AIQEQITRV | CQVYDMRPET | DSYVGDEAQSK | IEKLLNYAPLEK |  |
| 35 | FITPIVKV | AISKIFVLI | CVLISSVAIL | DVVEGEKGAEA | IIFLSDQTKEKE |  |
| 36 | FKTPRGPV | AISRYWYYL | DAYNARMDTS | EETLLQPPLTR | IIFLSDQTKEKE |  |
| 37 | FLFLLFFW | AISSFFRNV | DAYNARMDTS | EIIGKRGIIGY | IIRSMPEQTGEK |  |
| 38 | FLYIFVLE | AITGNFKSL | DIAVNWAGGL | FADKVPKTAEN | IIRSMPEQTGEK |  |
| 39 | FNAGDLLQ | AIVDLIHDI | DMSPLRPQNY | FAKPRNQGGYG | IKSTMGKPQRLY |  |
| 40 | FQMRAGLL | AIVKEIVNI | DSSEEKFLRR | FAVAIMMIGSL | ITAPTLALTIAL |  |
| 41 | FRLLGNVL | AIVRIAEAL | DVKFGADARA | FFAHPDLFVSI | IVVLRNPLIAGK |  |
| 42 | FRLLGNVL | AIYDLIERI | DVKFGADARA | FFAHPDLFVSI | IVVLRNPLIAGK |  |
| 43 | FRLLGNVL | ALAEKFKEL | DYTQQATQSY | FFAHPDLFVSI | KDEAKIAKPVSL |  |
| 44 | FRVPTANV | ALAEKFKEL | EEVQSLPLPL | FIGAGAATVGV | KGYSPTHREEEY |  |
| 45 | FVAKFMAL | ALAKLVEAI | EEVQSLPLPL | FIGAGAATVGV | KKAEAGAGSATE |  |
| 46 | FVAKFMAL | ALAKLVEAI | EFYEKLLSGN | FIGAGAATVGV | KPFPWGDGNHTL |  |
| 47 | FVNGVEKL | ALAKLVEAI | EFYEKLLSGN | FIIGPGGVVNL | KPFPWGDGNHTL |  |
| 48 | FVNGVEKL | ALALRFLAL | EIAGPGFINV | FIIGPGGVVNL | KPFPWGDGNHTL |  |
| 49 | FVNKFNVL | ALDNFLDKL | EIYQKPFQTL | FIIGPGGVVNL | KPFPWGDGNHTL |  |
| 50 | FVNKFNVL | ALERMFLSF | EVAAGGWHSV | FIIGPGGVVNL | KVVLTQANKLGV |  |
| 51 | FVNVQKEL | ALFAGFRAL | FEENVLPPKK | FIIYPGAFVDL | LGLAVTAMKSRP |  |
| 52 | FVNVQKEL | ALFAGFRAL | FETTEESLRN | FIVPLTDLRIP | LGLAVTAMKSRP |  |
| 53 | FVNVQKEL | ALFAGFRAL | FGYSNRVVDL | FLADPDTVNHL | LHLGYLPNQLFR |  |
| 54 | FVQMMTAK | ALFAGFRAL | FIADLDKTLA | FLADPDTVNHL | LLADPQDVGLLL |  |
| 55 | FVQMMTAK | ALFGPWLGL | FIAEVPIIAI | FLADPDTVNHL | LQGVDLLADAVA |  |
| 56 | FVRALAAF | ALFPGVALL | FIFSEKPVFV | FLADPSAFVAA | MATSGQQNNIGM |  |
| 57 | GAEALERM | ALNEKLVNL | FIFSEKPVFV | FLADPSAFVAA | PDGSTSQEESPS |  |
| 58 | GINNIREL | ALNELLQHV | FIFSPYNEVL | FLADPSAFVAA | PDPAKSAPAPKK |  |
| 59 | GLATFHAL | ALNEQIARL | FIFSPYNEVL | FLADPSAFVAA | PEPAKSAPAPKK |  |
| 60 | GLQHLYAL | ALNEQIARL | FINLDGISLL | FLAEASVMTQL | PILPAKKQGGRP |  |
| 61 | GMASYQTQ | ALNEQIARL | FINLDGISLL | FLASESLIKQI | RADDADEFGYSR |  |
| 62 | GVQGFQDY | ALNEQIARL | FIQDFQSVTL | FLASESLIKQI | RADDADEFGYSR |  |
| 63 | IIALFHLL | ALNNLLHSL | FIQDFQSVTL | FLASESLIKQI | RSHFEQWGTLTD |  |
| 64 | IIALFHLL | APEEHPVLL | FISEATVEKL | FLASESLIKQI | SPPGKPQGPPQQ |  |
| 65 | IIALFHLL | APEEHPVLL | FISEATVEKL | FLIGQGAHVGA | SSSGGGGGGGRF |  |
| 66 | IIGTFERM | APEEHPVLL | FISEATVEKL | FLIGQGAHVGA | STYSQAAAQQGY |  |
| 67 | IINFFERL | AQIVQKGGQ | FISGAGILRL | FLIGQGAHVGA | STYSQAAAQQGY |  |
| 68 | IINFFERL | AREKIQDLL | FISGAGILRL | FLPDPSALQNL | VEFSSGLKGMSL |  |
| 69 | IINLFHLI | AVAAVLTQV | FISGAGILRL | FVADEELVHLL | VHLTPEEKSAVT |  |
| 70 | IINQISKL | AVAAVLTQV | FISGAGILRL | FVADEELVHLL | VLSPADKTNVKA |  |
| 71 | IINQISKL | AVADKHELL | FIYTGKAPNL | FVADGIFKAEL | VQTKGTGASGSF |  |
| 72 | IIQSIRMG | AVADKHELL | FIYTGKAPNL | FVADGIFKAEL | VSEKGTVQQADE |  |
| 73 | IKFDTGNL | AVADKHELL | FIYTGKAPNL | FVADGIFKAEL | VSEKGTVQQADE |  |
| 74 | IKFDTGNL | AVADKHELL | FIYTSSVNRL | FVAVPVAVTFL | VSEKGTVQQADE |  |
| 75 | IPNIFQKI | AVAGILKQL | FIYTSSVNRL | FVKALDDLHKM | YVIDLEKGSQHL |  |
| 76 | IPNIFQKI | AVAHAKEYI | FIYTSSVNRL | GAYPTQPGQGY |  |  |
| 77 | IQSIRMGM | AVAKHFVAL | FKQMEQVAQF | GAYPTQPGQGY |  |  |
| 78 | IVAKQVLL | AVAKHFVAL | FLAEEGFYKF | GFTLGNVVGMY |  |  |
| 79 | IVFGEVDR | AVAKHFVAL | FLAEEGFYKF | GGYENQKQSSY |  |  |
| 80 | IVFGEVDR | AVANIVNSV | FLAEEGFYKF | GIALNDHFVKL |  |  |
| 81 | IVNAIMEL | AVANIVNSV | FLAEEGFYKF | GIALNDHFVKL |  |  |
| 82 | IVNAIMEL | AVANIVNSV | FLFGFSAVSI | GINSIGGGQKV |  |  |
| 83 | IVNAIMEL | AVANLFRQL | FLFGFSAVSI | GRDGMDNQGGY |  |  |
| 84 | IVWEFEQL | AVANLFRQL | FLIIAVSLAV | GRDGMDNQGGY |  |  |
| 85 | KIADMGHL | AVAPFFKSY | FLIIAVSLAV | GSGNYNDFGNY |  |  |
| 86 | KIAFFSEL | AVAPFFKSY | FLVGVVVTVL | GSGNYNDFGNY |  |  |
| 87 | KINEIRQM | AVASEFGRL | FPAQVGLLGI | GSHSMRYFSTS |  |  |
| 88 | KINEIRQM | AVASFKHAL | FRVPTANVSV | GVNNISGIEEV |  |  |
| 89 | KINSLAHL | AVASFKHAL | FSQFGPIERA | GVNNISGIEEV |  |  |
| 90 | KITDFIKF | AVASFKHAL | FSVFGQVERA | GVNNISGIEEV |  |  |
| 91 | KLAAFYRL | AVASFVTQL | FSVFGQVERA | GVSDADIQELF |  |  |
| 92 | KVAAIEAL | AVASFVTQL | FSVITVIGAL | HIADVSHFIRP |  |  |
| 93 | KVANVSLL | AVASFVTQL | FVAPIYGADI | HIADVSHFIRP |  |  |
| 94 | KVFKFIGL | AVASFVTQL | FVNDIFERIA | HIADVSHFIRP |  |  |
| 95 | KVGKFYEL | AVAVLRDLL | FVNDIFERIA | HIATVPSLTHL |  |  |
| 96 | KVNPIQGL | AVAVLRDLL | FVNDIFERIA | HIATVPSLTHL |  |  |
| 97 | KVNPIQGL | AVAVLRDLL | FVNDIFERIA | HIATVPSLTHL |  |  |
| 98 | LATVLGRF | AVAVLRDLL | GEVLAVARGL | HVAPSEAANSL |  |  |
| 99 | LATVLGRF | AVFGDFEQF | GGKDVTLLEL | HVAPSEAANSL |  |  |
| 100 | LENYFEEL | AVGEGFHNY | GGKDVTLLEL | HVAPSEAANSL |  |  |
| 101 | LERMFLSF | AVGEGFHNY | GGKDVTLLEL | HVGSGDLFSGM |  |  |
| 102 | LERmFLSF | AVGPVHNSV | GGKDVTLLEL | HVGSGDLFSGM |  |  |
| 103 | LFLFFLFW | AVIGDVIRV | GKVGAHAGEY | HVGSGDLFSGM |  |  |
| 104 | LFQFYKEI | AVIKFLELL | GKVGAHAGEY | HVGSGDLFSGM |  |  |
| 105 | LFQFYKEI | AVIKFLELL | GKVKVGVNGF | HVLESGGFRSL |  |  |
| 106 | LFQFYKEI | AVIKFLELL | GKVKVGVNGF | HVLESGGFRSL |  |  |
| 107 | LIFKLEEL | AVIKFLELL | GMGTLLISKI | HVLESGGFRSL |  |  |
| 108 | LINIFDKL | AVIPRLVQL | GQASQSYSGY | IAKNAGVEGSL |  |  |
| 109 | LINIFDKL | AVMKQFAFV | GQASQSYSGY | IFLGNVPVGGI |  |  |
| 110 | LIRFIEEL | AVMKQFAFV | GVADFSATYL | IFLGNVPVGGI |  |  |
| 111 | LLAHFLLL | AVMKQFAFV | GVADFSATYL | IFLGNVPVGGI |  |  |
| 112 | LLLLRLEP | AVNIIRTFL | GVADFSATYL | IIDDKGILRQI |  |  |
| 113 | LLMTHKVK | AVNIIRTFL | GVSLPDFELL | IIDDKGILRQI |  |  |
| 114 | LMEHIHKL | AVNIIRTFL | HDGHDDDVID | IIGGGMAFTFL |  |  |
| 115 | LMEHIHKL | AVNKMISKV | HINEGETAML | IIGGGMAFTFL |  |  |
| 116 | LMKQVTQL | AVNKMISKV | HINEGETAML | IIGGGMAFTFL |  |  |
| 117 | LSAARYAL | AVNKMISKV | HINEKNFVGL | IIHDPGRGAPL |  |  |
| 118 | LSEKLERI | AVNKMLEYL | HINEKNFVGL | IINAIDGIGGL |  |  |
| 119 | LSKEIELR | AVNPKFLAV | HINEKNFVGL | IINAIDGIGGL |  |  |
| 120 | LVAIFTHL | AVNPKFVAL | HIPERWKDYL | IINAIDGIGGL |  |  |
| 121 | LVAIFTHL | AVNPKFVAL | HIYHQHFDSV | IISGLTGFIGL |  |  |
| 122 | LVAIFTHL | AVNPKFVAL | HIYHQHFDSV | IISGLTGFIGL |  |  |
| 123 | LVNILSHI | AVNQLKDLL | HVATMVDNEL | IISGLTGFIGL |  |  |
| 124 | MAHMASKE | AVQALWKQF | HVFAEKITSL | IISGLTGFIGL |  |  |
| 125 | MAHMASKE | AVSDELIKL | HVFDHPWETV | ILAPAGSLPKI |  |  |
| 126 | MAHMASKE | AVSDILEKL | HVFDHPWETV | ILAQITGTEHL |  |  |
| 127 | MIAHITGL | AVSDILEKL | HVFDHPWETV | ILAQITGTEHL |  |  |
| 128 | MIAHITGL | AVSSWFRQL | IAYARIEGDM | ILASQHAWAQL |  |  |
| 129 | mIAHITGL | AVSSWFRQL | IIAALATLHL | ILNEFGEGSAL |  |  |
| 130 | MINFIHKL | AVTAMKSRP | IIFIGTGEHI | ILNEFGEGSAL |  |  |
| 131 | MKTILSNQ | AVTEAFVRL | IIFMDEIDSI | IPFPGSKLIEV |  |  |
| 132 | MLAPPREL | AVVTQVNKV | IIFMDEIDSI | IPFPGSKLIEV |  |  |
| 133 | MLAPPREL | CFSTAQHAS | IIHDGFGFML | ISAGFNLSSSL |  |  |
| 134 | MLEALERV | CLLQIALLL | IIHDGFGFML | IVADHVASYGV |  |  |
| 135 | MLEALERV | DFHLFKEGI | IIHDGFGFML | IVADHVASYGV |  |  |
| 136 | MLSPRNQL | DFNIIKILN | IIHDGFGFML | IVADHVASYGV |  |  |
| 137 | MQLARELK | DFNIIKILN | IIHNNEGYGV | IVAPGKGILAA |  |  |
| 138 | MREQALLK | DFNIIKILN | IIHNNEGYGV | IVAPGKGILAA |  |  |
| 139 | MRYVASYL | DKFLASVST | IIISEWMGYC | IVHELDQGQQL |  |  |
| 140 | MRYVASYL | DKLHVDPEN | IIMLEALERV | IVHELDQGQQL |  |  |
| 141 | MRYVASYL | DLILPVPAF | IIMLEALERV | IVISAAGIPNL |  |  |
| 142 | NALAHKYH | DPKGPPAKI | IIMLEALERV | IVISAAGIPNL |  |  |
| 143 | NIIKIVGL | DSSEEKFLR | IIMLEALERV | IVISAAGIPNL |  |  |
| 144 | NLAKFNQV | DVANKIGII | IINGGATSHL | IVKDWNDMERI |  |  |
| 145 | NLGKFLEL | DVANKIGII | IINGGATSHL | IVKDWNDMERI |  |  |
| 146 | PGHLQEGF | DVMKDFEEM | IINGGATSHL | IVNILGQEQFM |  |  |
| 147 | PGHLQEGF | EDRLLATLL | IIPNFSLDKI | IVNILGQEQFM |  |  |
| 148 | RIMKAQAL | EDTALLRLL | IIPNFSLDKI | IVNILGQEQFM |  |  |
| 149 | RmKYYELI | EIAKMAEML | IIPNFSLDKI | KEGKEEKEEGE |  |  |
| 150 | RVAGIHKK | EIAKMAEML | IISDGLKYSL | KEGKEEKEEGE |  |  |
| 151 | SLARFSAL | EIFPLFRAL | IISDGLKYSL | KGARADDRRPL |  |  |
| 152 | SLARFSAL | EIMEIMQNL | IIVNWVNETL | KIADFGLSNMM |  |  |
| 153 | SPLRPQNY | EINKELNQV | IIVNWVNETL | KIAVDELFTSL |  |  |
| 154 | SPLRPQNY | EINKELNQV | IIYPGAFVDL | KIAVDELFTSL |  |  |
| 155 | STVLTSKY | EINKKIYKI | IIYPGAFVDL | KIAVDELFTSL |  |  |
| 156 | SVATITRL | EINNLREKV | IKLACADTTF | KIFEMGPVFTL |  |  |
| 157 | SVNDIQRL | EISNLQSKI | ILNSFEHEFL | KIFEMGPVFTL |  |  |
| 158 | TAFTNGYH | ELFTGFIKV | ILNSFEHEFL | KIFEMGPVFTL |  |  |
| 159 | TAQPTQGY | ELFTGFIKV | ILNSFEHEFL | KIFEMGPVFTL |  |  |
| 160 | TISRFEAL | ELLFQREIL | ILNSLYNENL | KIIDEDGLLNL |  |  |
| 161 | TQQATQSY | EPNPDDPLM | ILNSLYNENL | KIIDEDGLLNL |  |  |
| 162 | TQQATQSY | EPNPDDPLM | ILNSLYNENL | KIIDEDGLLNL |  |  |
| 163 | TVLTSKYR | ERGILDLNL | ILPSAIEVQL | KIIDEDGLLNL |  |  |
| 164 | TVLTSKYR | EVAKEWQEL | IMGGTLLALL | KIKDLSTVEAL |  |  |
| 165 | TVLTSKYR | EVAKEWQEL | IMGGTLLALL | KIKDLSTVEAL |  |  |
| 166 | VANKIGII | EVIGKITAL | IMGGTLLALL | KIKDLSTVEAL |  |  |
| 167 | VANKIGII | EVIGKITAL | INDPFIDLNY | KIPVTIITGYL |  |  |
| 168 | VDIINAKQ | EVIGKITAL | IPFDVKWQSL | KLADFGVAGQL |  |  |
| 169 | VDIINAKQ | EVIGKITAL | IPYTGPFNLL | KLADFGVAGQL |  |  |
| 170 | VDIINAKQ | EVISKLYAV | IPYTGPFNLL | KLIDFGSGALL |  |  |
| 171 | VEEVDAAM | EVNAAWERL | IPYTGPFNLL | KPFDKFQFERL |  |  |
| 172 | VGKDRSSF | EVNKMESQL | IPYTGPFNLL | KPFDKFQFERL |  |  |
| 173 | VHLTPEEK | EVNKMESQL | IQIPPGAHAP | KSADEVLFTGV |  |  |
| 174 | VIAGFNRL | EVNKVISSV | IQVPIPDVYQ | KVAPADGFSDL |  |  |
| 175 | VIGKITAI | EVQSLPLPL | ISLKQAPLVH | KVAPADGFSDL |  |  |
| 176 | VIGKITAI | FAFLISERL | ISLKQAPLVH | KVAPADGFSDL |  |  |
| 177 | VIKFLELL | FAFLISERL | IVADLSEQSL | KVNPIQGLASK |  |  |
| 178 | VINIIHSL | FAFLISERL | IVADLSEQSL | KVSDIPQYSQL |  |  |
| 179 | VINSFVHV | FAFLISERL | IVADLSEQSL | KVSDIPQYSQL |  |  |
| 180 | VINSFVHV | FAFRNFLYL | IVAKNGFSGL | KVSDIPQYSQL |  |  |
| 181 | VITKFINV | FAIPLIEKL | IVFNGPHLLL | LAHLDNLKGTF |  |  |
| 182 | VITKFINV | FAIPLIEKL | IVFNGPHLLL | LAVASFPKKQE |  |  |
| 183 | VLATMERL | FAIPLIEKL | IVFTGEKEQL | LAVASFPKKQE |  |  |
| 184 | VLGEFERL | FAIPLIEKL | IVISVGIYNL | LEEVQSLPLPL |  |  |
| 185 | VLLESEQF | FAISILQQI | IVISVGIYNL | LEEVQSLPLPL |  |  |
| 186 | VLLESEQF | FAMPYFIQV | IVISVGIYNL | LIVDSRELGYL |  |  |
| 187 | VNLPKADI | FASGLIHRV | IVISVGIYNL | LIVDSRELGYL |  |  |
| 188 | VPNGFSKL | FAYDGKDYI | IVNKDAKSTL | LIVDSRELGYL |  |  |
| 189 | VSIAPRSI | FAYDGKDYI | IVNKDAKSTL | LLEPYKPPSAQ |  |  |
| 190 | VTKYTSSK | FAYDGKDYI | IVNVGSIVGL | LLRSAELALLL |  |  |
| 191 | VVAEFGRI | FDRSLFVVL | IVNVGSIVGL | LLRSAELALLL |  |  |
| 192 | WLQKIEAI | FFMPGFAPL | IVNVGSIVGL | LLRSAELALLL |  |  |
| 193 | WVEKFINV | FIADSHQNF | IVTPFNIDRL | LRVAPEEHPTL |  |  |
| 194 | WVEKFINV | FIADSHQNF | IVTPFNIDRL | LRVAPEEHPTL |  |  |
| 195 | YANMFERL | FIADSHQNF | IVTPFNIDRL | LRVAPEEHPTL |  |  |
| 196 | YDYYRDDF | FIAHFQDNL | IVTPFNIDRL | LRVAPEEHPVL |  |  |
| 197 | YEVSQLKD | FIAHFQDNL | KEQISDIDDA | LRVAPEEHPVL |  |  |
| 198 | YGVIKTDM | FIAHFQDNL | KIADFGFSNL | LRVAPEEHPVL |  |  |
| 199 | YIAQFFKI | FIAHFQDNL | KIADFGFSNL | LTMSEKGFQQI |  |  |
| 200 | YIFSFDKM | FIAIKPDGV | KIADFGFSNL | LTMSEKGFQQI |  |  |
| 201 | YIKRLEEL | FIAIKPDGV | KIADFGFSNL | LTMSEKGFQQI |  |  |
| 202 | YINEFVSI | FIAIKPDGV | KIADFGLARI | MDALCGSGELG |  |  |
| 203 | YINKVEEL | FIANLFNRY | KIADFGLSNM | MGGNPLENSGF |  |  |
| 204 | YITWIDKY | FIANLFNRY | KIADFGLSNM | MGGNPLENSGF |  |  |
| 205 | YKFQNALL | FIANLFNRY | KIADFGLSNM | MIGDILLFGTL |  |  |
| 206 | YLARFDAL | FIAPSGHGL | KIAEFSFDLL | MIGDILLFGTL |  |  |
| 207 | YLNSVQRL | FIAPSGHGL | KIAEGVNSLL | MPVGPDAILRY |  |  |
| 208 | YLNSVQRL | FIAPSGHGL | KIAEGVNSLL | MPVGPDAILRY |  |  |
| 209 | YNARMDTS | FIAPTGHSL | KIAEGVNSLL | MPVGPDAILRY |  |  |
| 210 | YPKIIQDI | FIAPTGHSL | KIAEGVNSLL | MPVGPDAILRY |  |  |
| 211 | YVFQMEMI | FIAPTGHSL | KIFEGGKLQF | MRKAGIFQSVK |  |  |
| 212 | YVIPKTGF | FIASVFMLH | KIFQGNVHNF | NEDVSQEESPS |  |  |
| 213 | YVIYIERV | FIEENDEVL | KIFSGVFVKV | NIASATKFKQL |  |  |
| 214 | YVIYIERV | FIENQGLEL | KIFSGVFVKV | NIASATKFKQL |  |  |
| 215 | YVIYIERV | FIFDVGTRL | KIFSGVFVKV | NINIEKAFLTL |  |  |
| 216 | YVNNMARV | FIFDVGTRL | KIFSGVFVKV | NINIEKAFLTL |  |  |
| 217 | YVSAFSKL | FIFEKILQL | KIFSQEYINL | NISPADAHRNL |  |  |
| 218 | YVSAFSKL | FIFSDTHEL | KIFTDIFHYL | NISPADAHRNL |  |  |
| 219 | YVSAFSKL | FIFSDTHEL | KIFTDIFHYL | NISPADAHRNL |  |  |
| 220 | YVTTSTRT | FIFSDTHEL | KIIGINGDFF | NLRTALIFGGF |  |  |
| 221 | YWRQAGLS | FIFSDTHEL | KIPNFDVREI | NQQPSNYGPMK |  |  |
| 222 |  | FIHQHFVEV | KIPNFDVREI | NQQPSNYGPMK |  |  |
| 223 |  | FIHQHFVEV | KIPNFDVREI | NVAPLAVFQML |  |  |
| 224 |  | FIINGIEKV | KIYEEAWTKY | NVAPLAVFQML |  |  |
| 225 |  | FIINGIEKV | KIYPQIPDHL | NVNAAMVFEFL |  |  |
| 226 |  | FIINGIEKV | KLFPDTPLAL | PDHMLAMVEEA |  |  |
| 227 |  | FIINGIEKV | KLFPDTPLAL | PDPAKSAPAPK |  |  |
| 228 |  | FIISDWRFV | KLFPDTPLAL | PEPAKSAPAPK |  |  |
| 229 |  | FIKAIGVGL | KLKSFESERV | PPVPGQALLDL |  |  |
| 230 |  | FIKAIGVGL | KLKSFESERV | PRIASNAGSIA |  |  |
| 231 |  | FIKAIGVGL | KLKSFESERV | PRKIEEIKDFL |  |  |
| 232 |  | FIKDNWEEL | KSIDDIGGAL | PVVPVHLDTTI |  |  |
| 233 |  | FIKDNWEEL | KVADFGLSRL | QIAWVGGFIAI |  |  |
| 234 |  | FIKFDTGNL | KVADWTGATY | QIAWVGGFIAI |  |  |
| 235 |  | FIKFDTGNL | KVFPEDMAKY | QIAWVGGFIAI |  |  |
| 236 |  | FIKPNFQQL | KVIEEEWQQV | QIIRDEGISAL |  |  |
| 237 |  | FIKSFLVFI | KVIEEEWQQV | QIIRDEGISAL |  |  |
| 238 |  | FILANEHNV | KVIEEEWQQV | QIIRDEGISAL |  |  |
| 239 |  | FILANEHNV | KVNEDMNAKL | QIIRDEGISAL |  |  |
| 240 |  | FINELITEL | KVNEDMNAKL | QTAPEEAFIKL |  |  |
| 241 |  | FINIVVHSV | KVNEDMNAKL | QTAPEEAFIKL |  |  |
| 242 |  | FINKFVQFI | KVVEDGILKL | QTAPEEAFIKL |  |  |
| 243 |  | FINNINSVL | KVYQEIWIGM | QVADGSLINQL |  |  |
| 244 |  | FINNINSVL | KVYQEIWIGM | QVAGPDRFVVL |  |  |
| 245 |  | FINQDNNTL | KVYQEIWIGM | QVAGPDRFVVL |  |  |
| 246 |  | FINQDNNTL | KVYQEIWIGM | QVAGPDRFVVL |  |  |
| 247 |  | FINRMEESL | LFRTVLDSGI | QVIEAEGLKEM |  |  |
| 248 |  | FINRMEESL | LHLGYLPNQL | QVIEAEGLKEM |  |  |
| 249 |  | FINRMEESL | LHLGYLPNQL | QVIEAEGLKEM |  |  |
| 250 |  | FINTVITSL | LIAKMEEQGL | QVISVSGVIGL |  |  |
| 251 |  | FIQEEFQHL | LIAKMEEQGL | QVNGGTVAEKL |  |  |
| 252 |  | FIQEEFQHL | LIAKMEEQGL | QVNGGTVAEKL |  |  |
| 253 |  | FIQEEFQHL | LIFLDDPQAV | QVTDSAAFNAL |  |  |
| 254 |  | FIQEIEHAL | LIFLDDPQAV | QVTDSAAFNAL |  |  |
| 255 |  | FIREQLELL | LIFLDDPQAV | RIAAQGFTVAA |  |  |
| 256 |  | FIREQLELL | LIFLDDPQAV | RIIDKNGIHDL |  |  |
| 257 |  | FIREQLELL | LIKDFLDEHY | RIIDKNGIHDL |  |  |
| 258 |  | FIRVIGSEL | LIKDFLDEHY | RIIDKNGIHDL |  |  |
| 259 |  | FISEFEHRV | LIKDFLDEHY | RIIDKNGIHDL |  |  |
| 260 |  | FISEFEHRV | LIMLDNQKLV | RIIRYPDSHQL |  |  |
| 261 |  | FISEIIHQL | LINELIREYL | RIITAEGIILL |  |  |
| 262 |  | FISGLFNFY | LINLIPLHVL | RIITAEGIILL |  |  |
| 263 |  | FISGLFNFY | LINLIPLHVL | RIITAEGIILL |  |  |
| 264 |  | FISGLFNFY | LINLIPLHVL | RIVAEEFLKNF |  |  |
| 265 |  | FISNVKTAL | LKSGADLSLQ | RLAAGSLFAGF |  |  |
| 266 |  | FISNVKTAL | LLENQNTQEI | RLAAGSLFAGF |  |  |
| 267 |  | FISPFGHGL | LLQGTLAQSI | RLAAGSLFAGF |  |  |
| 268 |  | FISPFGHGL | LLYQGPHNTL | RLAAGSLFAGF |  |  |
| 269 |  | FISPFGHGL | LLYQGPHNTL | RMAEDELFNRY |  |  |
| 270 |  | FISPFGHGL | LLYQGPHNTL | RVAAQGFVVGA |  |  |
| 271 |  | FISPWWDEV | LNVQNKNSSY | RVAEITKEQRL |  |  |
| 272 |  | FISVIKEML | LPSPVADHVK | RVFSGLVSTGL |  |  |
| 273 |  | FISVIKEML | LQSCIKLIKL | RVNLFTDFDKY |  |  |
| 274 |  | FISVIKEML | LQSCIKLIKL | SAYTAQPTQGY |  |  |
| 275 |  | FISVIKEML | LSAAADSIKI | SAYTAQPTQGY |  |  |
| 276 |  | FITSFPEGY | LSIRGNNIRY | SDVLELTDDNF |  |  |
| 277 |  | FITSFPEGY | LTIEQGNLGL | SHGSQETDEEF |  |  |
| 278 |  | FITVVPTKL | LVGLFEDTNL | SINPLGGFVHY |  |  |
| 279 |  | FITVVPTKL | LVGLFEDTNL | SIYPHGSTDKL |  |  |
| 280 |  | FITVVPTKL | LYQALASGKI | SLLSRSGLSGF |  |  |
| 281 |  | FIVIVPQKL | MAPARLFALL | SPPGKPQGPPP |  |  |
| 282 |  | FIVIVPQKL | MDTISNKKVL | SSAPAQGFVGV |  |  |
| 283 |  | FIVIVPQKL | MIHLGHILFL | SSSKGSLGGGF |  |  |
| 284 |  | FIYGNQDLF | MIHLGHILFL | SSYGQSQNTGY |  |  |
| 285 |  | FIYGNQDLF | MINKNMTHQV | STYGNPGSQGY |  |  |
| 286 |  | FKDPLLMKM | MLFEEAFVHL | SVANHNSFLNL |  |  |
| 287 |  | FLADIVQKL | mLmTDAGRKL | SVANHNSFLNL |  |  |
| 288 |  | FLADIVQKL | MLNKGFKEQI | SVANHNSFLNL |  |  |
| 289 |  | FLAKDFNFL | MLNKGFKEQI | SVASISHQEQL |  |  |
| 290 |  | FLAKDFNFL | MSIFTFMGAN | SVASISHQEQL |  |  |
| 291 |  | FLAKDFNFL | MVARLGLGLL | SVASISHQEQL |  |  |
| 292 |  | FLDDLFKAI | NSMGKSLPLF | SVNNFPTAAGL |  |  |
| 293 |  | FLDDLFKAI | NVEWAKPSTN | SVNNFPTAAGL |  |  |
| 294 |  | FLFDTQHFI | NVEWAKPSTN | SVYDGHGGEEV |  |  |
| 295 |  | FLFKWWDNL | PTISSYENLL | TFDVAPSRLDF |  |  |
| 296 |  | FLFKWWDNL | PTTKTYFPHF | TFDVAPSRLDF |  |  |
| 297 |  | FLFKWWDNL | PTTKTYFPHF | TIAREEGFRGL |  |  |
| 298 |  | FLFSKFIEL | PVQAPQWTDF | TIAREEGFRGL |  |  |
| 299 |  | FLFSKFIEL | QIFLRDIEQV | TIAREEGFRGL |  |  |
| 300 |  | FLITILDHL | QIFLRDIEQV | TIGTIDEIQKL |  |  |
| 301 |  | FLITILDHL | QIFLRDIEQV | TIGTIDEIQKL |  |  |
| 302 |  | FLITILDHL | QIFNGTFVKL | TIGTIDEIQKL |  |  |
| 303 |  | FLKDKFVEI | QIFNGTFVKL | TIIDLPGITRV |  |  |
| 304 |  | FLKDKFVEI | QIFNGTFVKL | TIIDLPGITRV |  |  |
| 305 |  | FLKDKFVEI | QIFNGTFVKL | TIIDLPGITRV |  |  |
| 306 |  | FLKEIVETF | QIIQSIRMGM | TIIDTKGVTAL |  |  |
| 307 |  | FLKEIVETF | QIKSFEKSQK | TIIDTKGVTAL |  |  |
| 308 |  | FLKEIVETF | QIKSFEKSQK | TIIDTKGVTAL |  |  |
| 309 |  | FLKEIVETF | QILLKALTNL | TIIDTKGVTAL |  |  |
| 310 |  | FLLEREQLL | QILLKALTNL | TLHPISSEELL |  |  |
| 311 |  | FLMDFIHQV | QILLKALTNL | TLHPISSEELL |  |  |
| 312 |  | FLMDFIHQV | QINQNGEVRL | TLHPISSEELL |  |  |
| 313 |  | FLMDFIHQV | QITRELIKYQ | TNGIYPHKLVF |  |  |
| 314 |  | FLNDIFERI | QIYDKFIAQL | TNGIYPHKLVF |  |  |
| 315 |  | FLNKTTHQL | QIYDKFIAQL | TVAAASTFNGF |  |  |
| 316 |  | FLREYFERL | QIYDKFIAQL | TVAAASTFNGF |  |  |
| 317 |  | FLREYFERL | QLNLTKIEQL | TVATQTQFFHV |  |  |
| 318 |  | FLSELTQQL | QLNLTKIEQL | VAHVDDMPNAL |  |  |
| 319 |  | FLSSVIQNL | QLNLTKIEQL | VAHVDDMPNAL |  |  |
| 320 |  | FLSSVIQNL | QVAKAVTQAL | VAIDAGHESFL |  |  |
| 321 |  | FLSSVIQNL | QVAKAVTQAL | VELDDLGKDEL |  |  |
| 322 |  | FLSSVIQNL | QVFEGHTHYV | VELDDLGKDEL |  |  |
| 323 |  | FLYPFPLAL | QVFPGLLERV | VELDDLGKDEL |  |  |
| 324 |  | FLYSDEVQI | QVFPGLLERV | VFAGKQGFITL |  |  |
| 325 |  | FPMEIRQYL | QVIGGIWVTY | VFATGAYPRLS |  |  |
| 326 |  | FPQKFIDLL | QVLSGVSQLL | VHLTPEEKSAV |  |  |
| 327 |  | FPQKWPDLL | RIGELQGHYL | VIEMVLATDMS |  |  |
| 328 |  | FPYPYAERL | RIKETDEQRL | VIEMVLATDMS |  |  |
| 329 |  | FQRDYYDRM | RINEIKSEEV | VIFILGKEHQL |  |  |
| 330 |  | FTAEFLEKV | RINEIKSEEV | VIFILGKEHQL |  |  |
| 331 |  | FTAEFLEKV | RIPDYLWMGL | VIGSSTLFSAL |  |  |
| 332 |  | FTNDKIINL | RIPDYLWMGL | VIGSSTLFSAL |  |  |
| 333 |  | FVADFADKL | RIPDYLWMGL | VIGSSTLFSAL |  |  |
| 334 |  | FVADFADKL | RIVDDFGTHL | VIGSSTLFSAL |  |  |
| 335 |  | FVADFADKL | RLALFPGVAL | VIHKGHEKEAL |  |  |
| 336 |  | FVADFADKL | RLFLSLPVLV | VIHKGHEKEAL |  |  |
| 337 |  | FVAETSQRI | RLISGDNAEI | VIHKGHEKEAL |  |  |
| 338 |  | FVAETSQRI | RLKDSWVERL | VINEDGSRTAL |  |  |
| 339 |  | FVAETSQRI | RLKDSWVERL | VINEDGSRTAL |  |  |
| 340 |  | FVAKFMALY | RLKDSWVERL | VINEDGSRTAL |  |  |
| 341 |  | FVAKFMALY | RLPVGGFADL | VINGLLGNIGL |  |  |
| 342 |  | FVAKFMALY | RLTAGTGHKL | VINGLLGNIGL |  |  |
| 343 |  | FVAKTWTEL | RPYDNWLEGV | VINGLLGNIGL |  |  |
| 344 |  | FVAKTWTEL | RVADFGSATF | VINGVGGNLVA |  |  |
| 345 |  | FVANAIIHL | RVADFGSATF | VLFDKATYDKL |  |  |
| 346 |  | FVANAIIHL | RVAELEKQLL | VLFDKATYDKL |  |  |
| 347 |  | FVANAIIHL | RVAELEKQLL | VLGAVMAAMGF |  |  |
| 348 |  | FVANLFNKY | RVAELEKQLL | VNLPINGNGKQ |  |  |
| 349 |  | FVANLFNKY | RVAPEEHPTL | VNLPINGNGKQ |  |  |
| 350 |  | FVATGFHGL | RVAPEEHPVL | VPISVTGIAQV |  |  |
| 351 |  | FVATGFHGL | RVAPEEHPVL | VPISVTGIAQV |  |  |
| 352 |  | FVFKGFDAL | RVAPEEHPVL | VPISVTGIAQV |  |  |
| 353 |  | FVFKGFDAL | RVFSDIIYTV | VPNPKMPNTFM |  |  |
| 354 |  | FVFKGFDAL | RVKEPVKELL | VQVNSIKFDSE |  |  |
| 355 |  | FVFPGELLL | SIAKEGFEKI | VSLGAGAKDEL |  |  |
| 356 |  | FVFPGELLL | SIAKEGFEKI | VTEGQISTEVT |  |  |
| 357 |  | FVFPGELLL | SIAKEGFEKI | VVAGGLGRQLL |  |  |
| 358 |  | FVFPGELLL | SIASAIVNEL | VVAGGLGRQLL |  |  |
| 359 |  | FVGLIPHLL | SIASFSKQEV | VVAGGLGRQLL |  |  |
| 360 |  | FVGQAGQKL | SIASFSKQEV | VVAGGLGRQLL |  |  |
| 361 |  | FVHPGAATV | SIASFSKQEV | VVAPVSQFPFA |  |  |
| 362 |  | FVHQGETEL | SIFEGGATEL | VVATGGKENAL |  |  |
| 363 |  | FVHQGETEL | SIFGSSFSGL | VVATGGKENAL |  |  |
| 364 |  | FVHQGETEL | SIFSEIFQRL | VVIAHDVDPIE |  |  |
| 365 |  | FVIDLQTRL | SIFSEIFQRL | VVISSDGQFAL |  |  |
| 366 |  | FVIRNIVEA | SIFSEIFQRL | VVISSDGQFAL |  |  |
| 367 |  | FVIRNIVEA | SIYNGDMEKI | VVISSDGQFAL |  |  |
| 368 |  | FVIRNIVEA | SIYNGDMEKI | VVISSDGQFAL |  |  |
| 369 |  | FVKAFILGF | SIYNGDMEKI | VVITEKGISDL |  |  |
| 370 |  | FVKAFILGF | SIYNGDMEKI | VVITEKGISDL |  |  |
| 371 |  | FVKAFILGF | SLFAGGMLRV | VVITEKGISDL |  |  |
| 372 |  | FVKDGMDTV | SLFAGGMLRV | VVITEKGISDL |  |  |
| 373 |  | FVKGILDYL | SLFAGGMLRV | VVLRNPLIAGK |  |  |
| 374 |  | FVKGILDYL | SLFAGGMLRV | VVLRNPLIAGK |  |  |
| 375 |  | FVKGILDYL | SLFPHNPQFI | VVNPASAFQGL |  |  |
| 376 |  | FVKPAFEEF | SLFPHNPQFI | VVNPASAFQGL |  |  |
| 377 |  | FVKPAFEEF | SLFPHNPQFI | VVNPASAFQGL |  |  |
| 378 |  | FVMETFVHL | SLFPHNPQFI | VVSSVPLGGGL |  |  |
| 379 |  | FVMETFVHL | SLLEQGLVEA | WANTLGSLALL |  |  |
| 380 |  | FVMETFVHL | SLYASGRTTG | WANTLGSLALL |  |  |
| 381 |  | FVMETFVHL | SPLRPQNYLF | WANTLGSLALL |  |  |
| 382 |  | FVNDIFERI | SSAGLIYLHF | WIITGGSHTGV |  |  |
| 383 |  | FVNDIFERI | SSLNLRETNL | WIITGGSHTGV |  |  |
| 384 |  | FVNDIFERI | SVNKESMIGV | WIVDNEGLTSL |  |  |
| 385 |  | FVNDIFERI | SVNKESMIGV | WIVDNEGLTSL |  |  |
| 386 |  | FVNDVKDYL | SVPTFSWEEI | WLANEGLITRL |  |  |
| 387 |  | FVNDVKDYL | SVPTFSWEEI | WLANEGLITRL |  |  |
| 388 |  | FVNDVKDYL | SVPTFSWEEI | WLANEGLITRL |  |  |
| 389 |  | FVNEIISRI | SVTLDDLADF | WVVRLGAEEAL |  |  |
| 390 |  | FVNEIISRI | SYDPTIENTF | WVVRLGAEEAL |  |  |
| 391 |  | FVNEIISRI | SYDPTIENTF | WVVRLGAEEAL |  |  |
| 392 |  | FVNEIISRI | TAFQGPWARL | YASGRTTGIVM |  |  |
| 393 |  | FVNILTHNL | TGGKILGFFF | YDFQRDYYDRM |  |  |
| 394 |  | FVNILTHNL | TGGKILGFFF | YIADLDAKSGA |  |  |
| 395 |  | FVNILTHNL | TIFKQAFSLL | YIAGHPAFVNY |  |  |
| 396 |  | FVNKAESAL | TIFPWLVESI | YIAGHPAFVNY |  |  |
| 397 |  | FVNKFNVLY | TIFTGSIERM | YIAGHPAFVNY |  |  |
| 398 |  | FVNKFNVLY | TIFTGSIERM | YIAGHPAFVNY |  |  |
| 399 |  | FVNKFNVLY | TIFTGSIERM | YIATITDKELL |  |  |
| 400 |  | FVNQLNTKY | TIFTGSIERM | YIATITDKELL |  |  |
| 401 |  | FVNSSETRL | TINKNARATL | YIATITDKELL |  |  |
| 402 |  | FVNSSETRL | TINKNARATL | YIATITDKEML |  |  |
| 403 |  | FVNYATEKL | TINKNARATL | YIATITDKEML |  |  |
| 404 |  | FVQELNKSL | TINPVPLVGL | YIATITDKEML |  |  |
| 405 |  | FVVDRNTIS | TIYDNAVQGL | YIIGGSVINEL |  |  |
| 406 |  | FWPPYVELL | TPAGVLSYQF | YIIGGSVINEL |  |  |
| 407 |  | GAEALERMF | TVFLSTWNKL | YIIGGSVINEL |  |  |
| 408 |  | GAEALERMF | TVFSKSFEQV | YIIGGSVINEL |  |  |
| 409 |  | GAEALERmF | TVFSKSFEQV | YIPTAAAFGGL |  |  |
| 410 |  | GAYKYIQEL | TVFTDHMLTV | YIPTAAAFGGL |  |  |
| 411 |  | GIAAIREQV | TVFTDHMLTV | YIPTAAAFGGL |  |  |
| 412 |  | GIAAIREQV | TVNEMSRVYL | YISNGELFQVL |  |  |
| 413 |  | GIAAIREQV | TVNEMSRVYL | YISNGELFQVL |  |  |
| 414 |  | GIADLAHLL | TVNSASQLQA | YISNGELFQVL |  |  |
| 415 |  | GIADLAHLL | TVNSASQLQA | YLKQTSRIAIG |  |  |
| 416 |  | GIADLAHLL | VAFTPEGERL | YLVGLFEDTNL |  |  |
| 417 |  | GIAFGFHQL | VAHVDDMPNA | YLVGLFEDTNL |  |  |
| 418 |  | GIAGYLHLL | VANEEGFVRL | YPNNKAAKTQL |  |  |
| 419 |  | GIAHFLEKL | VANSAFVERV | YPNNKAAKTQL |  |  |
| 420 |  | GIAHFLEKL | VAYWRQAGLS | YQKVVAGVANA |  |  |
| 421 |  | GIAHFLEKL | VAYWRQAGLS | YVGDLHPDVTE |  |  |
| 422 |  | GIAHFLEKL | VEFTRSLFVN |  |  |  |
| 423 |  | GIAQMLEKY | VEKVFGVSLV |  |  |  |
| 424 |  | GIFSDIHLL | VEKVFGVSLV |  |  |  |
| 425 |  | GIIDAFHQI | VFDMGAEVGF |  |  |  |
| 426 |  | GIITGIRGL | VFDMGAEVGF |  |  |  |
| 427 |  | GIMDRFLQV | VFDMGAEVGF |  |  |  |
| 428 |  | GIMDRFLQV | VFLNQIYTQL |  |  |  |
| 429 |  | GIMDRFLQV | VIAEAVRTTL |  |  |  |
| 430 |  | GIMDRFLQV | VIAEAVRTTL |  |  |  |
| 431 |  | GINPANIGF | VIAEAVRTTL |  |  |  |
| 432 |  | GINRIGNLL | VIFPTGDSKV |  |  |  |
| 433 |  | GIYTGFIKV | VIFPTGDSKV |  |  |  |
| 434 |  | GIYTGFIKV | VIFPTGDSKV |  |  |  |
| 435 |  | GIYTGFIKV | VIFSGSLDFF |  |  |  |
| 436 |  | GLAGEFQRL | VIFTSPIKAL |  |  |  |
| 437 |  | GLASKFTSL | VIFTSPIKAL |  |  |  |
| 438 |  | GLFQGKTPL | VIFTSPIKAL |  |  |  |
| 439 |  | GQGFRLGDQ | VIFTSPIKAL |  |  |  |
| 440 |  | GQQSSYPGY | VIIEYPTLHV |  |  |  |
| 441 |  | GQQSSYPGY | VIKEDAAEGL |  |  |  |
| 442 |  | GTQSTPQGY | VINDVRDIFL |  |  |  |
| 443 |  | GVAAFHAFL | VINDVRDIFL |  |  |  |
| 444 |  | GVAGKFVEF | VINDVRDIFL |  |  |  |
| 445 |  | GVAREWFFL | VINDVRDIFL |  |  |  |
| 446 |  | GVAREWFFL | VINESGLYRL |  |  |  |
| 447 |  | GVAREWFFL | VINESGLYRL |  |  |  |
| 448 |  | GVARHFNYV | VINKEDFVAL |  |  |  |
| 449 |  | GVARLFTKV | VINKEDFVAL |  |  |  |
| 450 |  | GVASLSERL | VINPAVGSGL |  |  |  |
| 451 |  | GVATDIQAL | VINPAVGSGL |  |  |  |
| 452 |  | GVFKDFDFL | VINPNGKSEV |  |  |  |
| 453 |  | GVFKDFDFL | VINPNGKSEV |  |  |  |
| 454 |  | GVFKDFDFL | VINPNGKSEV |  |  |  |
| 455 |  | GVFKDFDFL | VINQTQKENL |  |  |  |
| 456 |  | GVGPVPARA | VINQTQKENL |  |  |  |
| 457 |  | GVHGNFPRL | VINQTQKENL |  |  |  |
| 458 |  | GVIKVFNDM | VINQTQKENL |  |  |  |
| 459 |  | GVKGSFEEL | VISQAAVVHA |  |  |  |
| 460 |  | GVKGSFEEL | VISQAAVVHA |  |  |  |
| 461 |  | GVKGSFEEL | VISQAAVVHA |  |  |  |
| 462 |  | GVKKDIEKL | VISQAAVVHA |  |  |  |
| 463 |  | GVKKDIEKL | VITKGTVIEV |  |  |  |
| 464 |  | GVKQLIVGV | VITKGTVIEV |  |  |  |
| 465 |  | GVKQLIVGV | VLFTPTVLAK |  |  |  |
| 466 |  | GVKQLIVGV | VLFTPTVLAK |  |  |  |
| 467 |  | GVLPNIQAV | VLFTPTVLAK |  |  |  |
| 468 |  | GVNKIISSL | VLFTPTVLAK |  |  |  |
| 469 |  | GVNKIISSL | VLGPAEAHYA |  |  |  |
| 470 |  | GVNKIISSL | VLGPAEAHYA |  |  |  |
| 471 |  | GVNKIISSL | VLGPAEAHYA |  |  |  |
| 472 |  | GVQQWTERL | VLHKEEKQHL |  |  |  |
| 473 |  | GVSHFFREL | VLLAFSWQNP |  |  |  |
| 474 |  | GVSHFFREL | VLRNPLIAGK |  |  |  |
| 475 |  | GVSKEFFQL | VLRNPLIAGK |  |  |  |
| 476 |  | GVSKEFFQL | VLSGISLAVN |  |  |  |
| 477 |  | HIADVSHFI | VLSGISLAVN |  |  |  |
| 478 |  | HIADVSHFI | VLSGISLAVN |  |  |  |
| 479 |  | HIADVSHFI | VLSGISLAVN |  |  |  |
| 480 |  | HIADVSKKV | VLSPADKTNV |  |  |  |
| 481 |  | HIADVSKKV | VLSPADKTNV |  |  |  |
| 482 |  | HIADVSKKV | VPYEKGFALL |  |  |  |
| 483 |  | HIAGFLDGF | VPYEKGFALL |  |  |  |
| 484 |  | HIAGFLDGF | VVADLFHSLL |  |  |  |
| 485 |  | HIAKTLAQL | VVAEFGRIAV |  |  |  |
| 486 |  | HIAKTLAQL | VVAEFGRIAV |  |  |  |
| 487 |  | HIALIIELL | VVKDLSSEEL |  |  |  |
| 488 |  | HIIENIVAV | VVLRNPLIAG |  |  |  |
| 489 |  | HIIENIVAV | VVNKYIDQGI |  |  |  |
| 490 |  | HIIGRIESY | VVNNNEMVAL |  |  |  |
| 491 |  | HIIGRIESY | VVNNNEMVAL |  |  |  |
| 492 |  | HIIGRIESY | VVNPGANQNL |  |  |  |
| 493 |  | HIIGRIESY | VVNPGANQNL |  |  |  |
| 494 |  | HIKDSFHSL | VVNPGANQNL |  |  |  |
| 495 |  | HIMEIFSTY | VVNSITGERL |  |  |  |
| 496 |  | HINENFAKL | VVNSITGERL |  |  |  |
| 497 |  | HINENFAKL | VVSKFSFVSL |  |  |  |
| 498 |  | HINENFAKL | WIAGDIMHNL |  |  |  |
| 499 |  | HINENFAKL | WIAGDIMHNL |  |  |  |
| 500 |  | HINLFFTEL | WIAGDIMHNL |  |  |  |
| 501 |  | HINLFFTEL | WIHDNGEFYL |  |  |  |
| 502 |  | HINLFFTEL | WIIDKDFGYL |  |  |  |
| 503 |  | HINLFFTEL | WIIDKDFGYL |  |  |  |
| 504 |  | HINPANKSL | WIIDKDFGYL |  |  |  |
| 505 |  | HINPANKSL | WINEKDINAV |  |  |  |
| 506 |  | HINSIKSVF | WINEKDINAV |  |  |  |
| 507 |  | HIQEIMVQL | WINEKDINAV |  |  |  |
| 508 |  | HIRVGWEQL | WINEKDINAV |  |  |  |
| 509 |  | HIRVGWEQL | WIYSVNKEQL |  |  |  |
| 510 |  | HLFDAFVSV | YEKEKLNERL |  |  |  |
| 511 |  | HLIGDFSKV | YIAEMVRHAL |  |  |  |
| 512 |  | HLNQLREQL | YIAEMVRHAL |  |  |  |
| 513 |  | HLNQLREQL | YIAEMVRHAL |  |  |  |
| 514 |  | HLNQLREQL | YIAEMVRHAL |  |  |  |
| 515 |  | HLYSSIEHL | YIAQFTSQFL |  |  |  |
| 516 |  | HPLHNDPNL | YIFEGLGHLI |  |  |  |
| 517 |  | HPTIISESF | YIFEGLGHLI |  |  |  |
| 518 |  | HPTIISESF | YIFEGLGHLI |  |  |  |
| 519 |  | HPTIISESF | YIFEGLGHLI |  |  |  |
| 520 |  | HVAHIQSGL | YIFGYHPHGI |  |  |  |
| 521 |  | HVAHIQSGL | YIFGYHPHGI |  |  |  |
| 522 |  | HVAKVAEFF | YIFGYHPHGI |  |  |  |
| 523 |  | HVAPQQNAL | YIFPSSISAM |  |  |  |
| 524 |  | HVGDGFEFM | YIFPSSISAM |  |  |  |
| 525 |  | HVGDGFEFM | YIFPSSISAM |  |  |  |
| 526 |  | HVGDGFEFM | YINDYGLIQI |  |  |  |
| 527 |  | HVIEKMHHV | YINDYGLIQI |  |  |  |
| 528 |  | HVNVIVERL | YINDYGLIQI |  |  |  |
| 529 |  | HVQEVQERL | YINEHDYAKA |  |  |  |
| 530 |  | HVQNMIKGV | YINEHDYAKA |  |  |  |
| 531 |  | HVSHLTEKL | YINESGHLNL |  |  |  |
| 532 |  | HVVDFFNRV | YINQGVSHAL |  |  |  |
| 533 |  | IAKALDLAI | YINQGVSHAL |  |  |  |
| 534 |  | IENKLKDLL | YINQGVSHAL |  |  |  |
| 535 |  | IENKLKDLL | YINQGVSHAL |  |  |  |
| 536 |  | IFEGLVKQI | YINRFQDAVL |  |  |  |
| 537 |  | IGREGAMLK | YISFFIGQEL |  |  |  |
| 538 |  | IGTQLMERL | YISFFIGQEL |  |  |  |
| 539 |  | IGTQLMERL | YISFFIGQEL |  |  |  |
| 540 |  | IGTQLMERL | YISTLDKEKL |  |  |  |
| 541 |  | IGTQLmERL | YISTLDKEKL |  |  |  |
| 542 |  | IIAALFKAL | YPFILPHQQV |  |  |  |
| 543 |  | IIADNIIFL | YPFILPHQQV |  |  |  |
| 544 |  | IIADNIIFL | YPLERGFIYV |  |  |  |
| 545 |  | IIADNIIFL | YPLERGFIYV |  |  |  |
| 546 |  | IIADNIIFL | YQQYQQQAGY |  |  |  |
| 547 |  | IIAPYRQQL | YREFWAKPKP |  |  |  |
| 548 |  | IIAPYRQQL | YSVNPNKVDD |  |  |  |
| 549 |  | IIASHWDDL | YVAPAYGVYL |  |  |  |
| 550 |  | IIDDTIFNL | YVAPAYGVYL |  |  |  |
| 551 |  | IIEKNFDYL | YVAQADLELL |  |  |  |
| 552 |  | IIEKNFDYL | YVAQAGLELL |  |  |  |
| 553 |  | IIEKNFDYL | YVISEGFLKA |  |  |  |
| 554 |  | IIEKNFDYL | YVISEGFLKA |  |  |  |
| 555 |  | IIFDDFREA | YVISEGFLKA |  |  |  |
| 556 |  | IIFDDFREA | YVISEGFLKA |  |  |  |
| 557 |  | IIFDDFREA | YVKVKVEPSG |  |  |  |
| 558 |  | IIGKVSTAL | YYRDDFYDRL |  |  |  |
| 559 |  | IIGKVSTAL |  |  |  |  |
| 560 |  | IIGQFGVGF |  |  |  |  |
| 561 |  | IIIALIDRL |  |  |  |  |
| 562 |  | IIIALIDRL |  |  |  |  |
| 563 |  | IIIALIDRL |  |  |  |  |
| 564 |  | IIIALIDRL |  |  |  |  |
| 565 |  | IIIIPEKQV |  |  |  |  |
| 566 |  | IIIPEIQKV |  |  |  |  |
| 567 |  | IIIPEIQKV |  |  |  |  |
| 568 |  | IIIPEIQKV |  |  |  |  |
| 569 |  | IIIPEIQKV |  |  |  |  |
| 570 |  | IIISEWMGY |  |  |  |  |
| 571 |  | IIISEWMGY |  |  |  |  |
| 572 |  | IIISEWMGY |  |  |  |  |
| 573 |  | IIISEWmGY |  |  |  |  |
| 574 |  | IIKETKEEV |  |  |  |  |
| 575 |  | IIKETKEEV |  |  |  |  |
| 576 |  | IIKIIGDLL |  |  |  |  |
| 577 |  | IIKKYFEEY |  |  |  |  |
| 578 |  | IIKKYFEEY |  |  |  |  |
| 579 |  | IIKKYFEEY |  |  |  |  |
| 580 |  | IIKQDINSL |  |  |  |  |
| 581 |  | IIKQDINSL |  |  |  |  |
| 582 |  | IIKQDINSL |  |  |  |  |
| 583 |  | IINAARESL |  |  |  |  |
| 584 |  | IINDLLEQL |  |  |  |  |
| 585 |  | IINDLLEQL |  |  |  |  |
| 586 |  | IINDLLEQL |  |  |  |  |
| 587 |  | IINDLLQSL |  |  |  |  |
| 588 |  | IINDLLQSL |  |  |  |  |
| 589 |  | IINDLLQSL |  |  |  |  |
| 590 |  | IINDLLQSL |  |  |  |  |
| 591 |  | IINGEATML |  |  |  |  |
| 592 |  | IINGEATML |  |  |  |  |
| 593 |  | IINGEATML |  |  |  |  |
| 594 |  | IINGIIISV |  |  |  |  |
| 595 |  | IINIMQDRL |  |  |  |  |
| 596 |  | IINIMQDRL |  |  |  |  |
| 597 |  | IINIMQDRL |  |  |  |  |
| 598 |  | IINPYRETV |  |  |  |  |
| 599 |  | IINPYRETV |  |  |  |  |
| 600 |  | IINPYRETV |  |  |  |  |
| 601 |  | IINSILNEV |  |  |  |  |
| 602 |  | IINSILNEV |  |  |  |  |
| 603 |  | IINSILNEV |  |  |  |  |
| 604 |  | IINSLVTTV |  |  |  |  |
| 605 |  | IINSLVTTV |  |  |  |  |
| 606 |  | IIQKHWRGY |  |  |  |  |
| 607 |  | IIQKHWRGY |  |  |  |  |
| 608 |  | IIQQAFINV |  |  |  |  |
| 609 |  | IIQQAFINV |  |  |  |  |
| 610 |  | IIQSIRMGM |  |  |  |  |
| 611 |  | IIQSIRMGM |  |  |  |  |
| 612 |  | IIQSIRMGM |  |  |  |  |
| 613 |  | IIQSIRMGM |  |  |  |  |
| 614 |  | IISEIISSL |  |  |  |  |
| 615 |  | IISPLFAEL |  |  |  |  |
| 616 |  | IITKEFILM |  |  |  |  |
| 617 |  | IITKEFILM |  |  |  |  |
| 618 |  | IITKEFILM |  |  |  |  |
| 619 |  | IITKEFILM |  |  |  |  |
| 620 |  | IIVDIFHGL |  |  |  |  |
| 621 |  | IIVDIFHGL |  |  |  |  |
| 622 |  | IIVDIFHGL |  |  |  |  |
| 623 |  | IIVDIFHGL |  |  |  |  |
| 624 |  | IIYHLFDAF |  |  |  |  |
| 625 |  | IIYHLFDAF |  |  |  |  |
| 626 |  | IIYQYMEEI |  |  |  |  |
| 627 |  | IIYQYMEEI |  |  |  |  |
| 628 |  | IIYQYMEEI |  |  |  |  |
| 629 |  | ILADIQSHM |  |  |  |  |
| 630 |  | ILFGHENRV |  |  |  |  |
| 631 |  | ILFGHENRV |  |  |  |  |
| 632 |  | ILFREWSNV |  |  |  |  |
| 633 |  | ILGPKPQGV |  |  |  |  |
| 634 |  | ILGPKPQGV |  |  |  |  |
| 635 |  | ILIDWLVQV |  |  |  |  |
| 636 |  | ILIDWLVQV |  |  |  |  |
| 637 |  | ILKEHITQL |  |  |  |  |
| 638 |  | ILKGDFNYL |  |  |  |  |
| 639 |  | ILKGDFNYL |  |  |  |  |
| 640 |  | ILKGDFNYL |  |  |  |  |
| 641 |  | ILKGDWLGL |  |  |  |  |
| 642 |  | ILKGDWLGL |  |  |  |  |
| 643 |  | ILKGDWLGL |  |  |  |  |
| 644 |  | ILKMATNYL |  |  |  |  |
| 645 |  | ILKQDFETL |  |  |  |  |
| 646 |  | ILKQDFETL |  |  |  |  |
| 647 |  | ILKQDFETL |  |  |  |  |
| 648 |  | ILLLNGHLL |  |  |  |  |
| 649 |  | ILMEHIHKL |  |  |  |  |
| 650 |  | ILMEHIHKL |  |  |  |  |
| 651 |  | ILMEHIHKL |  |  |  |  |
| 652 |  | ILMEHIHKL |  |  |  |  |
| 653 |  | ILNDNINLL |  |  |  |  |
| 654 |  | ILNKVEERM |  |  |  |  |
| 655 |  | ILNKVEERM |  |  |  |  |
| 656 |  | ILNKVEERM |  |  |  |  |
| 657 |  | ILNPLLTLL |  |  |  |  |
| 658 |  | ILNPLLTLL |  |  |  |  |
| 659 |  | ILNRIQDLL |  |  |  |  |
| 660 |  | ILQIITELI |  |  |  |  |
| 661 |  | ILTSIQSLL |  |  |  |  |
| 662 |  | ILVRIPGEI |  |  |  |  |
| 663 |  | IMKLELEKN |  |  |  |  |
| 664 |  | IMKLELEKN |  |  |  |  |
| 665 |  | IMNSILERM |  |  |  |  |
| 666 |  | IMQSLRDLG |  |  |  |  |
| 667 |  | INTIFSDYI |  |  |  |  |
| 668 |  | IPFSFSDRL |  |  |  |  |
| 669 |  | IPIKKSDPV |  |  |  |  |
| 670 |  | IPIKKSDPV |  |  |  |  |
| 671 |  | IPNEIIHAL |  |  |  |  |
| 672 |  | IPNEIIHAL |  |  |  |  |
| 673 |  | IPNEIIHAL |  |  |  |  |
| 674 |  | IPNEIIHAL |  |  |  |  |
| 675 |  | IPNRGIDLL |  |  |  |  |
| 676 |  | IPNRGIDLL |  |  |  |  |
| 677 |  | IPNRGIDLL |  |  |  |  |
| 678 |  | IPNRGIDLL |  |  |  |  |
| 679 |  | IQAYLDNVF |  |  |  |  |
| 680 |  | ISNDKFEYL |  |  |  |  |
| 681 |  | ISNDKFEYL |  |  |  |  |
| 682 |  | ISNDKFEYL |  |  |  |  |
| 683 |  | ISNKPTDKL |  |  |  |  |
| 684 |  | ITLLQSVSN |  |  |  |  |
| 685 |  | ITLLQSVSN |  |  |  |  |
| 686 |  | ITMKMVPKL |  |  |  |  |
| 687 |  | ITMKMVPKL |  |  |  |  |
| 688 |  | ITMKMVPKL |  |  |  |  |
| 689 |  | IVAKHTSAL |  |  |  |  |
| 690 |  | IVAKHTSAL |  |  |  |  |
| 691 |  | IVALLSHGV |  |  |  |  |
| 692 |  | IVALLSHGV |  |  |  |  |
| 693 |  | IVALLSHGV |  |  |  |  |
| 694 |  | IVAPGTFEV |  |  |  |  |
| 695 |  | IVHSVFEFL |  |  |  |  |
| 696 |  | IVIGVLHQL |  |  |  |  |
| 697 |  | IVIGVLHQL |  |  |  |  |
| 698 |  | IVIGVLHQL |  |  |  |  |
| 699 |  | IVIGVLHQL |  |  |  |  |
| 700 |  | IVKEIHEQL |  |  |  |  |
| 701 |  | IVKEIHEQL |  |  |  |  |
| 702 |  | IVKEIHEQL |  |  |  |  |
| 703 |  | IVKEVSTYI |  |  |  |  |
| 704 |  | IVKEVSTYI |  |  |  |  |
| 705 |  | IVKEVSTYI |  |  |  |  |
| 706 |  | IVKEVSTYI |  |  |  |  |
| 707 |  | IVKVTFEEL |  |  |  |  |
| 708 |  | IVKVTFEEL |  |  |  |  |
| 709 |  | IVNDKSQNL |  |  |  |  |
| 710 |  | IVNDKSQNL |  |  |  |  |
| 711 |  | IVNDKSQNL |  |  |  |  |
| 712 |  | IVNDKSQNL |  |  |  |  |
| 713 |  | IVNFVHTNL |  |  |  |  |
| 714 |  | IVNFVHTNL |  |  |  |  |
| 715 |  | IVNFVHTNL |  |  |  |  |
| 716 |  | IVNFVHTNL |  |  |  |  |
| 717 |  | IVNFVNQKL |  |  |  |  |
| 718 |  | IVNGQIHSV |  |  |  |  |
| 719 |  | IVNGQIHSV |  |  |  |  |
| 720 |  | IVNKAFGIN |  |  |  |  |
| 721 |  | IVNRTIANM |  |  |  |  |
| 722 |  | IVNRTIANM |  |  |  |  |
| 723 |  | IVNTSNESL |  |  |  |  |
| 724 |  | IVNTSNESL |  |  |  |  |
| 725 |  | IVNWVNETL |  |  |  |  |
| 726 |  | IVNWVNETL |  |  |  |  |
| 727 |  | IVNWVNETL |  |  |  |  |
| 728 |  | IVQKIEEVL |  |  |  |  |
| 729 |  | IVQKIEEVL |  |  |  |  |
| 730 |  | IVQPIISKL |  |  |  |  |
| 731 |  | IVQPIISKL |  |  |  |  |
| 732 |  | IVQPIISKL |  |  |  |  |
| 733 |  | IVQRLIEQI |  |  |  |  |
| 734 |  | IVRDIKEKL |  |  |  |  |
| 735 |  | IVRDIKEKL |  |  |  |  |
| 736 |  | IVRDIKEKL |  |  |  |  |
| 737 |  | IVSKEFQAL |  |  |  |  |
| 738 |  | IVSKEFQAL |  |  |  |  |
| 739 |  | IVSKEFQAL |  |  |  |  |
| 740 |  | IVSRQFVEM |  |  |  |  |
| 741 |  | IVSRQFVEM |  |  |  |  |
| 742 |  | IVSRQFVEM |  |  |  |  |
| 743 |  | IVVGRITEV |  |  |  |  |
| 744 |  | IVVGRITEV |  |  |  |  |
| 745 |  | IVVGRITEV |  |  |  |  |
| 746 |  | KAFDKHMNL |  |  |  |  |
| 747 |  | KAFQKEIGL |  |  |  |  |
| 748 |  | KAIDIYEQV |  |  |  |  |
| 749 |  | KAISRWSSL |  |  |  |  |
| 750 |  | KAKDNFNFL |  |  |  |  |
| 751 |  | KAKDNFNFL |  |  |  |  |
| 752 |  | KAKDNFNFL |  |  |  |  |
| 753 |  | KAKEWDEFL |  |  |  |  |
| 754 |  | KAKEWDEFL |  |  |  |  |
| 755 |  | KANEVISKL |  |  |  |  |
| 756 |  | KANEVISKL |  |  |  |  |
| 757 |  | KANEVISKL |  |  |  |  |
| 758 |  | KANEVISKL |  |  |  |  |
| 759 |  | KFAEAFEAI |  |  |  |  |
| 760 |  | KFAHLINVE |  |  |  |  |
| 761 |  | KFASFIDKV |  |  |  |  |
| 762 |  | KGVLRVEGF |  |  |  |  |
| 763 |  | KIADFGWSV |  |  |  |  |
| 764 |  | KIADFGWSV |  |  |  |  |
| 765 |  | KIADFGWSV |  |  |  |  |
| 766 |  | KIADFGWSV |  |  |  |  |
| 767 |  | KIADFLNSF |  |  |  |  |
| 768 |  | KIADFLNSF |  |  |  |  |
| 769 |  | KIADFLNSF |  |  |  |  |
| 770 |  | KIADFLNSF |  |  |  |  |
| 771 |  | KIADRFLLY |  |  |  |  |
| 772 |  | KIAEGVNSL |  |  |  |  |
| 773 |  | KIAELNTKL |  |  |  |  |
| 774 |  | KIAELNTKL |  |  |  |  |
| 775 |  | KIAELNTKL |  |  |  |  |
| 776 |  | KIAFIFNNL |  |  |  |  |
| 777 |  | KIAFIFNNL |  |  |  |  |
| 778 |  | KIAFIFNNL |  |  |  |  |
| 779 |  | KIAGYVTHL |  |  |  |  |
| 780 |  | KIAGYVTHL |  |  |  |  |
| 781 |  | KIAGYVTHL |  |  |  |  |
| 782 |  | KIAKESQFL |  |  |  |  |
| 783 |  | KIAKESQFL |  |  |  |  |
| 784 |  | KIAKESQFL |  |  |  |  |
| 785 |  | KIAPNTPQL |  |  |  |  |
| 786 |  | KIAPNTPQL |  |  |  |  |
| 787 |  | KIAPNTPQL |  |  |  |  |
| 788 |  | KIAPNTPQL |  |  |  |  |
| 789 |  | KIAPYSVEI |  |  |  |  |
| 790 |  | KIAQFLESV |  |  |  |  |
| 791 |  | KIAQLEEQL |  |  |  |  |
| 792 |  | KIAQLEEQL |  |  |  |  |
| 793 |  | KIEDYFPEF |  |  |  |  |
| 794 |  | KIEEIKDFL |  |  |  |  |
| 795 |  | KIEEIKDFL |  |  |  |  |
| 796 |  | KIEEIKDFL |  |  |  |  |
| 797 |  | KIEEIKDFL |  |  |  |  |
| 798 |  | KIEVSFREL |  |  |  |  |
| 799 |  | KIFEFKETL |  |  |  |  |
| 800 |  | KIFMEFRKL |  |  |  |  |
| 801 |  | KIFPAALQL |  |  |  |  |
| 802 |  | KIFPMGDRL |  |  |  |  |
| 803 |  | KIFPMGDRL |  |  |  |  |
| 804 |  | KIFPMGDRL |  |  |  |  |
| 805 |  | KIFPMGDRL |  |  |  |  |
| 806 |  | KIFQVAQEL |  |  |  |  |
| 807 |  | KIGEGTYGV |  |  |  |  |
| 808 |  | KIHPMAYQL |  |  |  |  |
| 809 |  | KIIDGLLVM |  |  |  |  |
| 810 |  | KIIDIFTTL |  |  |  |  |
| 811 |  | KIIDIQEKV |  |  |  |  |
| 812 |  | KIIDIQEKV |  |  |  |  |
| 813 |  | KIIDIQEKV |  |  |  |  |
| 814 |  | KIIDIQEKV |  |  |  |  |
| 815 |  | KIIEYSVYL |  |  |  |  |
| 816 |  | KIIGIMEEV |  |  |  |  |
| 817 |  | KIIGIMEEV |  |  |  |  |
| 818 |  | KIIGIMEEV |  |  |  |  |
| 819 |  | KIINIVEVM |  |  |  |  |
| 820 |  | KIINIVEVM |  |  |  |  |
| 821 |  | KIINIVEVM |  |  |  |  |
| 822 |  | KIINNTENL |  |  |  |  |
| 823 |  | KIKEIAVTV |  |  |  |  |
| 824 |  | KIKEIAVTV |  |  |  |  |
| 825 |  | KIKEWVDKY |  |  |  |  |
| 826 |  | KIKSLTEYL |  |  |  |  |
| 827 |  | KILDTFEKL |  |  |  |  |
| 828 |  | KILPTLEAV |  |  |  |  |
| 829 |  | KIMEAMEKL |  |  |  |  |
| 830 |  | KINALMHAL |  |  |  |  |
| 831 |  | KINALMHAL |  |  |  |  |
| 832 |  | KINEAFIEM |  |  |  |  |
| 833 |  | KINEAFIEM |  |  |  |  |
| 834 |  | KINEAFIEM |  |  |  |  |
| 835 |  | KINEAFIEM |  |  |  |  |
| 836 |  | KINEGLEHL |  |  |  |  |
| 837 |  | KINEGLEHL |  |  |  |  |
| 838 |  | KINEGLEHL |  |  |  |  |
| 839 |  | KINEIRTLI |  |  |  |  |
| 840 |  | KINEIRTLI |  |  |  |  |
| 841 |  | KINGIFEQL |  |  |  |  |
| 842 |  | KINGIFEQL |  |  |  |  |
| 843 |  | KINGIFEQL |  |  |  |  |
| 844 |  | KINNVIDNL |  |  |  |  |
| 845 |  | KINNVIDNL |  |  |  |  |
| 846 |  | KINNVIDNL |  |  |  |  |
| 847 |  | KINNVIDNL |  |  |  |  |
| 848 |  | KINNWIVQL |  |  |  |  |
| 849 |  | KINNWIVQL |  |  |  |  |
| 850 |  | KINPENSKL |  |  |  |  |
| 851 |  | KINQFIEEI |  |  |  |  |
| 852 |  | KINQFIEEI |  |  |  |  |
| 853 |  | KINQFIEEI |  |  |  |  |
| 854 |  | KIQEILTQV |  |  |  |  |
| 855 |  | KIQEILTQV |  |  |  |  |
| 856 |  | KIQEILTQV |  |  |  |  |
| 857 |  | KIQEILTQV |  |  |  |  |
| 858 |  | KIQEVFSSY |  |  |  |  |
| 859 |  | KISDIQSQL |  |  |  |  |
| 860 |  | KISDIQSQL |  |  |  |  |
| 861 |  | KISDIQSQL |  |  |  |  |
| 862 |  | KISDIQSQL |  |  |  |  |
| 863 |  | KISEMIQGL |  |  |  |  |
| 864 |  | KISEMIQGL |  |  |  |  |
| 865 |  | KISVIVETV |  |  |  |  |
| 866 |  | KISVIVETV |  |  |  |  |
| 867 |  | KISVIVETV |  |  |  |  |
| 868 |  | KITDAFSSL |  |  |  |  |
| 869 |  | KITDAFSSL |  |  |  |  |
| 870 |  | KITDAFSSL |  |  |  |  |
| 871 |  | KITDIIIFF |  |  |  |  |
| 872 |  | KITDLQNQL |  |  |  |  |
| 873 |  | KITDLQNQL |  |  |  |  |
| 874 |  | KITDLQNQL |  |  |  |  |
| 875 |  | KITDVIIGF |  |  |  |  |
| 876 |  | KITEDFRAL |  |  |  |  |
| 877 |  | KITEDFRAL |  |  |  |  |
| 878 |  | KITEDFRAL |  |  |  |  |
| 879 |  | KIVDKVQGL |  |  |  |  |
| 880 |  | KIWPGYSEL |  |  |  |  |
| 881 |  | KIYEGQVEV |  |  |  |  |
| 882 |  | KIYEGQVEV |  |  |  |  |
| 883 |  | KIYEGQVEV |  |  |  |  |
| 884 |  | KIYEGQVEV |  |  |  |  |
| 885 |  | KIYPGWLKV |  |  |  |  |
| 886 |  | KIYQWINEL |  |  |  |  |
| 887 |  | KLFDTQQFL |  |  |  |  |
| 888 |  | KLFELFDQL |  |  |  |  |
| 889 |  | KLFELFDQL |  |  |  |  |
| 890 |  | KLFNEFIQL |  |  |  |  |
| 891 |  | KLFNEFIQL |  |  |  |  |
| 892 |  | KLFNEFIQL |  |  |  |  |
| 893 |  | KLFNEFIQL |  |  |  |  |
| 894 |  | KLGELQEKL |  |  |  |  |
| 895 |  | KLIEFMDTF |  |  |  |  |
| 896 |  | KLKEIYENM |  |  |  |  |
| 897 |  | KLNAKLAEL |  |  |  |  |
| 898 |  | KLNAKLAEL |  |  |  |  |
| 899 |  | KLNAKLAEL |  |  |  |  |
| 900 |  | KLNAKLAEL |  |  |  |  |
| 901 |  | KLNATNIEL |  |  |  |  |
| 902 |  | KLNDLIQRL |  |  |  |  |
| 903 |  | KLNDLIQRL |  |  |  |  |
| 904 |  | KLNDLIQRL |  |  |  |  |
| 905 |  | KLNDLIQRL |  |  |  |  |
| 906 |  | KLNDLNSSV |  |  |  |  |
| 907 |  | KLNDLNSSV |  |  |  |  |
| 908 |  | KLNPQQFEV |  |  |  |  |
| 909 |  | KLNPQQFEV |  |  |  |  |
| 910 |  | KLQEFLQTL |  |  |  |  |
| 911 |  | KLSSIGIQV |  |  |  |  |
| 912 |  | KMLTSNLKI |  |  |  |  |
| 913 |  | KMLTSNLKI |  |  |  |  |
| 914 |  | KMLTSNLKI |  |  |  |  |
| 915 |  | KPEQVAKQI |  |  |  |  |
| 916 |  | KPFPFFEGL |  |  |  |  |
| 917 |  | KPFPFFEGL |  |  |  |  |
| 918 |  | KPFPFFEGL |  |  |  |  |
| 919 |  | KPNATQEEL |  |  |  |  |
| 920 |  | KQMEQVAQF |  |  |  |  |
| 921 |  | KTNIIRDYL |  |  |  |  |
| 922 |  | KTNIIRDYL |  |  |  |  |
| 923 |  | KVADALTNA |  |  |  |  |
| 924 |  | KVADGFKVV |  |  |  |  |
| 925 |  | KVADGFKVV |  |  |  |  |
| 926 |  | KVADGFKVV |  |  |  |  |
| 927 |  | KVADGFKVV |  |  |  |  |
| 928 |  | KVADGLVKV |  |  |  |  |
| 929 |  | KVADGLVKV |  |  |  |  |
| 930 |  | KVADLIHFL |  |  |  |  |
| 931 |  | KVADSKTLL |  |  |  |  |
| 932 |  | KVAEKLEAL |  |  |  |  |
| 933 |  | KVAEKLEAL |  |  |  |  |
| 934 |  | KVAEKLEAL |  |  |  |  |
| 935 |  | KVAELFEKL |  |  |  |  |
| 936 |  | KVAELFEKL |  |  |  |  |
| 937 |  | KVAELFEKL |  |  |  |  |
| 938 |  | KVAELFEKL |  |  |  |  |
| 939 |  | KVAELKQEL |  |  |  |  |
| 940 |  | KVAELLEKY |  |  |  |  |
| 941 |  | KVAELVEFL |  |  |  |  |
| 942 |  | KVAELVHFL |  |  |  |  |
| 943 |  | KVAELVHFL |  |  |  |  |
| 944 |  | KVAELVHFL |  |  |  |  |
| 945 |  | KVAELVHFL |  |  |  |  |
| 946 |  | KVALWLAGL |  |  |  |  |
| 947 |  | KVAPAPAVV |  |  |  |  |
| 948 |  | KVAPAPAVV |  |  |  |  |
| 949 |  | KVAPAPAVV |  |  |  |  |
| 950 |  | KVAPGFQFF |  |  |  |  |
| 951 |  | KVASLLHQV |  |  |  |  |
| 952 |  | KVASLLHQV |  |  |  |  |
| 953 |  | KVASLLHQV |  |  |  |  |
| 954 |  | KVATEFESF |  |  |  |  |
| 955 |  | KVATEFESF |  |  |  |  |
| 956 |  | KVATEFESF |  |  |  |  |
| 957 |  | KVFDAFLNM |  |  |  |  |
| 958 |  | KVFDAFLNM |  |  |  |  |
| 959 |  | KVFGNEIKL |  |  |  |  |
| 960 |  | KVFLLKEEL |  |  |  |  |
| 961 |  | KVFPSISKL |  |  |  |  |
| 962 |  | KVFSAFITV |  |  |  |  |
| 963 |  | KVFSAFITV |  |  |  |  |
| 964 |  | KVFSAFITV |  |  |  |  |
| 965 |  | KVFSAFITV |  |  |  |  |
| 966 |  | KVFSTPHGL |  |  |  |  |
| 967 |  | KVGEFFSHV |  |  |  |  |
| 968 |  | KVGPVPVLV |  |  |  |  |
| 969 |  | KVGPVPVLV |  |  |  |  |
| 970 |  | KVGPVPVLV |  |  |  |  |
| 971 |  | KVIDQQNGL |  |  |  |  |
| 972 |  | KVIDTQQKV |  |  |  |  |
| 973 |  | KVIDTQQKV |  |  |  |  |
| 974 |  | KVIDTQQKV |  |  |  |  |
| 975 |  | KVIDTQQKV |  |  |  |  |
| 976 |  | KVIGIIEHL |  |  |  |  |
| 977 |  | KVKAEVQNL |  |  |  |  |
| 978 |  | KVKDWVERL |  |  |  |  |
| 979 |  | KVKEFFEKL |  |  |  |  |
| 980 |  | KVKEFFEKL |  |  |  |  |
| 981 |  | KVKEFFEKL |  |  |  |  |
| 982 |  | KVKEKFQQL |  |  |  |  |
| 983 |  | KVKEKFQQL |  |  |  |  |
| 984 |  | KVKEKFQQL |  |  |  |  |
| 985 |  | KVKEKVQKL |  |  |  |  |
| 986 |  | KVKEKVQKL |  |  |  |  |
| 987 |  | KVKEKVQKL |  |  |  |  |
| 988 |  | KVKELGENL |  |  |  |  |
| 989 |  | KVKSEVNKL |  |  |  |  |
| 990 |  | KVLDFEHFL |  |  |  |  |
| 991 |  | KVLGIVVGV |  |  |  |  |
| 992 |  | KVLGIVVGV |  |  |  |  |
| 993 |  | KVLGIVVGV |  |  |  |  |
| 994 |  | KVNDILELY |  |  |  |  |
| 995 |  | KVNDILELY |  |  |  |  |
| 996 |  | KVNDILELY |  |  |  |  |
| 997 |  | KVNSHIAKL |  |  |  |  |
| 998 |  | KVPEWVDTV |  |  |  |  |
| 999 |  | KVPEWVDTV |  |  |  |  |
| 1000 |  | KVPEWVDTV |  |  |  |  |
| 1001 |  | KVPEWVDTV |  |  |  |  |
| 1002 |  | KVQAGNSSL |  |  |  |  |
| 1003 |  | KVQDIKNNL |  |  |  |  |
| 1004 |  | KVQDIKNNL |  |  |  |  |
| 1005 |  | KVQDIKNNL |  |  |  |  |
| 1006 |  | KVQDIKNNL |  |  |  |  |
| 1007 |  | KVSELKEEL |  |  |  |  |
| 1008 |  | KVSELKEEL |  |  |  |  |
| 1009 |  | KVSELVQFL |  |  |  |  |
| 1010 |  | KVSSGFSGV |  |  |  |  |
| 1011 |  | KVSSGFSGV |  |  |  |  |
| 1012 |  | KVTDAFARL |  |  |  |  |
| 1013 |  | KVTDAFARL |  |  |  |  |
| 1014 |  | KVTDAFARL |  |  |  |  |
| 1015 |  | KVTEAFDYL |  |  |  |  |
| 1016 |  | KVTEAFDYL |  |  |  |  |
| 1017 |  | KVTEAFDYL |  |  |  |  |
| 1018 |  | KVTEAFDYL |  |  |  |  |
| 1019 |  | LAAARLAAA |  |  |  |  |
| 1020 |  | LAAEQLYKL |  |  |  |  |
| 1021 |  | LAARLAFII |  |  |  |  |
| 1022 |  | LAKVINDGL |  |  |  |  |
| 1023 |  | LAKVINDGL |  |  |  |  |
| 1024 |  | LAKVINDGL |  |  |  |  |
| 1025 |  | LDFGSLSNL |  |  |  |  |
| 1026 |  | LDLQILEKL |  |  |  |  |
| 1027 |  | LEAEFQSLF |  |  |  |  |
| 1028 |  | LEQRFLELL |  |  |  |  |
| 1029 |  | LEQRFLELL |  |  |  |  |
| 1030 |  | LEQRFLELL |  |  |  |  |
| 1031 |  | LERQFLELL |  |  |  |  |
| 1032 |  | LFVINMNEI |  |  |  |  |
| 1033 |  | LIAEPFYHL |  |  |  |  |
| 1034 |  | LIAKVIQNL |  |  |  |  |
| 1035 |  | LIAKVIQNL |  |  |  |  |
| 1036 |  | LIAKVIQNL |  |  |  |  |
| 1037 |  | LIFPEFERL |  |  |  |  |
| 1038 |  | LIFPEFERL |  |  |  |  |
| 1039 |  | LIFPEFERL |  |  |  |  |
| 1040 |  | LIGEFLEKV |  |  |  |  |
| 1041 |  | LIGEFLEKV |  |  |  |  |
| 1042 |  | LIGEFLEKV |  |  |  |  |
| 1043 |  | LIHRMIEFV |  |  |  |  |
| 1044 |  | LIHSEISNL |  |  |  |  |
| 1045 |  | LIHSEISNL |  |  |  |  |
| 1046 |  | LIHSEISNL |  |  |  |  |
| 1047 |  | LIIGTFERM |  |  |  |  |
| 1048 |  | LIIGTFERM |  |  |  |  |
| 1049 |  | LIIGTFERM |  |  |  |  |
| 1050 |  | LIIQDFKGL |  |  |  |  |
| 1051 |  | LIKELPELL |  |  |  |  |
| 1052 |  | LIKSINEKF |  |  |  |  |
| 1053 |  | LIKSINEKF |  |  |  |  |
| 1054 |  | LINDKVAQL |  |  |  |  |
| 1055 |  | LINKEITKI |  |  |  |  |
| 1056 |  | LINKGFVEI |  |  |  |  |
| 1057 |  | LINKGFVEI |  |  |  |  |
| 1058 |  | LINKGFVEI |  |  |  |  |
| 1059 |  | LINKGFVEI |  |  |  |  |
| 1060 |  | LINKSGELL |  |  |  |  |
| 1061 |  | LINKSGELL |  |  |  |  |
| 1062 |  | LINKSGELL |  |  |  |  |
| 1063 |  | LINKSGELL |  |  |  |  |
| 1064 |  | LINLVGESL |  |  |  |  |
| 1065 |  | LINLVGESL |  |  |  |  |
| 1066 |  | LINPNIATV |  |  |  |  |
| 1067 |  | LINPNIATV |  |  |  |  |
| 1068 |  | LINQHLSGL |  |  |  |  |
| 1069 |  | LINQHLSGL |  |  |  |  |
| 1070 |  | LINQHLSGL |  |  |  |  |
| 1071 |  | LINQHLSGL |  |  |  |  |
| 1072 |  | LIPAFQNLL |  |  |  |  |
| 1073 |  | LIQAEARCL |  |  |  |  |
| 1074 |  | LIQDITQKL |  |  |  |  |
| 1075 |  | LIQDITQRL |  |  |  |  |
| 1076 |  | LIQNAFEKL |  |  |  |  |
| 1077 |  | LIQNAFEKL |  |  |  |  |
| 1078 |  | LIQSFTDRL |  |  |  |  |
| 1079 |  | LIQSFTDRL |  |  |  |  |
| 1080 |  | LIQSFTDRL |  |  |  |  |
| 1081 |  | LIQSFTDRL |  |  |  |  |
| 1082 |  | LISDGFHML |  |  |  |  |
| 1083 |  | LISELFQKL |  |  |  |  |
| 1084 |  | LISELFQKL |  |  |  |  |
| 1085 |  | LISKLFYSL |  |  |  |  |
| 1086 |  | LISQIVSSI |  |  |  |  |
| 1087 |  | LITGMFQRL |  |  |  |  |
| 1088 |  | LITGMFQRL |  |  |  |  |
| 1089 |  | LITGMFQRL |  |  |  |  |
| 1090 |  | LITGMFQRL |  |  |  |  |
| 1091 |  | LIVKEWNSV |  |  |  |  |
| 1092 |  | LIWNIKDEL |  |  |  |  |
| 1093 |  | LKAEDKSQL |  |  |  |  |
| 1094 |  | LKAEDKSQL |  |  |  |  |
| 1095 |  | LKFGTDLNQ |  |  |  |  |
| 1096 |  | LKKQVQELK |  |  |  |  |
| 1097 |  | LKKQVQELK |  |  |  |  |
| 1098 |  | LLADLLHNV |  |  |  |  |
| 1099 |  | LLEKDGDFL |  |  |  |  |
| 1100 |  | LLFSAFSRA |  |  |  |  |
| 1101 |  | LLGPAGARA |  |  |  |  |
| 1102 |  | LLGPPPVGV |  |  |  |  |
| 1103 |  | LLGPPPVGV |  |  |  |  |
| 1104 |  | LLGPPPVGV |  |  |  |  |
| 1105 |  | LLGPPPVGV |  |  |  |  |
| 1106 |  | LLIENVASL |  |  |  |  |
| 1107 |  | LLIENVASL |  |  |  |  |
| 1108 |  | LLIENVASL |  |  |  |  |
| 1109 |  | LLKDSIVQL |  |  |  |  |
| 1110 |  | LLKKNWQNV |  |  |  |  |
| 1111 |  | LLNEILEQV |  |  |  |  |
| 1112 |  | LLNEILEQV |  |  |  |  |
| 1113 |  | LLNEILEQV |  |  |  |  |
| 1114 |  | LLNEILEQV |  |  |  |  |
| 1115 |  | LLNNAFEEL |  |  |  |  |
| 1116 |  | LLNNAFEEL |  |  |  |  |
| 1117 |  | LLNNAFEEL |  |  |  |  |
| 1118 |  | LLNVINENF |  |  |  |  |
| 1119 |  | LLNVINENF |  |  |  |  |
| 1120 |  | LPAPKWTEL |  |  |  |  |
| 1121 |  | LPFGKVTNI |  |  |  |  |
| 1122 |  | LPNELIELL |  |  |  |  |
| 1123 |  | LQAKLEELQ |  |  |  |  |
| 1124 |  | LQAKLEELQ |  |  |  |  |
| 1125 |  | LQAKLEELQ |  |  |  |  |
| 1126 |  | LQAQLEKEL |  |  |  |  |
| 1127 |  | LQAQLTLER |  |  |  |  |
| 1128 |  | LQFAKEEAF |  |  |  |  |
| 1129 |  | LQISNEKNL |  |  |  |  |
| 1130 |  | LQRDEFIKI |  |  |  |  |
| 1131 |  | LQTRFPLDY |  |  |  |  |
| 1132 |  | LQTRFPLDY |  |  |  |  |
| 1133 |  | LRNPLIAGK |  |  |  |  |
| 1134 |  | LRNPLIAGK |  |  |  |  |
| 1135 |  | LSATVKEQI |  |  |  |  |
| 1136 |  | LSATVKEQI |  |  |  |  |
| 1137 |  | LSDQTKEKE |  |  |  |  |
| 1138 |  | LSDQTKEKE |  |  |  |  |
| 1139 |  | LSKQLTDLN |  |  |  |  |
| 1140 |  | LSLDDFIPG |  |  |  |  |
| 1141 |  | LSNKPSALH |  |  |  |  |
| 1142 |  | LSQRLQDIL |  |  |  |  |
| 1143 |  | LSQRLQDIL |  |  |  |  |
| 1144 |  | LSQRLQDIL |  |  |  |  |
| 1145 |  | LSQRLQDIL |  |  |  |  |
| 1146 |  | LSSKIMSSL |  |  |  |  |
| 1147 |  | LSSLPFQKI |  |  |  |  |
| 1148 |  | LSSLPFQKI |  |  |  |  |
| 1149 |  | LSSLPFQKI |  |  |  |  |
| 1150 |  | LSSLPFQKI |  |  |  |  |
| 1151 |  | LSVAPEFRR |  |  |  |  |
| 1152 |  | LTEAPLNPK |  |  |  |  |
| 1153 |  | LVAEIITHL |  |  |  |  |
| 1154 |  | LVAEVTQQL |  |  |  |  |
| 1155 |  | LVAEVTQQL |  |  |  |  |
| 1156 |  | LVANIIQNV |  |  |  |  |
| 1157 |  | LVANIIQNV |  |  |  |  |
| 1158 |  | LVASFSERV |  |  |  |  |
| 1159 |  | LVASFSERV |  |  |  |  |
| 1160 |  | LVASFSERV |  |  |  |  |
| 1161 |  | LVASFSERV |  |  |  |  |
| 1162 |  | LVFPGFFEL |  |  |  |  |
| 1163 |  | LVFPGFFEL |  |  |  |  |
| 1164 |  | LVFPGFFEL |  |  |  |  |
| 1165 |  | LVFPGFFEL |  |  |  |  |
| 1166 |  | LVFRDWPEL |  |  |  |  |
| 1167 |  | LVIVGLHQL |  |  |  |  |
| 1168 |  | LVIVGLHQL |  |  |  |  |
| 1169 |  | LVKDWAEFV |  |  |  |  |
| 1170 |  | LVKGFYPSD |  |  |  |  |
| 1171 |  | LVMGMFTRL |  |  |  |  |
| 1172 |  | LVMGMFTRL |  |  |  |  |
| 1173 |  | LVNKFIGYL |  |  |  |  |
| 1174 |  | LVPSKEQHL |  |  |  |  |
| 1175 |  | LVQDKFEVL |  |  |  |  |
| 1176 |  | LVQDKFEVL |  |  |  |  |
| 1177 |  | LVQVVMGFM |  |  |  |  |
| 1178 |  | LVQVVMGFM |  |  |  |  |
| 1179 |  | LVQVVMGFM |  |  |  |  |
| 1180 |  | LVVDWLESI |  |  |  |  |
| 1181 |  | LVVDWLESI |  |  |  |  |
| 1182 |  | LYALKSVKI |  |  |  |  |
| 1183 |  | MAAALFVLL |  |  |  |  |
| 1184 |  | MEKILILLL |  |  |  |  |
| 1185 |  | MEKMLFPLK |  |  |  |  |
| 1186 |  | MGERLLESK |  |  |  |  |
| 1187 |  | MIADGMIHL |  |  |  |  |
| 1188 |  | MIAILTENY |  |  |  |  |
| 1189 |  | MIAILTENY |  |  |  |  |
| 1190 |  | MIGQFGVGF |  |  |  |  |
| 1191 |  | MIGQFGVGF |  |  |  |  |
| 1192 |  | MIGQFGVGF |  |  |  |  |
| 1193 |  | MIGQFGVGF |  |  |  |  |
| 1194 |  | MIIENFEAL |  |  |  |  |
| 1195 |  | MIKAFEELL |  |  |  |  |
| 1196 |  | MIKAFEELL |  |  |  |  |
| 1197 |  | MINELAVAM |  |  |  |  |
| 1198 |  | MINELAVAM |  |  |  |  |
| 1199 |  | MINGFFDQF |  |  |  |  |
| 1200 |  | MINGFFDQF |  |  |  |  |
| 1201 |  | MINKDFEEY |  |  |  |  |
| 1202 |  | MINKDFEEY |  |  |  |  |
| 1203 |  | MINKDFEEY |  |  |  |  |
| 1204 |  | MINKQNEQM |  |  |  |  |
| 1205 |  | MINKQNEQM |  |  |  |  |
| 1206 |  | MINKQNEQM |  |  |  |  |
| 1207 |  | MINSSVHVI |  |  |  |  |
| 1208 |  | MINTLLALL |  |  |  |  |
| 1209 |  | MINTLLALL |  |  |  |  |
| 1210 |  | MINTLLALL |  |  |  |  |
| 1211 |  | MIPLLLAAL |  |  |  |  |
| 1212 |  | MIPTFTALL |  |  |  |  |
| 1213 |  | MISEYKANK |  |  |  |  |
| 1214 |  | MITDLEERL |  |  |  |  |
| 1215 |  | MLASMFHSL |  |  |  |  |
| 1216 |  | MLASMFHSL |  |  |  |  |
| 1217 |  | MLASMFHSL |  |  |  |  |
| 1218 |  | MLFTQPNIE |  |  |  |  |
| 1219 |  | MLIEVIEKL |  |  |  |  |
| 1220 |  | MLIEVIEKL |  |  |  |  |
| 1221 |  | MLMNLISRL |  |  |  |  |
| 1222 |  | MLPSILNQL |  |  |  |  |
| 1223 |  | MLSYLRAQL |  |  |  |  |
| 1224 |  | MPLGYVPPT |  |  |  |  |
| 1225 |  | MPNPSLAQV |  |  |  |  |
| 1226 |  | MPNPSLAQV |  |  |  |  |
| 1227 |  | mPNTRPPII |  |  |  |  |
| 1228 |  | MPNYPFINI |  |  |  |  |
| 1229 |  | MQMLIQMKL |  |  |  |  |
| 1230 |  | MQMLIQMKL |  |  |  |  |
| 1231 |  | MRSKEQILE |  |  |  |  |
| 1232 |  | MRYVASYLL |  |  |  |  |
| 1233 |  | MRYVASYLL |  |  |  |  |
| 1234 |  | MRYVASYLL |  |  |  |  |
| 1235 |  | MVADKFTEL |  |  |  |  |
| 1236 |  | MVADKFTEL |  |  |  |  |
| 1237 |  | MVADKFTEL |  |  |  |  |
| 1238 |  | MVADKFTEL |  |  |  |  |
| 1239 |  | MVADRFSEL |  |  |  |  |
| 1240 |  | MVADRFSEL |  |  |  |  |
| 1241 |  | MVARLGLGL |  |  |  |  |
| 1242 |  | MVHPVAERL |  |  |  |  |
| 1243 |  | MVHPVAERL |  |  |  |  |
| 1244 |  | MVHPVAERL |  |  |  |  |
| 1245 |  | MVNFIKENL |  |  |  |  |
| 1246 |  | MVNHFIAEF |  |  |  |  |
| 1247 |  | MVNQMTEKV |  |  |  |  |
| 1248 |  | MVNQMTEKV |  |  |  |  |
| 1249 |  | MVVDIVQEL |  |  |  |  |
| 1250 |  | NIAILNNNL |  |  |  |  |
| 1251 |  | NIAILNNNL |  |  |  |  |
| 1252 |  | NIANHFFTV |  |  |  |  |
| 1253 |  | NIAQVLAEL |  |  |  |  |
| 1254 |  | NIAQVLAEL |  |  |  |  |
| 1255 |  | NIAQVLAEL |  |  |  |  |
| 1256 |  | NIAVLKHNL |  |  |  |  |
| 1257 |  | NIAVLKHNL |  |  |  |  |
| 1258 |  | NIISTFQNL |  |  |  |  |
| 1259 |  | NIISTFQNL |  |  |  |  |
| 1260 |  | NIKDSFEKV |  |  |  |  |
| 1261 |  | NIKFIGELF |  |  |  |  |
| 1262 |  | NILmSMSLN |  |  |  |  |
| 1263 |  | NINRIKDQY |  |  |  |  |
| 1264 |  | NLASFIEQV |  |  |  |  |
| 1265 |  | NLLDNLNLL |  |  |  |  |
| 1266 |  | NLLDNLNLL |  |  |  |  |
| 1267 |  | NLLDNLNLL |  |  |  |  |
| 1268 |  | NLQKIKEKL |  |  |  |  |
| 1269 |  | NPISTVTEL |  |  |  |  |
| 1270 |  | NVIESFTEL |  |  |  |  |
| 1271 |  | NVKEFKEHI |  |  |  |  |
| 1272 |  | NVNAFIERL |  |  |  |  |
| 1273 |  | NVQNKNSSY |  |  |  |  |
| 1274 |  | NYLDQEIKI |  |  |  |  |
| 1275 |  | PAEFTPAVH |  |  |  |  |
| 1276 |  | PEKELLLLI |  |  |  |  |
| 1277 |  | PEKELLLLI |  |  |  |  |
| 1278 |  | PGHLQEGFG |  |  |  |  |
| 1279 |  | PGHLQEGFG |  |  |  |  |
| 1280 |  | PVLPRLDPE |  |  |  |  |
| 1281 |  | PVLPRLDPE |  |  |  |  |
| 1282 |  | PVLPRLDPE |  |  |  |  |
| 1283 |  | QIADLQQKL |  |  |  |  |
| 1284 |  | QIAEFKEAF |  |  |  |  |
| 1285 |  | QIAEFKEAF |  |  |  |  |
| 1286 |  | QIAEFKEAF |  |  |  |  |
| 1287 |  | QIAEFKEAF |  |  |  |  |
| 1288 |  | QIAKIFSSL |  |  |  |  |
| 1289 |  | QIFHPEQLI |  |  |  |  |
| 1290 |  | QIGPPEVHL |  |  |  |  |
| 1291 |  | QIGPPEVHL |  |  |  |  |
| 1292 |  | QIGPPEVHL |  |  |  |  |
| 1293 |  | QIGPPEVHL |  |  |  |  |
| 1294 |  | QIHAFVDYL |  |  |  |  |
| 1295 |  | QIHAFVDYL |  |  |  |  |
| 1296 |  | QIHAFVDYL |  |  |  |  |
| 1297 |  | QIIKNITTL |  |  |  |  |
| 1298 |  | QIIKNITTL |  |  |  |  |
| 1299 |  | QIIKNITTL |  |  |  |  |
| 1300 |  | QIIQGIQQL |  |  |  |  |
| 1301 |  | QIKEIKEVI |  |  |  |  |
| 1302 |  | QIKEIKEVI |  |  |  |  |
| 1303 |  | QIKEIKEVI |  |  |  |  |
| 1304 |  | QIKKWAEKL |  |  |  |  |
| 1305 |  | QILNIIERL |  |  |  |  |
| 1306 |  | QILNIIERL |  |  |  |  |
| 1307 |  | QILNIIERL |  |  |  |  |
| 1308 |  | QILNIIERL |  |  |  |  |
| 1309 |  | QILTDFPKL |  |  |  |  |
| 1310 |  | QINKFFEAL |  |  |  |  |
| 1311 |  | QINKIIEQL |  |  |  |  |
| 1312 |  | QINKIIEQL |  |  |  |  |
| 1313 |  | QINKIIEQL |  |  |  |  |
| 1314 |  | QINKIIEQL |  |  |  |  |
| 1315 |  | QINTSWHTL |  |  |  |  |
| 1316 |  | QIQDQFNDL |  |  |  |  |
| 1317 |  | QIQEFKEAF |  |  |  |  |
| 1318 |  | QIQEFKEAF |  |  |  |  |
| 1319 |  | QIQEFKEAF |  |  |  |  |
| 1320 |  | QIQEFKEAF |  |  |  |  |
| 1321 |  | QIQKIIETL |  |  |  |  |
| 1322 |  | QIQKIIETL |  |  |  |  |
| 1323 |  | QIQKIIETL |  |  |  |  |
| 1324 |  | QIQKIIETL |  |  |  |  |
| 1325 |  | QISPFFPLV |  |  |  |  |
| 1326 |  | QISPFFPLV |  |  |  |  |
| 1327 |  | QISPFFPLV |  |  |  |  |
| 1328 |  | QISRIFQTL |  |  |  |  |
| 1329 |  | QITQINHGL |  |  |  |  |
| 1330 |  | QITQINHGL |  |  |  |  |
| 1331 |  | QIVDPWERL |  |  |  |  |
| 1332 |  | QIVELIEKI |  |  |  |  |
| 1333 |  | QIVELIEKI |  |  |  |  |
| 1334 |  | QIYCRLMKI |  |  |  |  |
| 1335 |  | QIYDIFQKL |  |  |  |  |
| 1336 |  | QIYDIFQKL |  |  |  |  |
| 1337 |  | QIYDIFQKL |  |  |  |  |
| 1338 |  | QIYDIFQKL |  |  |  |  |
| 1339 |  | QIYEIFQKL |  |  |  |  |
| 1340 |  | QIYEIFQKL |  |  |  |  |
| 1341 |  | QIYIYNEKI |  |  |  |  |
| 1342 |  | QIYQLIEYL |  |  |  |  |
| 1343 |  | QLAQFVHEV |  |  |  |  |
| 1344 |  | QLFSVVTLK |  |  |  |  |
| 1345 |  | QLFSVVTLK |  |  |  |  |
| 1346 |  | QLIVGWREL |  |  |  |  |
| 1347 |  | QLIVGWREL |  |  |  |  |
| 1348 |  | QLNEKVAQL |  |  |  |  |
| 1349 |  | QLNEKVAQL |  |  |  |  |
| 1350 |  | QLNEKVAQL |  |  |  |  |
| 1351 |  | QLNEKVAQL |  |  |  |  |
| 1352 |  | QNHKLIHEL |  |  |  |  |
| 1353 |  | QTNRLQEAL |  |  |  |  |
| 1354 |  | QVADFKDAL |  |  |  |  |
| 1355 |  | QVADFKDAL |  |  |  |  |
| 1356 |  | QVADFKDAL |  |  |  |  |
| 1357 |  | QVADFKDAL |  |  |  |  |
| 1358 |  | QVALLTERL |  |  |  |  |
| 1359 |  | QVALLTERL |  |  |  |  |
| 1360 |  | QVALLTERL |  |  |  |  |
| 1361 |  | QVAVISERL |  |  |  |  |
| 1362 |  | QVAVISERL |  |  |  |  |
| 1363 |  | QVAVISERL |  |  |  |  |
| 1364 |  | QVFEGQNRI |  |  |  |  |
| 1365 |  | QVFEKMHRM |  |  |  |  |
| 1366 |  | QVIAGFNRL |  |  |  |  |
| 1367 |  | QVIAGFNRL |  |  |  |  |
| 1368 |  | QVIAGFNRL |  |  |  |  |
| 1369 |  | QVIAGFNRL |  |  |  |  |
| 1370 |  | QVMQIIEHL |  |  |  |  |
| 1371 |  | QVNDLKERL |  |  |  |  |
| 1372 |  | QVNNLLEKI |  |  |  |  |
| 1373 |  | QVNNLLEKI |  |  |  |  |
| 1374 |  | QVQPFFDNI |  |  |  |  |
| 1375 |  | QVQPFFDNI |  |  |  |  |
| 1376 |  | RAIDSIHQL |  |  |  |  |
| 1377 |  | REEHLLEQL |  |  |  |  |
| 1378 |  | RFFDSFGNL |  |  |  |  |
| 1379 |  | RIADQFLGA |  |  |  |  |
| 1380 |  | RIADQFLGA |  |  |  |  |
| 1381 |  | RIADQFLGA |  |  |  |  |
| 1382 |  | RIAEALSKM |  |  |  |  |
| 1383 |  | RIAEALSKM |  |  |  |  |
| 1384 |  | RIAEFTTNL |  |  |  |  |
| 1385 |  | RIAELLTEL |  |  |  |  |
| 1386 |  | RIAELLTEL |  |  |  |  |
| 1387 |  | RIAELLTEL |  |  |  |  |
| 1388 |  | RIAELLTEL |  |  |  |  |
| 1389 |  | RIAEVLNGL |  |  |  |  |
| 1390 |  | RIAGEASRL |  |  |  |  |
| 1391 |  | RIATEFNQL |  |  |  |  |
| 1392 |  | RIATEFNQL |  |  |  |  |
| 1393 |  | RIATEFNQL |  |  |  |  |
| 1394 |  | RIATEFNQL |  |  |  |  |
| 1395 |  | RIFAPNHVV |  |  |  |  |
| 1396 |  | RIFGGLDML |  |  |  |  |
| 1397 |  | RIFKDFmHV |  |  |  |  |
| 1398 |  | RIHPVSTMV |  |  |  |  |
| 1399 |  | RIHPVSTMV |  |  |  |  |
| 1400 |  | RIHPVSTMV |  |  |  |  |
| 1401 |  | RIHPVSTMV |  |  |  |  |
| 1402 |  | RIIDVVYNA |  |  |  |  |
| 1403 |  | RIIDVVYNA |  |  |  |  |
| 1404 |  | RIIDVVYNA |  |  |  |  |
| 1405 |  | RIIDVVYNA |  |  |  |  |
| 1406 |  | RIIQSWEKI |  |  |  |  |
| 1407 |  | RIIQSWEKI |  |  |  |  |
| 1408 |  | RIKAINTFF |  |  |  |  |
| 1409 |  | RIKDEFQFL |  |  |  |  |
| 1410 |  | RIKDEFQFL |  |  |  |  |
| 1411 |  | RIKDEFQFL |  |  |  |  |
| 1412 |  | RIKDEFQFL |  |  |  |  |
| 1413 |  | RIKDEFQLL |  |  |  |  |
| 1414 |  | RIKDEFQLL |  |  |  |  |
| 1415 |  | RIKDEFQLL |  |  |  |  |
| 1416 |  | RIKDEFQLL |  |  |  |  |
| 1417 |  | RIKEEFQFL |  |  |  |  |
| 1418 |  | RIKGDWIGL |  |  |  |  |
| 1419 |  | RIKQITEEV |  |  |  |  |
| 1420 |  | RIKQITEEV |  |  |  |  |
| 1421 |  | RIKQITEEV |  |  |  |  |
| 1422 |  | RILDILEYI |  |  |  |  |
| 1423 |  | RINESLSQL |  |  |  |  |
| 1424 |  | RINKELSDL |  |  |  |  |
| 1425 |  | RINNEIDQL |  |  |  |  |
| 1426 |  | RIPSFVTEL |  |  |  |  |
| 1427 |  | RIQAQFAQL |  |  |  |  |
| 1428 |  | RIQAQFAQL |  |  |  |  |
| 1429 |  | RIQEFIELI |  |  |  |  |
| 1430 |  | RIQEFIELI |  |  |  |  |
| 1431 |  | RIQEFIELI |  |  |  |  |
| 1432 |  | RIQEFIELI |  |  |  |  |
| 1433 |  | RIQEIIEQL |  |  |  |  |
| 1434 |  | RIQEIIEQL |  |  |  |  |
| 1435 |  | RIQETQAEL |  |  |  |  |
| 1436 |  | RIQETQAEL |  |  |  |  |
| 1437 |  | RIQGGWTTI |  |  |  |  |
| 1438 |  | RIQVRFAEL |  |  |  |  |
| 1439 |  | RISDAITHL |  |  |  |  |
| 1440 |  | RISDAITHL |  |  |  |  |
| 1441 |  | RISDAITHL |  |  |  |  |
| 1442 |  | RISDAITHL |  |  |  |  |
| 1443 |  | RISDFHETY |  |  |  |  |
| 1444 |  | RISDFHETY |  |  |  |  |
| 1445 |  | RISDFHETY |  |  |  |  |
| 1446 |  | RISDFHETY |  |  |  |  |
| 1447 |  | RISEQFTAM |  |  |  |  |
| 1448 |  | RISPWLLRV |  |  |  |  |
| 1449 |  | RISVYFPTL |  |  |  |  |
| 1450 |  | RISVYFPTL |  |  |  |  |
| 1451 |  | RISVYFPTL |  |  |  |  |
| 1452 |  | RISVYFPTL |  |  |  |  |
| 1453 |  | RITDLYTDL |  |  |  |  |
| 1454 |  | RITILQEKL |  |  |  |  |
| 1455 |  | RITILQEKL |  |  |  |  |
| 1456 |  | RITILQEKL |  |  |  |  |
| 1457 |  | RIYFGEVQL |  |  |  |  |
| 1458 |  | RIYGKFLGL |  |  |  |  |
| 1459 |  | RIYGKFLGL |  |  |  |  |
| 1460 |  | RIYGKFLGL |  |  |  |  |
| 1461 |  | RIYGKFLGL |  |  |  |  |
| 1462 |  | RIYPTFLHL |  |  |  |  |
| 1463 |  | RLAPTFSNL |  |  |  |  |
| 1464 |  | RLATLNEKL |  |  |  |  |
| 1465 |  | RLAVYIDRV |  |  |  |  |
| 1466 |  | RLFPGSSFL |  |  |  |  |
| 1467 |  | RLNDFASTV |  |  |  |  |
| 1468 |  | RLNKVISEL |  |  |  |  |
| 1469 |  | RLNKVISEL |  |  |  |  |
| 1470 |  | RLNKVISEL |  |  |  |  |
| 1471 |  | RLNKVISEL |  |  |  |  |
| 1472 |  | RLQTWWHGV |  |  |  |  |
| 1473 |  | RLYQGINQL |  |  |  |  |
| 1474 |  | RPIDHWDAV |  |  |  |  |
| 1475 |  | RPIDHWDAV |  |  |  |  |
| 1476 |  | RPIDHWDAV |  |  |  |  |
| 1477 |  | RPNDIMAEV |  |  |  |  |
| 1478 |  | RSLGQNPTE |  |  |  |  |
| 1479 |  | RVADFTEQY |  |  |  |  |
| 1480 |  | RVADFTEQY |  |  |  |  |
| 1481 |  | RVADILTQL |  |  |  |  |
| 1482 |  | RVADILTQL |  |  |  |  |
| 1483 |  | RVADILTQL |  |  |  |  |
| 1484 |  | RVADILTQL |  |  |  |  |
| 1485 |  | RVADLTEQY |  |  |  |  |
| 1486 |  | RVADLTEQY |  |  |  |  |
| 1487 |  | RVADLTEQY |  |  |  |  |
| 1488 |  | RVADVIAQV |  |  |  |  |
| 1489 |  | RVADVIAQV |  |  |  |  |
| 1490 |  | RVADVIAQV |  |  |  |  |
| 1491 |  | RVAEFHTEL |  |  |  |  |
| 1492 |  | RVAEFHTEL |  |  |  |  |
| 1493 |  | RVAEFHTEL |  |  |  |  |
| 1494 |  | RVAEFHTEL |  |  |  |  |
| 1495 |  | RVAGIFPRL |  |  |  |  |
| 1496 |  | RVAGIFPRL |  |  |  |  |
| 1497 |  | RVAGIHKKV |  |  |  |  |
| 1498 |  | RVAGIHKKV |  |  |  |  |
| 1499 |  | RVAGIHKKV |  |  |  |  |
| 1500 |  | RVIDYILDL |  |  |  |  |
| 1501 |  | RVIDYILDL |  |  |  |  |
| 1502 |  | RVIDYILDL |  |  |  |  |
| 1503 |  | RVIDYILDL |  |  |  |  |
| 1504 |  | RVIEPFSRV |  |  |  |  |
| 1505 |  | RVIEPFSRV |  |  |  |  |
| 1506 |  | RVIEPFSRV |  |  |  |  |
| 1507 |  | RVIEPFSRV |  |  |  |  |
| 1508 |  | RVIGTLEEV |  |  |  |  |
| 1509 |  | RVKDDIESL |  |  |  |  |
| 1510 |  | RVKLILEYV |  |  |  |  |
| 1511 |  | RVMEYINRL |  |  |  |  |
| 1512 |  | RVMEYINRL |  |  |  |  |
| 1513 |  | RVMEYINRL |  |  |  |  |
| 1514 |  | RVNAVKTKV |  |  |  |  |
| 1515 |  | RVNENTHNL |  |  |  |  |
| 1516 |  | RVNENTHNL |  |  |  |  |
| 1517 |  | RVNETTEFL |  |  |  |  |
| 1518 |  | RVNETTEFL |  |  |  |  |
| 1519 |  | RVNETTEFL |  |  |  |  |
| 1520 |  | RVNILFDFV |  |  |  |  |
| 1521 |  | RVNILFDFV |  |  |  |  |
| 1522 |  | RVNKAFVNV |  |  |  |  |
| 1523 |  | RVNNFGSGL |  |  |  |  |
| 1524 |  | RVNNFGSGL |  |  |  |  |
| 1525 |  | RVQDAFAAA |  |  |  |  |
| 1526 |  | RVQDLMAQM |  |  |  |  |
| 1527 |  | RVQDLMAQM |  |  |  |  |
| 1528 |  | RVQDLMAQM |  |  |  |  |
| 1529 |  | RVQYFWEAL |  |  |  |  |
| 1530 |  | RVQYFWEAL |  |  |  |  |
| 1531 |  | RVSDINFTL |  |  |  |  |
| 1532 |  | RVVDLITNL |  |  |  |  |
| 1533 |  | RVVDYLTKL |  |  |  |  |
| 1534 |  | RVYEFLDKL |  |  |  |  |
| 1535 |  | RVYEFLDKL |  |  |  |  |
| 1536 |  | RVYEFLDKL |  |  |  |  |
| 1537 |  | RVYEFLDKL |  |  |  |  |
| 1538 |  | SAFSSWEEL |  |  |  |  |
| 1539 |  | SANDGFVRL |  |  |  |  |
| 1540 |  | SANDGFVRL |  |  |  |  |
| 1541 |  | SANDGFVRL |  |  |  |  |
| 1542 |  | SANDGFVRL |  |  |  |  |
| 1543 |  | SDYPPLGRF |  |  |  |  |
| 1544 |  | SEVQNYAIL |  |  |  |  |
| 1545 |  | SFKDEWTQL |  |  |  |  |
| 1546 |  | SIAAFIQRL |  |  |  |  |
| 1547 |  | SIAAFIQRL |  |  |  |  |
| 1548 |  | SIAAFIQRL |  |  |  |  |
| 1549 |  | SIAAFIQRL |  |  |  |  |
| 1550 |  | SIADKIQQI |  |  |  |  |
| 1551 |  | SIAEVVHQL |  |  |  |  |
| 1552 |  | SIAEVVHQL |  |  |  |  |
| 1553 |  | SIAEVVHQL |  |  |  |  |
| 1554 |  | SIAEVVHQL |  |  |  |  |
| 1555 |  | SIAGIFKEV |  |  |  |  |
| 1556 |  | SIAGIFKEV |  |  |  |  |
| 1557 |  | SIAGIFKEV |  |  |  |  |
| 1558 |  | SIAGIFKEV |  |  |  |  |
| 1559 |  | SIFAPFLTL |  |  |  |  |
| 1560 |  | SIFDDFAHY |  |  |  |  |
| 1561 |  | SIFDDFSHY |  |  |  |  |
| 1562 |  | SIFDDFSHY |  |  |  |  |
| 1563 |  | SIFEFVHAL |  |  |  |  |
| 1564 |  | SIFKWFSDL |  |  |  |  |
| 1565 |  | SIFPVFDNL |  |  |  |  |
| 1566 |  | SIFSAFLSV |  |  |  |  |
| 1567 |  | SIFSAFLSV |  |  |  |  |
| 1568 |  | SIHVGQAGV |  |  |  |  |
| 1569 |  | SIHVGQAGV |  |  |  |  |
| 1570 |  | SIHVGQAGV |  |  |  |  |
| 1571 |  | SIHVGQAGV |  |  |  |  |
| 1572 |  | SIIGRLLEV |  |  |  |  |
| 1573 |  | SIIGRLLEV |  |  |  |  |
| 1574 |  | SIIGRLLEV |  |  |  |  |
| 1575 |  | SIIGRLLEV |  |  |  |  |
| 1576 |  | SIINFFERL |  |  |  |  |
| 1577 |  | SIINFFERL |  |  |  |  |
| 1578 |  | SIKAFAEKL |  |  |  |  |
| 1579 |  | SIKAFAEKL |  |  |  |  |
| 1580 |  | SIKAFAEKL |  |  |  |  |
| 1581 |  | SIKAFAEKL |  |  |  |  |
| 1582 |  | SIKEFFRGL |  |  |  |  |
| 1583 |  | SIKKIADFL |  |  |  |  |
| 1584 |  | SIKKIADFL |  |  |  |  |
| 1585 |  | SIKNFEEFF |  |  |  |  |
| 1586 |  | SIKNFEEFF |  |  |  |  |
| 1587 |  | SIKNFEEFF |  |  |  |  |
| 1588 |  | SIKTGFQAV |  |  |  |  |
| 1589 |  | SIKTGFQAV |  |  |  |  |
| 1590 |  | SIKTGFQAV |  |  |  |  |
| 1591 |  | SIMSFFKTL |  |  |  |  |
| 1592 |  | SINAGFQSL |  |  |  |  |
| 1593 |  | SINAGFQSL |  |  |  |  |
| 1594 |  | SINAGFQSL |  |  |  |  |
| 1595 |  | SINAGFQSL |  |  |  |  |
| 1596 |  | SINDKIIEL |  |  |  |  |
| 1597 |  | SINDKIIEL |  |  |  |  |
| 1598 |  | SINEFWNKF |  |  |  |  |
| 1599 |  | SINKDWWKV |  |  |  |  |
| 1600 |  | SINQGLDRL |  |  |  |  |
| 1601 |  | SINQKWVEL |  |  |  |  |
| 1602 |  | SINQKWVEL |  |  |  |  |
| 1603 |  | SINSIKSRL |  |  |  |  |
| 1604 |  | SINSILDYI |  |  |  |  |
| 1605 |  | SINSILDYI |  |  |  |  |
| 1606 |  | SINSILDYI |  |  |  |  |
| 1607 |  | SINSRFAKV |  |  |  |  |
| 1608 |  | SINSRFAKV |  |  |  |  |
| 1609 |  | SINSRFAKV |  |  |  |  |
| 1610 |  | SIQKITDNI |  |  |  |  |
| 1611 |  | SIQKITDNI |  |  |  |  |
| 1612 |  | SIQKITDNI |  |  |  |  |
| 1613 |  | SIQQSIERL |  |  |  |  |
| 1614 |  | SKMIELSKV |  |  |  |  |
| 1615 |  | SKYPLLNTV |  |  |  |  |
| 1616 |  | SLAKIYTEA |  |  |  |  |
| 1617 |  | SLAPHFNSL |  |  |  |  |
| 1618 |  | SLAQYLINV |  |  |  |  |
| 1619 |  | SLDKFLASV |  |  |  |  |
| 1620 |  | SLFPFIEKL |  |  |  |  |
| 1621 |  | SLKSWNETL |  |  |  |  |
| 1622 |  | SLQGSWVEL |  |  |  |  |
| 1623 |  | SLSTYFKAL |  |  |  |  |
| 1624 |  | SLYKSFLQL |  |  |  |  |
| 1625 |  | SPIENIQRV |  |  |  |  |
| 1626 |  | SPIENIQRV |  |  |  |  |
| 1627 |  | SPIENIQRV |  |  |  |  |
| 1628 |  | SPIENIQRV |  |  |  |  |
| 1629 |  | STKNWTEKI |  |  |  |  |
| 1630 |  | SVAAVEERL |  |  |  |  |
| 1631 |  | SVAAVEERL |  |  |  |  |
| 1632 |  | SVAAVEERL |  |  |  |  |
| 1633 |  | SVADLAHVL |  |  |  |  |
| 1634 |  | SVADLAHVL |  |  |  |  |
| 1635 |  | SVADLAHVL |  |  |  |  |
| 1636 |  | SVADYFKQI |  |  |  |  |
| 1637 |  | SVAEKLSKL |  |  |  |  |
| 1638 |  | SVAEKLSKL |  |  |  |  |
| 1639 |  | SVAKTSQLL |  |  |  |  |
| 1640 |  | SVAKTSQLL |  |  |  |  |
| 1641 |  | SVASIKTGF |  |  |  |  |
| 1642 |  | SVASIKTGF |  |  |  |  |
| 1643 |  | SVASIKTGF |  |  |  |  |
| 1644 |  | SVASIKTGF |  |  |  |  |
| 1645 |  | SVATKLTAV |  |  |  |  |
| 1646 |  | SVAVIHQSL |  |  |  |  |
| 1647 |  | SVAVIHQSL |  |  |  |  |
| 1648 |  | SVAVIHQSL |  |  |  |  |
| 1649 |  | SVFAHPRKL |  |  |  |  |
| 1650 |  | SVFGQVERA |  |  |  |  |
| 1651 |  | SVFGQVERA |  |  |  |  |
| 1652 |  | SVFKDFYEL |  |  |  |  |
| 1653 |  | SVFKDFYEL |  |  |  |  |
| 1654 |  | SVFKDFYEL |  |  |  |  |
| 1655 |  | SVFPGARLL |  |  |  |  |
| 1656 |  | SVFSRFYGL |  |  |  |  |
| 1657 |  | SVFSRFYGL |  |  |  |  |
| 1658 |  | SVHKGFAFV |  |  |  |  |
| 1659 |  | SVHKGFAFV |  |  |  |  |
| 1660 |  | SVHKGFAFV |  |  |  |  |
| 1661 |  | SVHKGFAFV |  |  |  |  |
| 1662 |  | SVINFLDQL |  |  |  |  |
| 1663 |  | SVKEFKEEI |  |  |  |  |
| 1664 |  | SVKEFKEEI |  |  |  |  |
| 1665 |  | SVKEFKEEI |  |  |  |  |
| 1666 |  | SVKKQFEEL |  |  |  |  |
| 1667 |  | SVKKQFEEL |  |  |  |  |
| 1668 |  | SVKKQFEEL |  |  |  |  |
| 1669 |  | SVKYFLDNL |  |  |  |  |
| 1670 |  | SVLSKLSQL |  |  |  |  |
| 1671 |  | SVLSKLSQL |  |  |  |  |
| 1672 |  | SVNASSERL |  |  |  |  |
| 1673 |  | SVNASSERL |  |  |  |  |
| 1674 |  | SVNRLNYYV |  |  |  |  |
| 1675 |  | SVNRLNYYV |  |  |  |  |
| 1676 |  | SVNRLNYYV |  |  |  |  |
| 1677 |  | SVQDQWKEL |  |  |  |  |
| 1678 |  | SVQDQWKEL |  |  |  |  |
| 1679 |  | SVQDQWKEL |  |  |  |  |
| 1680 |  | SVQDQWKEL |  |  |  |  |
| 1681 |  | SVQRSFTQV |  |  |  |  |
| 1682 |  | SVQRSFTQV |  |  |  |  |
| 1683 |  | SVQRSFTQV |  |  |  |  |
| 1684 |  | SVSDQFYRY |  |  |  |  |
| 1685 |  | SVSDQFYRY |  |  |  |  |
| 1686 |  | SVSDQFYRY |  |  |  |  |
| 1687 |  | SVSNFLHSL |  |  |  |  |
| 1688 |  | SVSQINHKL |  |  |  |  |
| 1689 |  | SVSSHFVGF |  |  |  |  |
| 1690 |  | SVSSHFVGF |  |  |  |  |
| 1691 |  | SVSSHFVGF |  |  |  |  |
| 1692 |  | SVSSHFVGF |  |  |  |  |
| 1693 |  | SVSSIQNQM |  |  |  |  |
| 1694 |  | SVSTQLSLV |  |  |  |  |
| 1695 |  | SVSYLFSHV |  |  |  |  |
| 1696 |  | SVYEFFKYL |  |  |  |  |
| 1697 |  | SVYEFFKYL |  |  |  |  |
| 1698 |  | TANDGFVRL |  |  |  |  |
| 1699 |  | TANDGFVRL |  |  |  |  |
| 1700 |  | TANDGFVRL |  |  |  |  |
| 1701 |  | TANDGFVRL |  |  |  |  |
| 1702 |  | TIAEGFVKA |  |  |  |  |
| 1703 |  | TIAEGFVKA |  |  |  |  |
| 1704 |  | TIAEGFVKA |  |  |  |  |
| 1705 |  | TIAQLVHAV |  |  |  |  |
| 1706 |  | TIAQLVHAV |  |  |  |  |
| 1707 |  | TIAQLVHAV |  |  |  |  |
| 1708 |  | TIAQLVHAV |  |  |  |  |
| 1709 |  | TIAQVLVHL |  |  |  |  |
| 1710 |  | TIFEGFAKI |  |  |  |  |
| 1711 |  | TIFKDHVSL |  |  |  |  |
| 1712 |  | TIFKDHVSL |  |  |  |  |
| 1713 |  | TIFKDHVSL |  |  |  |  |
| 1714 |  | TIFKDHVSL |  |  |  |  |
| 1715 |  | TIITNFERL |  |  |  |  |
| 1716 |  | TIITNFERL |  |  |  |  |
| 1717 |  | TIITNFERL |  |  |  |  |
| 1718 |  | TIKEIHEYL |  |  |  |  |
| 1719 |  | TILRIDDLI |  |  |  |  |
| 1720 |  | TIMPKDIQL |  |  |  |  |
| 1721 |  | TINELRVNL |  |  |  |  |
| 1722 |  | TINELRVNL |  |  |  |  |
| 1723 |  | TINFIISQV |  |  |  |  |
| 1724 |  | TINFIISQV |  |  |  |  |
| 1725 |  | TINFIISQV |  |  |  |  |
| 1726 |  | TINGHNAEV |  |  |  |  |
| 1727 |  | TINGHNAEV |  |  |  |  |
| 1728 |  | TINGHNAEV |  |  |  |  |
| 1729 |  | TINIVFDRV |  |  |  |  |
| 1730 |  | TINIVFDRV |  |  |  |  |
| 1731 |  | TINKDTTHL |  |  |  |  |
| 1732 |  | TINRIQDLL |  |  |  |  |
| 1733 |  | TINRIQDLL |  |  |  |  |
| 1734 |  | TINRIQDLL |  |  |  |  |
| 1735 |  | TINRIQDLL |  |  |  |  |
| 1736 |  | TINSSMQAV |  |  |  |  |
| 1737 |  | TINVGLTSI |  |  |  |  |
| 1738 |  | TINVGLTSI |  |  |  |  |
| 1739 |  | TIQEEFYRV |  |  |  |  |
| 1740 |  | TIQEEFYRV |  |  |  |  |
| 1741 |  | TIQEEFYRV |  |  |  |  |
| 1742 |  | TIQEEFYRV |  |  |  |  |
| 1743 |  | TIRSITEIL |  |  |  |  |
| 1744 |  | TISKMNDFM |  |  |  |  |
| 1745 |  | TISSLFSRL |  |  |  |  |
| 1746 |  | TISSLFSRL |  |  |  |  |
| 1747 |  | TISSLFSRL |  |  |  |  |
| 1748 |  | TITDIISAL |  |  |  |  |
| 1749 |  | TIVDILERF |  |  |  |  |
| 1750 |  | TIYKDFVYI |  |  |  |  |
| 1751 |  | TIYKDFVYI |  |  |  |  |
| 1752 |  | TIYKDFVYI |  |  |  |  |
| 1753 |  | TLADIIARL |  |  |  |  |
| 1754 |  | TLADLLALR |  |  |  |  |
| 1755 |  | TLAKYLMEL |  |  |  |  |
| 1756 |  | TLFKGFFEL |  |  |  |  |
| 1757 |  | TLFKGFFEL |  |  |  |  |
| 1758 |  | TLIEDILGV |  |  |  |  |
| 1759 |  | TLNEKLTAL |  |  |  |  |
| 1760 |  | TLNEKLTAL |  |  |  |  |
| 1761 |  | TLNEKLTAL |  |  |  |  |
| 1762 |  | TLNEKLTAL |  |  |  |  |
| 1763 |  | TLNKFTEEL |  |  |  |  |
| 1764 |  | TLNSFIHVL |  |  |  |  |
| 1765 |  | TLQEFLERI |  |  |  |  |
| 1766 |  | TLQEFLERI |  |  |  |  |
| 1767 |  | TPAVVSERL |  |  |  |  |
| 1768 |  | TPAVVSERL |  |  |  |  |
| 1769 |  | TPHDFIEHI |  |  |  |  |
| 1770 |  | TPSEPHPVL |  |  |  |  |
| 1771 |  | TVADHIQKV |  |  |  |  |
| 1772 |  | TVADKIHSV |  |  |  |  |
| 1773 |  | TVADKIHSV |  |  |  |  |
| 1774 |  | TVADKIHSV |  |  |  |  |
| 1775 |  | TVAQIKAHV |  |  |  |  |
| 1776 |  | TVAQIKAHV |  |  |  |  |
| 1777 |  | TVAQIKAHV |  |  |  |  |
| 1778 |  | TVAQIKATV |  |  |  |  |
| 1779 |  | TVAQIKATV |  |  |  |  |
| 1780 |  | TVAQIKATV |  |  |  |  |
| 1781 |  | TVATLSERV |  |  |  |  |
| 1782 |  | TVATLSERV |  |  |  |  |
| 1783 |  | TVATLSERV |  |  |  |  |
| 1784 |  | TVFNFQEKV |  |  |  |  |
| 1785 |  | TVFNFQEKV |  |  |  |  |
| 1786 |  | TVFNFQEKV |  |  |  |  |
| 1787 |  | TVFNFQEKV |  |  |  |  |
| 1788 |  | TVFSTQDFL |  |  |  |  |
| 1789 |  | TVHDKFLAL |  |  |  |  |
| 1790 |  | TVHDKFLAL |  |  |  |  |
| 1791 |  | TVIDYFHQL |  |  |  |  |
| 1792 |  | TVIDYFHQL |  |  |  |  |
| 1793 |  | TVIDYFHQL |  |  |  |  |
| 1794 |  | TVIKLIERL |  |  |  |  |
| 1795 |  | TVIQFLERI |  |  |  |  |
| 1796 |  | TVIQFLERI |  |  |  |  |
| 1797 |  | TVIQFLERI |  |  |  |  |
| 1798 |  | TVIRPFPGL |  |  |  |  |
| 1799 |  | TVISNWIKY |  |  |  |  |
| 1800 |  | TVISNWIKY |  |  |  |  |
| 1801 |  | TVISNWIKY |  |  |  |  |
| 1802 |  | TVISNWIKY |  |  |  |  |
| 1803 |  | TVKAIIERL |  |  |  |  |
| 1804 |  | TVNQLAHAL |  |  |  |  |
| 1805 |  | TVNQLAHAL |  |  |  |  |
| 1806 |  | TVNSAREEL |  |  |  |  |
| 1807 |  | TVNSAREEL |  |  |  |  |
| 1808 |  | TVNSAREEL |  |  |  |  |
| 1809 |  | TVNSVEEKI |  |  |  |  |
| 1810 |  | TVNSVEEKI |  |  |  |  |
| 1811 |  | TVSPLFQKL |  |  |  |  |
| 1812 |  | TVSPLFQKL |  |  |  |  |
| 1813 |  | TVSPLFQKL |  |  |  |  |
| 1814 |  | VAADTLQRL |  |  |  |  |
| 1815 |  | VAADTLQRL |  |  |  |  |
| 1816 |  | VAKQGFDFL |  |  |  |  |
| 1817 |  | VAKQGFDFL |  |  |  |  |
| 1818 |  | VAKQGFDFL |  |  |  |  |
| 1819 |  | VAKQGFDFL |  |  |  |  |
| 1820 |  | VAKTWTELL |  |  |  |  |
| 1821 |  | VAKTWTELL |  |  |  |  |
| 1822 |  | VFTQPEYRI |  |  |  |  |
| 1823 |  | VFTQPEYRI |  |  |  |  |
| 1824 |  | VGGEALGRL |  |  |  |  |
| 1825 |  | VGLFEDTNL |  |  |  |  |
| 1826 |  | VGLFEDTNL |  |  |  |  |
| 1827 |  | VGMGQKDSY |  |  |  |  |
| 1828 |  | VHLTPEEKS |  |  |  |  |
| 1829 |  | VIAEILRGV |  |  |  |  |
| 1830 |  | VIAEILRGV |  |  |  |  |
| 1831 |  | VIAEILRGV |  |  |  |  |
| 1832 |  | VIAEILRGV |  |  |  |  |
| 1833 |  | VIAEQIQHL |  |  |  |  |
| 1834 |  | VIAEQIQHL |  |  |  |  |
| 1835 |  | VIAEQIQHL |  |  |  |  |
| 1836 |  | VIAEQLAKL |  |  |  |  |
| 1837 |  | VIAHDFLAF |  |  |  |  |
| 1838 |  | VIAHDFLAF |  |  |  |  |
| 1839 |  | VIAPIISNF |  |  |  |  |
| 1840 |  | VIAPIISNF |  |  |  |  |
| 1841 |  | VIAPIISNF |  |  |  |  |
| 1842 |  | VIAPIISNF |  |  |  |  |
| 1843 |  | VIAPNPAQL |  |  |  |  |
| 1844 |  | VIAPNPAQL |  |  |  |  |
| 1845 |  | VIAPNPAQL |  |  |  |  |
| 1846 |  | VIAPVGESL |  |  |  |  |
| 1847 |  | VIAQGIGKL |  |  |  |  |
| 1848 |  | VIAQGIGKL |  |  |  |  |
| 1849 |  | VIAQGIGKL |  |  |  |  |
| 1850 |  | VIAQGIGKL |  |  |  |  |
| 1851 |  | VIARAISKL |  |  |  |  |
| 1852 |  | VIARAISKL |  |  |  |  |
| 1853 |  | VIFDEIHYM |  |  |  |  |
| 1854 |  | VIFDEIHYM |  |  |  |  |
| 1855 |  | VIFDEVHYI |  |  |  |  |
| 1856 |  | VIFDEVHYI |  |  |  |  |
| 1857 |  | VIFTKFDEV |  |  |  |  |
| 1858 |  | VIFTKFDEV |  |  |  |  |
| 1859 |  | VIFTKFDEV |  |  |  |  |
| 1860 |  | VIFTKFDEV |  |  |  |  |
| 1861 |  | VIFVLQERI |  |  |  |  |
| 1862 |  | VIGPKGNSL |  |  |  |  |
| 1863 |  | VIHDFLFSL |  |  |  |  |
| 1864 |  | VIHDFLFSL |  |  |  |  |
| 1865 |  | VIHEKQTAV |  |  |  |  |
| 1866 |  | VIHPESNNL |  |  |  |  |
| 1867 |  | VIIETISLL |  |  |  |  |
| 1868 |  | VIIETISLL |  |  |  |  |
| 1869 |  | VIIKYLESL |  |  |  |  |
| 1870 |  | VIIKYLESL |  |  |  |  |
| 1871 |  | VIIKYLESL |  |  |  |  |
| 1872 |  | VIISVITQL |  |  |  |  |
| 1873 |  | VIKADFEQY |  |  |  |  |
| 1874 |  | VIKADFEQY |  |  |  |  |
| 1875 |  | VIKADFEQY |  |  |  |  |
| 1876 |  | VIKAIADTL |  |  |  |  |
| 1877 |  | VIKAIADTL |  |  |  |  |
| 1878 |  | VIKAIADTL |  |  |  |  |
| 1879 |  | VIKALLEVV |  |  |  |  |
| 1880 |  | VIKALLEVV |  |  |  |  |
| 1881 |  | VIKALLEVV |  |  |  |  |
| 1882 |  | VIKEDVSEL |  |  |  |  |
| 1883 |  | VIKEDVSEL |  |  |  |  |
| 1884 |  | VIKEGEEQL |  |  |  |  |
| 1885 |  | VIKEGEEQL |  |  |  |  |
| 1886 |  | VIKEGEEQL |  |  |  |  |
| 1887 |  | VIKEITQEM |  |  |  |  |
| 1888 |  | VIKGDFETI |  |  |  |  |
| 1889 |  | VIKGDFETI |  |  |  |  |
| 1890 |  | VIKGDFETI |  |  |  |  |
| 1891 |  | VIKGDFETI |  |  |  |  |
| 1892 |  | VILGIFNHL |  |  |  |  |
| 1893 |  | VILGIFNHL |  |  |  |  |
| 1894 |  | VILGIIHML |  |  |  |  |
| 1895 |  | VILGIKDNL |  |  |  |  |
| 1896 |  | VILGIKDNL |  |  |  |  |
| 1897 |  | VILGIKDNL |  |  |  |  |
| 1898 |  | VILGIKDNL |  |  |  |  |
| 1899 |  | VINELIGNL |  |  |  |  |
| 1900 |  | VINELIGNL |  |  |  |  |
| 1901 |  | VINELIGNL |  |  |  |  |
| 1902 |  | VINELIGNL |  |  |  |  |
| 1903 |  | VINENFGTL |  |  |  |  |
| 1904 |  | VINFDFPKL |  |  |  |  |
| 1905 |  | VINFDFPKL |  |  |  |  |
| 1906 |  | VINFDFPKL |  |  |  |  |
| 1907 |  | VINFDFPKL |  |  |  |  |
| 1908 |  | VINFTAEKL |  |  |  |  |
| 1909 |  | VINGNIKTV |  |  |  |  |
| 1910 |  | VINGNIKTV |  |  |  |  |
| 1911 |  | VINGNIKTV |  |  |  |  |
| 1912 |  | VINGNIKTV |  |  |  |  |
| 1913 |  | VINPSWSEL |  |  |  |  |
| 1914 |  | VINPSWSEL |  |  |  |  |
| 1915 |  | VINQILTEM |  |  |  |  |
| 1916 |  | VINQILTEM |  |  |  |  |
| 1917 |  | VINQILTEM |  |  |  |  |
| 1918 |  | VINTLTELF |  |  |  |  |
| 1919 |  | VINTLTELF |  |  |  |  |
| 1920 |  | VINTLTELF |  |  |  |  |
| 1921 |  | VIQALGEHL |  |  |  |  |
| 1922 |  | VIQALGEHL |  |  |  |  |
| 1923 |  | VIQALGEHL |  |  |  |  |
| 1924 |  | VIQGLVERL |  |  |  |  |
| 1925 |  | VIQGLVERL |  |  |  |  |
| 1926 |  | VIQHVFQNL |  |  |  |  |
| 1927 |  | VIQHVFQNL |  |  |  |  |
| 1928 |  | VIQHVFQNL |  |  |  |  |
| 1929 |  | VIQKIGDLL |  |  |  |  |
| 1930 |  | VISDLQSKL |  |  |  |  |
| 1931 |  | VISDLQSKL |  |  |  |  |
| 1932 |  | VISDLQSKL |  |  |  |  |
| 1933 |  | VISEGFLKA |  |  |  |  |
| 1934 |  | VISEGFLKA |  |  |  |  |
| 1935 |  | VISEGFLKA |  |  |  |  |
| 1936 |  | VISEGFLKA |  |  |  |  |
| 1937 |  | VISELREKL |  |  |  |  |
| 1938 |  | VISELREKL |  |  |  |  |
| 1939 |  | VISELREKL |  |  |  |  |
| 1940 |  | VISGHFDGV |  |  |  |  |
| 1941 |  | VISGHFDGV |  |  |  |  |
| 1942 |  | VISGINEKL |  |  |  |  |
| 1943 |  | VISGINEKL |  |  |  |  |
| 1944 |  | VISGINEKL |  |  |  |  |
| 1945 |  | VISGINEKL |  |  |  |  |
| 1946 |  | VISLEGKPL |  |  |  |  |
| 1947 |  | VISPLNQLL |  |  |  |  |
| 1948 |  | VISRFLEEY |  |  |  |  |
| 1949 |  | VISRFLEEY |  |  |  |  |
| 1950 |  | VISSIRNFL |  |  |  |  |
| 1951 |  | VISSIRNFL |  |  |  |  |
| 1952 |  | VISSIRNFL |  |  |  |  |
| 1953 |  | VISSIRNFL |  |  |  |  |
| 1954 |  | VISSVAHYL |  |  |  |  |
| 1955 |  | VISSVAHYL |  |  |  |  |
| 1956 |  | VISSVAHYL |  |  |  |  |
| 1957 |  | VIVTAHAFV |  |  |  |  |
| 1958 |  | VIVTAHAFV |  |  |  |  |
| 1959 |  | VIYPFMQGL |  |  |  |  |
| 1960 |  | VIYPFMQGL |  |  |  |  |
| 1961 |  | VIYPFMQGL |  |  |  |  |
| 1962 |  | VLAGHDNRV |  |  |  |  |
| 1963 |  | VLAPMFERL |  |  |  |  |
| 1964 |  | VLAPMFERL |  |  |  |  |
| 1965 |  | VLAPMFERL |  |  |  |  |
| 1966 |  | VLAPMFERL |  |  |  |  |
| 1967 |  | VLAWGLLNV |  |  |  |  |
| 1968 |  | VLGPPFTHL |  |  |  |  |
| 1969 |  | VLHPYPTQI |  |  |  |  |
| 1970 |  | VLHPYPTQI |  |  |  |  |
| 1971 |  | VLKDLMHGL |  |  |  |  |
| 1972 |  | VLKEAWTEL |  |  |  |  |
| 1973 |  | VLKEIVERV |  |  |  |  |
| 1974 |  | VLKEIVERV |  |  |  |  |
| 1975 |  | VLKEIVERV |  |  |  |  |
| 1976 |  | VLKIGDFGL |  |  |  |  |
| 1977 |  | VLKNFTDQL |  |  |  |  |
| 1978 |  | VLKTGFLFV |  |  |  |  |
| 1979 |  | VLKTGFLFV |  |  |  |  |
| 1980 |  | VLKTGFLFV |  |  |  |  |
| 1981 |  | VLKTGFLFV |  |  |  |  |
| 1982 |  | VLKVFLENV |  |  |  |  |
| 1983 |  | VLKVFLENV |  |  |  |  |
| 1984 |  | VLKVFLENV |  |  |  |  |
| 1985 |  | VLKVFLENV |  |  |  |  |
| 1986 |  | VLMKALEYL |  |  |  |  |
| 1987 |  | VLMKFMEML |  |  |  |  |
| 1988 |  | VLMKFMEML |  |  |  |  |
| 1989 |  | VLNEYFHNV |  |  |  |  |
| 1990 |  | VLNEYFHNV |  |  |  |  |
| 1991 |  | VLNEYFHNV |  |  |  |  |
| 1992 |  | VLNGKFANL |  |  |  |  |
| 1993 |  | VLNGKFANL |  |  |  |  |
| 1994 |  | VLNGKFANL |  |  |  |  |
| 1995 |  | VLNGKFANL |  |  |  |  |
| 1996 |  | VLPEFLRLP |  |  |  |  |
| 1997 |  | VLPEFLRLP |  |  |  |  |
| 1998 |  | VLPEFLRLP |  |  |  |  |
| 1999 |  | VLPEFLRLP |  |  |  |  |
| 2000 |  | VLQDTVEQL |  |  |  |  |
| 2001 |  | VLQDTVEQL |  |  |  |  |
| 2002 |  | VLQEDAEKL |  |  |  |  |
| 2003 |  | VLQEDAEKL |  |  |  |  |
| 2004 |  | VLQEDAEKL |  |  |  |  |
| 2005 |  | VLSDIIQNL |  |  |  |  |
| 2006 |  | VLSDIIQNL |  |  |  |  |
| 2007 |  | VLSDIIQNL |  |  |  |  |
| 2008 |  | VLYDWKERL |  |  |  |  |
| 2009 |  | VLYDWKERL |  |  |  |  |
| 2010 |  | VLYDWKERL |  |  |  |  |
| 2011 |  | VMAPRTLIL |  |  |  |  |
| 2012 |  | VMAPRTLIL |  |  |  |  |
| 2013 |  | VPISFVDRV |  |  |  |  |
| 2014 |  | VPISFVDRV |  |  |  |  |
| 2015 |  | VPISFVDRV |  |  |  |  |
| 2016 |  | VPNNKITVV |  |  |  |  |
| 2017 |  | VPNNKITVV |  |  |  |  |
| 2018 |  | VPNNKITVV |  |  |  |  |
| 2019 |  | VQTAELTKV |  |  |  |  |
| 2020 |  | VRLLLPGEL |  |  |  |  |
| 2021 |  | VSVGANPVQ |  |  |  |  |
| 2022 |  | VTFWKWISL |  |  |  |  |
| 2023 |  | VTIESVADY |  |  |  |  |
| 2024 |  | VTMEWWTHL |  |  |  |  |
| 2025 |  | VTMTASRML |  |  |  |  |
| 2026 |  | VTMTASRML |  |  |  |  |
| 2027 |  | VTNPAFTKL |  |  |  |  |
| 2028 |  | VTNPAFTKL |  |  |  |  |
| 2029 |  | VTNPAFTKL |  |  |  |  |
| 2030 |  | VTNPAFTKL |  |  |  |  |
| 2031 |  | VTSPIKMKI |  |  |  |  |
| 2032 |  | VVAAHLAGA |  |  |  |  |
| 2033 |  | VVADIQDRI |  |  |  |  |
| 2034 |  | VVADIQDRI |  |  |  |  |
| 2035 |  | VVADIQDRI |  |  |  |  |
| 2036 |  | VVADLFHSL |  |  |  |  |
| 2037 |  | VVADLFHSL |  |  |  |  |
| 2038 |  | VVADLFHSL |  |  |  |  |
| 2039 |  | VVAGAFVHF |  |  |  |  |
| 2040 |  | VVAGFGHFL |  |  |  |  |
| 2041 |  | VVAGFGHFL |  |  |  |  |
| 2042 |  | VVAGIKEYF |  |  |  |  |
| 2043 |  | VVAGIKEYF |  |  |  |  |
| 2044 |  | VVAGIKEYF |  |  |  |  |
| 2045 |  | VVAGIKEYF |  |  |  |  |
| 2046 |  | VVAGRFIEV |  |  |  |  |
| 2047 |  | VVAGRFIEV |  |  |  |  |
| 2048 |  | VVARFVEFL |  |  |  |  |
| 2049 |  | VVARFVEFL |  |  |  |  |
| 2050 |  | VVARFVEFL |  |  |  |  |
| 2051 |  | VVASVKEGV |  |  |  |  |
| 2052 |  | VVASVKEGV |  |  |  |  |
| 2053 |  | VVFGEHTLL |  |  |  |  |
| 2054 |  | VVFGEHTLL |  |  |  |  |
| 2055 |  | VVFGEHTLL |  |  |  |  |
| 2056 |  | VVFRLQEFI |  |  |  |  |
| 2057 |  | VVGHITEGV |  |  |  |  |
| 2058 |  | VVGKFVEFF |  |  |  |  |
| 2059 |  | VVGKFVEFF |  |  |  |  |
| 2060 |  | VVGKFVEFF |  |  |  |  |
| 2061 |  | VVHDGFEGL |  |  |  |  |
| 2062 |  | VVHDGFEGL |  |  |  |  |
| 2063 |  | VVHDGFEGL |  |  |  |  |
| 2064 |  | VVHDGFEGL |  |  |  |  |
| 2065 |  | VVHKIIELL |  |  |  |  |
| 2066 |  | VVHKIIELL |  |  |  |  |
| 2067 |  | VVHKIIELL |  |  |  |  |
| 2068 |  | VVINYTEQL |  |  |  |  |
| 2069 |  | VVINYTEQL |  |  |  |  |
| 2070 |  | VVINYTEQL |  |  |  |  |
| 2071 |  | VVIQENGSL |  |  |  |  |
| 2072 |  | VVIQENGSL |  |  |  |  |
| 2073 |  | VVITDPEKL |  |  |  |  |
| 2074 |  | VVKGQVEYL |  |  |  |  |
| 2075 |  | VVKGQVEYL |  |  |  |  |
| 2076 |  | VVKGQVEYL |  |  |  |  |
| 2077 |  | VVKKHIKEL |  |  |  |  |
| 2078 |  | VVKLWRENL |  |  |  |  |
| 2079 |  | VVNDFWAEI |  |  |  |  |
| 2080 |  | VVNDGVVRL |  |  |  |  |
| 2081 |  | VVNDGVVRL |  |  |  |  |
| 2082 |  | VVNDGVVRL |  |  |  |  |
| 2083 |  | VVNETVEKL |  |  |  |  |
| 2084 |  | VVNETVEKL |  |  |  |  |
| 2085 |  | VVNETVEKL |  |  |  |  |
| 2086 |  | VVNGKVEYF |  |  |  |  |
| 2087 |  | VVNKLFEFM |  |  |  |  |
| 2088 |  | VVNKLIQFL |  |  |  |  |
| 2089 |  | VVNRAQHQL |  |  |  |  |
| 2090 |  | VVNRAQHQL |  |  |  |  |
| 2091 |  | VVNRAQHQL |  |  |  |  |
| 2092 |  | VVNRLTEEL |  |  |  |  |
| 2093 |  | VVNRLTEEL |  |  |  |  |
| 2094 |  | VVNRLTEEL |  |  |  |  |
| 2095 |  | VVNRLTTAV |  |  |  |  |
| 2096 |  | VVNSALYNV |  |  |  |  |
| 2097 |  | VVNVLKSLL |  |  |  |  |
| 2098 |  | VVNVWTHLL |  |  |  |  |
| 2099 |  | VVNVWTHLL |  |  |  |  |
| 2100 |  | VVQKFFESL |  |  |  |  |
| 2101 |  | VVQKFFESL |  |  |  |  |
| 2102 |  | VVQPYNSLL |  |  |  |  |
| 2103 |  | VVRHQLLKT |  |  |  |  |
| 2104 |  | VVSRFDEQL |  |  |  |  |
| 2105 |  | VVSRFDEQL |  |  |  |  |
| 2106 |  | VVSRFDEQL |  |  |  |  |
| 2107 |  | VVSSVVHPL |  |  |  |  |
| 2108 |  | VVVDPIQSV |  |  |  |  |
| 2109 |  | VVYPKPEQL |  |  |  |  |
| 2110 |  | VVYPKPEQL |  |  |  |  |
| 2111 |  | VVYPKPEQL |  |  |  |  |
| 2112 |  | VVYPWTQRF |  |  |  |  |
| 2113 |  | VVYPWTQRF |  |  |  |  |
| 2114 |  | VVYTPWSNL |  |  |  |  |
| 2115 |  | VVYTPWSNL |  |  |  |  |
| 2116 |  | WIANNLFGL |  |  |  |  |
| 2117 |  | WIANNLFGL |  |  |  |  |
| 2118 |  | WIANNLFGL |  |  |  |  |
| 2119 |  | WIANNLFGL |  |  |  |  |
| 2120 |  | WIKPGMTMI |  |  |  |  |
| 2121 |  | WINEKEAAL |  |  |  |  |
| 2122 |  | WINEKMTLV |  |  |  |  |
| 2123 |  | WIQAEWKEI |  |  |  |  |
| 2124 |  | WLAEKLPTL |  |  |  |  |
| 2125 |  | WLAEKLPTL |  |  |  |  |
| 2126 |  | WLAEKLPTL |  |  |  |  |
| 2127 |  | WLAETTHAL |  |  |  |  |
| 2128 |  | WLIGQWISV |  |  |  |  |
| 2129 |  | WLKEGVLGL |  |  |  |  |
| 2130 |  | WLQEHVADL |  |  |  |  |
| 2131 |  | WLQEKLSFF |  |  |  |  |
| 2132 |  | WLVDHVYAI |  |  |  |  |
| 2133 |  | WLVDHVYAI |  |  |  |  |
| 2134 |  | WLVDHVYAI |  |  |  |  |
| 2135 |  | WPGYDLNLF |  |  |  |  |
| 2136 |  | WQYSTFSGF |  |  |  |  |
| 2137 |  | WTQRFFDSF |  |  |  |  |
| 2138 |  | WVAERVELL |  |  |  |  |
| 2139 |  | WVAERVELL |  |  |  |  |
| 2140 |  | WVEDLFEKF |  |  |  |  |
| 2141 |  | WVFEHPETL |  |  |  |  |
| 2142 |  | WVFEHPETL |  |  |  |  |
| 2143 |  | WVFEHPETL |  |  |  |  |
| 2144 |  | WVFGTNVMV |  |  |  |  |
| 2145 |  | WVFGTNVMV |  |  |  |  |
| 2146 |  | WVGILSHGL |  |  |  |  |
| 2147 |  | WVGQQVHNL |  |  |  |  |
| 2148 |  | WVKEKVVAL |  |  |  |  |
| 2149 |  | WVKEKVVAL |  |  |  |  |
| 2150 |  | WVKEKVVAL |  |  |  |  |
| 2151 |  | WVNEDRHYM |  |  |  |  |
| 2152 |  | WVNEDRHYM |  |  |  |  |
| 2153 |  | WVNEDRHYM |  |  |  |  |
| 2154 |  | WVQDKITQM |  |  |  |  |
| 2155 |  | WVQDKITQM |  |  |  |  |
| 2156 |  | WVQDKITQM |  |  |  |  |
| 2157 |  | WVQDKITQM |  |  |  |  |
| 2158 |  | WVQKKFQKY |  |  |  |  |
| 2159 |  | WVSDFSERI |  |  |  |  |
| 2160 |  | WVSGKFSSL |  |  |  |  |
| 2161 |  | WVSGKFSSL |  |  |  |  |
| 2162 |  | WVSGKFSSL |  |  |  |  |
| 2163 |  | WVTEIFSQI |  |  |  |  |
| 2164 |  | WVTEIFSQI |  |  |  |  |
| 2165 |  | WVTEIFSQI |  |  |  |  |
| 2166 |  | WVYPFLEHI |  |  |  |  |
| 2167 |  | WVYPFLEHI |  |  |  |  |
| 2168 |  | YAAELIERV |  |  |  |  |
| 2169 |  | YAAELIERV |  |  |  |  |
| 2170 |  | YAIKNIHGV |  |  |  |  |
| 2171 |  | YASDYFDQL |  |  |  |  |
| 2172 |  | YCAEIAHNV |  |  |  |  |
| 2173 |  | YCAEIAHNV |  |  |  |  |
| 2174 |  | YIAAFLSHF |  |  |  |  |
| 2175 |  | YIAAFLSHF |  |  |  |  |
| 2176 |  | YIAAFLSHF |  |  |  |  |
| 2177 |  | YIAEEYSRL |  |  |  |  |
| 2178 |  | YIAEEYSRL |  |  |  |  |
| 2179 |  | YIAEEYSRL |  |  |  |  |
| 2180 |  | YIAKDLMKV |  |  |  |  |
| 2181 |  | YIAKDLMKV |  |  |  |  |
| 2182 |  | YIAPLFDKF |  |  |  |  |
| 2183 |  | YIAPLFDKF |  |  |  |  |
| 2184 |  | YIAPLFDKF |  |  |  |  |
| 2185 |  | YIAQNFKQL |  |  |  |  |
| 2186 |  | YIAQNFKQL |  |  |  |  |
| 2187 |  | YIATIHSRL |  |  |  |  |
| 2188 |  | YIDDVFHAL |  |  |  |  |
| 2189 |  | YIEEAIEKL |  |  |  |  |
| 2190 |  | YIEKFTDFL |  |  |  |  |
| 2191 |  | YIEKFTDFL |  |  |  |  |
| 2192 |  | YIEKFTDFL |  |  |  |  |
| 2193 |  | YIFAHVGQL |  |  |  |  |
| 2194 |  | YIFAHVGQL |  |  |  |  |
| 2195 |  | YIFAHVGQL |  |  |  |  |
| 2196 |  | YIFSVVENL |  |  |  |  |
| 2197 |  | YIFSVVENL |  |  |  |  |
| 2198 |  | YIFSVVENL |  |  |  |  |
| 2199 |  | YIFSVVENL |  |  |  |  |
| 2200 |  | YIHALDNGL |  |  |  |  |
| 2201 |  | YIHVHFLEV |  |  |  |  |
| 2202 |  | YIIRIYQEL |  |  |  |  |
| 2203 |  | YIIRIYQEL |  |  |  |  |
| 2204 |  | YIIRIYQEL |  |  |  |  |
| 2205 |  | YIITLHELL |  |  |  |  |
| 2206 |  | YIKAAVKNV |  |  |  |  |
| 2207 |  | YIKDEQKNL |  |  |  |  |
| 2208 |  | YIKDFFEQM |  |  |  |  |
| 2209 |  | YIKDFFEQM |  |  |  |  |
| 2210 |  | YIKDFFEQM |  |  |  |  |
| 2211 |  | YIKDGITRV |  |  |  |  |
| 2212 |  | YIKDGITRV |  |  |  |  |
| 2213 |  | YIKDYMKSI |  |  |  |  |
| 2214 |  | YIKFISETL |  |  |  |  |
| 2215 |  | YIKFISETL |  |  |  |  |
| 2216 |  | YIKFISETL |  |  |  |  |
| 2217 |  | YIKFISETL |  |  |  |  |
| 2218 |  | YIKRLLETL |  |  |  |  |
| 2219 |  | YIKRLLETL |  |  |  |  |
| 2220 |  | YILGKFFAL |  |  |  |  |
| 2221 |  | YILGKFFAL |  |  |  |  |
| 2222 |  | YIMGYISKV |  |  |  |  |
| 2223 |  | YIMGYISKV |  |  |  |  |
| 2224 |  | YIMGYISKV |  |  |  |  |
| 2225 |  | YINAYSHTM |  |  |  |  |
| 2226 |  | YINENWDRL |  |  |  |  |
| 2227 |  | YINENWDRL |  |  |  |  |
| 2228 |  | YINERNAKF |  |  |  |  |
| 2229 |  | YINERNAKF |  |  |  |  |
| 2230 |  | YINERNAKF |  |  |  |  |
| 2231 |  | YINKISSTL |  |  |  |  |
| 2232 |  | YINKISSTL |  |  |  |  |
| 2233 |  | YINKISSTL |  |  |  |  |
| 2234 |  | YINKISSTL |  |  |  |  |
| 2235 |  | YIQQIVQRL |  |  |  |  |
| 2236 |  | YIQRLLHAL |  |  |  |  |
| 2237 |  | YIQRTFEEF |  |  |  |  |
| 2238 |  | YIQSKFEDL |  |  |  |  |
| 2239 |  | YIQSKFEDL |  |  |  |  |
| 2240 |  | YIQSKFEDL |  |  |  |  |
| 2241 |  | YIRTGFINL |  |  |  |  |
| 2242 |  | YIRTGFINL |  |  |  |  |
| 2243 |  | YIRTGFINL |  |  |  |  |
| 2244 |  | YIRTGFINL |  |  |  |  |
| 2245 |  | YISNATHML |  |  |  |  |
| 2246 |  | YISNATHML |  |  |  |  |
| 2247 |  | YISPYFINT |  |  |  |  |
| 2248 |  | YISPYFINT |  |  |  |  |
| 2249 |  | YISTEFNRY |  |  |  |  |
| 2250 |  | YITDLFQVL |  |  |  |  |
| 2251 |  | YITDLFQVL |  |  |  |  |
| 2252 |  | YITDLFQVL |  |  |  |  |
| 2253 |  | YITLFIEKL |  |  |  |  |
| 2254 |  | YITLFIEKL |  |  |  |  |
| 2255 |  | YITLFIEKL |  |  |  |  |
| 2256 |  | YITRYIASL |  |  |  |  |
| 2257 |  | YIVDLLTHL |  |  |  |  |
| 2258 |  | YIVDLLTHL |  |  |  |  |
| 2259 |  | YIVDLLTHL |  |  |  |  |
| 2260 |  | YIVDLLTHL |  |  |  |  |
| 2261 |  | YIVKWPLSL |  |  |  |  |
| 2262 |  | YIVKWPLSL |  |  |  |  |
| 2263 |  | YIYFQFQNL |  |  |  |  |
| 2264 |  | YIYFQFQNL |  |  |  |  |
| 2265 |  | YIYFQFQNL |  |  |  |  |
| 2266 |  | YIYNREEYA |  |  |  |  |
| 2267 |  | YIYNREEYA |  |  |  |  |
| 2268 |  | YKAQPVIQF |  |  |  |  |
| 2269 |  | YLADIFTKL |  |  |  |  |
| 2270 |  | YLADIFTKL |  |  |  |  |
| 2271 |  | YLADIFTKL |  |  |  |  |
| 2272 |  | YLADIFTKL |  |  |  |  |
| 2273 |  | YLADLYHFV |  |  |  |  |
| 2274 |  | YLADLYHFV |  |  |  |  |
| 2275 |  | YLAHFIEGL |  |  |  |  |
| 2276 |  | YLAKVKSLL |  |  |  |  |
| 2277 |  | YLAKVKSLL |  |  |  |  |
| 2278 |  | YLANGQTKV |  |  |  |  |
| 2279 |  | YLARFLEGL |  |  |  |  |
| 2280 |  | YLATLTEKM |  |  |  |  |
| 2281 |  | YLATLTEKM |  |  |  |  |
| 2282 |  | YLATLTEKM |  |  |  |  |
| 2283 |  | YLATVRSDL |  |  |  |  |
| 2284 |  | YLFERIKEL |  |  |  |  |
| 2285 |  | YLFERIKEL |  |  |  |  |
| 2286 |  | YLGDVSERV |  |  |  |  |
| 2287 |  | YLGRLAHEV |  |  |  |  |
| 2288 |  | YLGRLAHEV |  |  |  |  |
| 2289 |  | YLGRLAHEV |  |  |  |  |
| 2290 |  | YLGRLAHEV |  |  |  |  |
| 2291 |  | YLHEFRDKL |  |  |  |  |
| 2292 |  | YLHEFRDKL |  |  |  |  |
| 2293 |  | YLHEFRDKL |  |  |  |  |
| 2294 |  | YLHEFRDKL |  |  |  |  |
| 2295 |  | YLIFNEYEI |  |  |  |  |
| 2296 |  | YLIFNEYEI |  |  |  |  |
| 2297 |  | YLIPLLERL |  |  |  |  |
| 2298 |  | YLIPLLERL |  |  |  |  |
| 2299 |  | YLITLLEHL |  |  |  |  |
| 2300 |  | YLKDLIEEV |  |  |  |  |
| 2301 |  | YLKDLIEEV |  |  |  |  |
| 2302 |  | YLKYFQDQV |  |  |  |  |
| 2303 |  | YLNALVHLI |  |  |  |  |
| 2304 |  | YLNDGLWHM |  |  |  |  |
| 2305 |  | YLNDLHEVL |  |  |  |  |
| 2306 |  | YLNDLHEVL |  |  |  |  |
| 2307 |  | YLNEIKDSV |  |  |  |  |
| 2308 |  | YLNETFSEL |  |  |  |  |
| 2309 |  | YLSGIAHFL |  |  |  |  |
| 2310 |  | YLSGIAHFL |  |  |  |  |
| 2311 |  | YLSGIAHFL |  |  |  |  |
| 2312 |  | YLSGIAHFL |  |  |  |  |
| 2313 |  | YLVKIFEEL |  |  |  |  |
| 2314 |  | YLVKIFEEL |  |  |  |  |
| 2315 |  | YMAELIERL |  |  |  |  |
| 2316 |  | YMAELIERL |  |  |  |  |
| 2317 |  | YMAELIERL |  |  |  |  |
| 2318 |  | YMAELIERL |  |  |  |  |
| 2319 |  | YMIAHITGL |  |  |  |  |
| 2320 |  | YMIAHITGL |  |  |  |  |
| 2321 |  | YMIAHITGL |  |  |  |  |
| 2322 |  | YMIAHITGL |  |  |  |  |
| 2323 |  | YNTISNVLF |  |  |  |  |
| 2324 |  | YPASIVHQV |  |  |  |  |
| 2325 |  | YPASIVHQV |  |  |  |  |
| 2326 |  | YPASIVHQV |  |  |  |  |
| 2327 |  | YPNERFELL |  |  |  |  |
| 2328 |  | YPNERFELL |  |  |  |  |
| 2329 |  | YPNERFELL |  |  |  |  |
| 2330 |  | YPSETFTRV |  |  |  |  |
| 2331 |  | YPSETFTRV |  |  |  |  |
| 2332 |  | YPSETFTRV |  |  |  |  |
| 2333 |  | YPSETFTRV |  |  |  |  |
| 2334 |  | YPVEIHEYL |  |  |  |  |
| 2335 |  | YPVEIHEYL |  |  |  |  |
| 2336 |  | YPVEIHEYL |  |  |  |  |
| 2337 |  | YPVEIHEYL |  |  |  |  |
| 2338 |  | YQKVVAGVA |  |  |  |  |
| 2339 |  | YSFHKFHYL |  |  |  |  |
| 2340 |  | YSNPIKEEM |  |  |  |  |
| 2341 |  | YSNPIKEEM |  |  |  |  |
| 2342 |  | YSNPIKEEM |  |  |  |  |
| 2343 |  | YTAQPTQGY |  |  |  |  |
| 2344 |  | YTAQPTQGY |  |  |  |  |
| 2345 |  | YTKAWEEYY |  |  |  |  |
| 2346 |  | YTKAWEEYY |  |  |  |  |
| 2347 |  | YTKAWEEYY |  |  |  |  |
| 2348 |  | YTMVYWHAL |  |  |  |  |
| 2349 |  | YTMVYWHAL |  |  |  |  |
| 2350 |  | YTQPGPGYR |  |  |  |  |
| 2351 |  | YTQPGPGYR |  |  |  |  |
| 2352 |  | YTQQATQSY |  |  |  |  |
| 2353 |  | YTQQATQSY |  |  |  |  |
| 2354 |  | YTQTQFHNL |  |  |  |  |
| 2355 |  | YTQTQFHNL |  |  |  |  |
| 2356 |  | YTQTQFHNL |  |  |  |  |
| 2357 |  | YVAELIQQL |  |  |  |  |
| 2358 |  | YVAELIQQL |  |  |  |  |
| 2359 |  | YVAELIQQL |  |  |  |  |
| 2360 |  | YVAPHLNNL |  |  |  |  |
| 2361 |  | YVAPHLNNL |  |  |  |  |
| 2362 |  | YVAPHLNNL |  |  |  |  |
| 2363 |  | YVAQAGLEL |  |  |  |  |
| 2364 |  | YVASGSEQL |  |  |  |  |
| 2365 |  | YVASGSEQL |  |  |  |  |
| 2366 |  | YVASGSEQL |  |  |  |  |
| 2367 |  | YVASNQHEV |  |  |  |  |
| 2368 |  | YVASNQHEV |  |  |  |  |
| 2369 |  | YVASNQHEV |  |  |  |  |
| 2370 |  | YVASVHQDL |  |  |  |  |
| 2371 |  | YVFPGVTRL |  |  |  |  |
| 2372 |  | YVFPGVTRL |  |  |  |  |
| 2373 |  | YVFPGVTRL |  |  |  |  |
| 2374 |  | YVFPGVTRL |  |  |  |  |
| 2375 |  | YVHALVAYF |  |  |  |  |
| 2376 |  | YVHIMKEGL |  |  |  |  |
| 2377 |  | YVHIMKEGL |  |  |  |  |
| 2378 |  | YVHIMKEGL |  |  |  |  |
| 2379 |  | YVHIMKEGL |  |  |  |  |
| 2380 |  | YVHMVTHFI |  |  |  |  |
| 2381 |  | YVIDPIKGL |  |  |  |  |
| 2382 |  | YVIDPIKGL |  |  |  |  |
| 2383 |  | YVINDLTAV |  |  |  |  |
| 2384 |  | YVIQKFFEF |  |  |  |  |
| 2385 |  | YVIQKFFEF |  |  |  |  |
| 2386 |  | YVIQKFFEF |  |  |  |  |
| 2387 |  | YVITDLTQL |  |  |  |  |
| 2388 |  | YVITDLTQL |  |  |  |  |
| 2389 |  | YVITDLTQL |  |  |  |  |
| 2390 |  | YVKDIYAYL |  |  |  |  |
| 2391 |  | YVKDIYAYL |  |  |  |  |
| 2392 |  | YVKDIYAYL |  |  |  |  |
| 2393 |  | YVKDIYAYL |  |  |  |  |
| 2394 |  | YVKDIYQYL |  |  |  |  |
| 2395 |  | YVKDIYQYL |  |  |  |  |
| 2396 |  | YVKDIYQYL |  |  |  |  |
| 2397 |  | YVKDRVEKV |  |  |  |  |
| 2398 |  | YVKDRVEKV |  |  |  |  |
| 2399 |  | YVKKFGENF |  |  |  |  |
| 2400 |  | YVKKFGENF |  |  |  |  |
| 2401 |  | YVKKFGENF |  |  |  |  |
| 2402 |  | YVNAMNDKI |  |  |  |  |
| 2403 |  | YVNAMNDKI |  |  |  |  |
| 2404 |  | YVNAMNDKI |  |  |  |  |
| 2405 |  | YVNIIKILL |  |  |  |  |
| 2406 |  | YVNIIKILL |  |  |  |  |
| 2407 |  | YVNIIKILL |  |  |  |  |
| 2408 |  | YVNIIKILL |  |  |  |  |
| 2409 |  | YVNSIWDLL |  |  |  |  |
| 2410 |  | YVNTTEEKF |  |  |  |  |
| 2411 |  | YVNTTEEKF |  |  |  |  |
| 2412 |  | YVNTTEEKF |  |  |  |  |
| 2413 |  | YVQDFLNHL |  |  |  |  |
| 2414 |  | YVQDFLNHL |  |  |  |  |
| 2415 |  | YVQDFLNHL |  |  |  |  |
| 2416 |  | YVQDFLNHL |  |  |  |  |
| 2417 |  | YVSDHWAQY |  |  |  |  |
| 2418 |  | YVSEILEKV |  |  |  |  |
| 2419 |  | YVSKYINYI |  |  |  |  |
| 2420 |  | YVSKYINYI |  |  |  |  |
| 2421 |  | YVSKYINYI |  |  |  |  |
| 2422 |  | YVTPVNRNV |  |  |  |  |
| 2423 |  | YVTPVNRNV |  |  |  |  |
| 2424 |  | YVVADLDKL |  |  |  |  |
| 2425 |  | YVVPFVAKV |  |  |  |  |
| 2426 |  | YVYEFRDKL |  |  |  |  |
| 2427 |  | YVYEFRDKL |  |  |  |  |
| 2428 |  | YVYEFRDKL |  |  |  |  |
| 2429 |  | YVYQPMEKL |  |  |  |  |
| 2430 |  | YVYQPMEKL |  |  |  |  |
| 2431 |  | YVYQPMEKL |  |  |  |  |
| 2432 |  | YVYSHFLQF |  |  |  |  |
| 2433 |  | YVYSHFLQF |  |  |  |  |
| 2434 |  | YVYSHFLQF |  |  |  |  |
| 2435 |  | YVYYIFERL |  |  |  |  |
| 2436 |  | YVYYIFERL |  |  |  |  |
