# Supplementary material for: Characterization of the Canine MHC Class I DLA-88*50101 Peptide Binding Motif as a Prerequisite for Canine T Cell Immunotherapy
Source: PLoS One. 2016 Nov 28;11(11):e0167017. doi: 10.1371/journal.pone.0167017 (PMC5125661; doi:10.1371/journal.pone.0167017)
Supplement: S2 Table — (DOCX) [file pone.0167017.s003.docx]

| \| Position \| \| \| \| \| \| \| \| \| \| \| --- \| --- \| --- \| --- \| --- \| --- \| --- \| --- \| --- \| --- \| \| Residue \| 1 \| 2 \| 3 \| 4 \| 5 \| 6 \| 7 \| 8 \| 9 \| \| A \| 4.31 \| 2.02 \| 21.67 \| 4.85 \| 3.90 \| 2.56 \| 5.65 \| 6.33 \| 0.94 \| \| C \| 0.00 \| 0.00 \| 0.00 \| 0.13 \| 0.00 \| 0.00 \| 0.00 \| 0.00 \| 0.00 \| \| D \| 0.27 \| 0.13 \| 0.27 \| 15.48 \| 4.17 \| 0.81 \| 8.34 \| 0.67 \| 0.00 \| \| E \| 1.62 \| 0.67 \| 1.08 \| 17.63 \| 4.31 \| 3.77 \| 29.07 \| 10.36 \| 0.27 \| \| F \| 10.09 \| 0.13 \| 5.92 \| 0.94 \| 11.44 \| 23.96 \| 1.21 \| 5.52 \| 5.52 \| \| G \| 2.56 \| 0.40 \| 2.29 \| 6.73 \| 6.86 \| 2.56 \| 0.54 \| 6.73 \| 0.00 \| \| H \| 3.10 \| 0.00 \| 3.50 \| 1.75 \| 3.23 \| 2.15 \| 9.15 \| 3.10 \| 0.00 \| \| I \| 8.48 \| ***39.97*** \| 6.86 \| 2.15 \| 16.02 \| 12.38 \| 2.29 \| 0.27 \| 8.75 \| \| K \| 9.83 \| 0.40 \| 11.17 \| 11.31 \| 6.46 \| 5.79 \| 2.42 \| 13.59 \| 0.81 \| \| L \| 6.46 \| ***12.65*** \| 1.88 \| 1.35 \| 13.59 \| 10.23 \| 2.56 \| 7.00 \| ***56.66*** \| \| M \| 3.23 \| 0.54 \| 2.42 \| 0.40 \| 2.29 \| 2.69 \| 0.94 \| 1.08 \| 4.17 \| \| N \| 1.08 \| 0.40 \| 19.38 \| 3.77 \| 3.77 \| 3.50 \| 5.52 \| 7.27 \| 0.00 \| \| P \| 0.13 \| 3.23 \| 0.27 \| 7.67 \| 1.08 \| 1.88 \| 1.48 \| 0.27 \| 0.13 \| \| Q \| 4.71 \| 0.54 \| 6.59 \| 5.65 \| 2.83 \| 4.85 \| 8.21 \| 11.04 \| 0.13 \| \| R \| 6.73 \| 0.27 \| 0.81 \| 4.17 \| 2.15 \| 2.56 \| 1.48 \| 11.04 \| 0.13 \| \| S \| 6.06 \| 0.94 \| 7.94 \| 7.27 \| 2.69 \| 3.90 \| 6.73 \| 5.52 \| 0.00 \| \| T \| 4.44 \| 1.21 \| 2.29 \| 4.31 \| 3.23 \| 4.44 \| 7.67 \| 3.77 \| 0.13 \| \| V \| 11.84 \| ***36.20*** \| 1.48 \| 3.36 \| 6.46 \| 6.59 \| 4.85 \| 1.48 \| ***18.57*** \| \| W \| 3.23 \| 0.00 \| 0.13 \| 0.13 \| 2.29 \| 4.44 \| 0.27 \| 0.00 \| 0.00 \| \| Y \| 11.84 \| 0.27 \| 4.04 \| 0.94 \| 3.23 \| 0.94 \| 1.62 \| 4.98 \| 3.77 \|   **A** | \| Position \| \| \| \| \| \| \| \| \| \| \| --- \| --- \| --- \| --- \| --- \| --- \| --- \| --- \| --- \| --- \| \| Residue \| 1 \| 2 \| 3 \| 4 \| 5 \| 6 \| 7 \| 8 \| 9 \| \| A \| 4.55 \| 2.19 \| 22.39 \| 5.56 \| 3.87 \| 3.37 \| 6.06 \| 5.56 \| 1.18 \| \| C \| 0.00 \| 0.17 \| 0.00 \| 0.00 \| 0.00 \| 0.00 \| 0.00 \| 0.17 \| 0.00 \| \| D \| 0.34 \| 0.17 \| 0.17 \| 16.50 \| 4.38 \| 0.84 \| 8.59 \| 0.51 \| 0.00 \| \| E \| 1.01 \| 0.34 \| 1.35 \| 15.99 \| 4.71 \| 4.21 \| 27.10 \| 11.28 \| 0.51 \| \| F \| 9.76 \| 0.51 \| 6.06 \| 1.52 \| 11.11 \| 24.07 \| 1.01 \| 4.38 \| 5.39 \| \| G \| 1.85 \| 0.67 \| 2.02 \| 7.07 \| 6.23 \| 2.69 \| 1.85 \| 6.73 \| 0.34 \| \| H \| 2.36 \| 0.00 \| 2.19 \| 1.35 \| 3.03 \| 2.36 \| 6.90 \| 3.37 \| 0.17 \| \| I \| 11.11 \| ***41.58*** \| 7.07 \| 2.19 \| 16.33 \| 13.80 \| 2.86 \| 0.67 \| 6.40 \| \| K \| 9.93 \| 0.17 \| 10.27 \| 10.27 \| 5.72 \| 5.05 \| 2.19 \| 13.30 \| 0.51 \| \| L \| 7.58 \| ***11.11*** \| 1.68 \| 2.02 \| 13.64 \| 9.93 \| 3.20 \| 7.58 \| ***56.73*** \| \| M \| 2.69 \| 1.01 \| 2.02 \| 0.00 \| 2.19 \| 1.52 \| 0.51 \| 1.18 \| 4.21 \| \| N \| 1.01 \| 0.00 \| 21.21 \| 4.04 \| 4.38 \| 4.04 \| 4.38 \| 7.74 \| 0.67 \| \| P \| 0.34 \| 3.70 \| 0.51 \| 9.43 \| 2.19 \| 2.36 \| 1.68 \| 0.51 \| 0.17 \| \| Q \| 3.70 \| 1.35 \| 6.90 \| 6.57 \| 3.54 \| 4.04 \| 8.25 \| 10.27 \| 0.34 \| \| R \| 5.39 \| 0.34 \| 0.34 \| 3.54 \| 2.02 \| 2.86 \| 2.02 \| 9.76 \| 0.34 \| \| S \| 5.89 \| 1.52 \| 7.41 \| 6.90 \| 2.19 \| 4.38 \| 7.41 \| 6.40 \| 0.17 \| \| T \| 5.39 \| 2.02 \| 4.04 \| 3.87 \| 3.54 \| 5.22 \| 8.42 \| 4.21 \| 0.17 \| \| V \| 13.64 \| ***33.16*** \| 1.52 \| 2.86 \| 7.41 \| 6.73 \| 5.89 \| 2.36 \| ***19.02*** \| \| W \| 1.68 \| 0.00 \| 0.00 \| 0.17 \| 0.67 \| 1.52 \| 0.17 \| 0.00 \| 0.00 \| \| Y \| 11.78 \| 0.00 \| 2.86 \| 0.17 \| 2.86 \| 1.01 \| 1.52 \| 4.04 \| 3.70 \|   **B** |
| --- | --- | --- | --- | --- | --- | --- | --- | --- | --- | --- | --- | --- | --- | --- | --- | --- | --- | --- | --- | --- | --- | --- | --- | --- | --- | --- | --- | --- | --- | --- | --- | --- | --- | --- | --- | --- | --- | --- | --- | --- | --- | --- | --- | --- | --- | --- | --- | --- | --- | --- | --- | --- | --- | --- | --- | --- | --- | --- | --- | --- | --- | --- | --- | --- | --- | --- | --- | --- | --- | --- | --- | --- | --- | --- | --- | --- | --- | --- | --- | --- | --- | --- | --- | --- | --- | --- | --- | --- | --- | --- | --- | --- | --- | --- | --- | --- | --- | --- | --- | --- | --- | --- | --- | --- | --- | --- | --- | --- | --- | --- | --- | --- | --- | --- | --- | --- | --- | --- | --- | --- | --- | --- | --- | --- | --- | --- | --- | --- | --- | --- | --- | --- | --- | --- | --- | --- | --- | --- | --- | --- | --- | --- | --- | --- | --- | --- | --- | --- | --- | --- | --- | --- | --- | --- | --- | --- | --- | --- | --- | --- | --- | --- | --- | --- | --- | --- | --- | --- | --- | --- | --- | --- | --- | --- | --- | --- | --- | --- | --- | --- | --- | --- | --- | --- | --- | --- | --- | --- | --- | --- | --- | --- | --- | --- | --- | --- | --- | --- | --- | --- | --- | --- | --- | --- | --- | --- | --- | --- | --- | --- | --- | --- | --- | --- | --- | --- | --- | --- | --- | --- | --- | --- | --- | --- | --- | --- | --- | --- | --- | --- | --- | --- | --- | --- | --- | --- | --- | --- | --- | --- | --- | --- | --- | --- | --- | --- | --- | --- | --- | --- | --- | --- | --- | --- | --- | --- | --- | --- | --- | --- | --- | --- | --- | --- | --- | --- | --- | --- | --- | --- | --- | --- | --- | --- | --- | --- | --- | --- | --- | --- | --- | --- | --- | --- | --- | --- | --- | --- | --- | --- | --- | --- | --- | --- | --- | --- | --- | --- | --- | --- | --- | --- | --- | --- | --- | --- | --- | --- | --- | --- | --- | --- | --- | --- | --- | --- | --- | --- | --- | --- | --- | --- | --- | --- | --- | --- | --- | --- | --- | --- | --- | --- | --- | --- | --- | --- | --- | --- | --- | --- | --- | --- | --- | --- | --- | --- | --- | --- | --- | --- | --- | --- | --- | --- | --- | --- | --- | --- | --- | --- | --- | --- | --- | --- | --- | --- | --- | --- | --- | --- | --- | --- | --- | --- | --- | --- | --- | --- | --- | --- | --- | --- | --- | --- | --- | --- | --- | --- | --- | --- | --- | --- | --- | --- | --- | --- | --- | --- | --- | --- | --- | --- | --- | --- | --- | --- | --- | --- | --- | --- | --- | --- | --- | --- | --- | --- | --- | --- | --- | --- | --- | --- | --- | --- | --- | --- | --- | --- | --- | --- | --- | --- | --- | --- | --- | --- | --- | --- | --- | --- | --- |
| \| Position \| \| \| \| \| \| \| \| \| \| \| --- \| --- \| --- \| --- \| --- \| --- \| --- \| --- \| --- \| --- \| \| Residue \| 1 \| 2 \| 3 \| 4 \| 5 \| 6 \| 7 \| 8 \| 9 \| \| A \| 4,65 \| 2,49 \| 19,93 \| 5,32 \| 4,49 \| 3,16 \| 5,65 \| 5,98 \| 1,33 \| \| C \| 0,17 \| 0,17 \| 0,00 \| 0,00 \| 0,00 \| 0,00 \| 0,00 \| 0,00 \| 0,00 \| \| D \| 1,16 \| 0,33 \| 0,33 \| 14,62 \| 4,32 \| 0,83 \| 9,30 \| 0,83 \| 0,17 \| \| E \| 0,50 \| 0,33 \| 1,66 \| 17,77 \| 3,49 \| 3,32 \| 28,41 \| 10,30 \| 0,50 \| \| F \| 10,80 \| 0,83 \| 6,81 \| 1,99 \| 12,62 \| 25,42 \| 1,33 \| 5,98 \| 5,32 \| \| G \| 2,33 \| 0,83 \| 1,83 \| 7,81 \| 5,81 \| 1,83 \| 2,66 \| 5,48 \| 0,33 \| \| H \| 2,16 \| 0,17 \| 2,82 \| 1,00 \| 2,66 \| 2,33 \| 7,81 \| 2,82 \| 0,17 \| \| I \| 9,47 \| ***40,53*** \| 8,31 \| 2,66 \| 16,45 \| 12,29 \| 3,49 \| 0,83 \| 7,64 \| \| K \| 8,97 \| 0,83 \| 10,13 \| 9,80 \| 5,81 \| 5,15 \| 2,16 \| 12,46 \| 0,33 \| \| L \| 6,64 \| ***12,13*** \| 2,49 \| 2,49 \| 14,29 \| 11,13 \| 3,49 \| 8,31 \| ***56,98*** \| \| M \| 2,16 \| 0,66 \| 2,66 \| 0,00 \| 2,16 \| 2,16 \| 0,66 \| 1,33 \| 3,32 \| \| N \| 0,66 \| 0,00 \| 18,27 \| 3,82 \| 3,82 \| 3,32 \| 4,65 \| 6,64 \| 0,83 \| \| P \| 0,50 \| 2,49 \| 0,83 \| 8,80 \| 2,33 \| 3,32 \| 2,33 \| 0,50 \| 0,33 \| \| Q \| 2,99 \| 1,16 \| 6,48 \| 5,15 \| 2,99 \| 3,65 \| 7,31 \| 10,13 \| 0,50 \| \| R \| 5,15 \| 0,50 \| 1,66 \| 3,99 \| 2,66 \| 2,33 \| 1,50 \| 10,30 \| 0,33 \| \| S \| 6,64 \| 1,50 \| 6,31 \| 7,31 \| 1,16 \| 3,32 \| 4,65 \| 6,48 \| 0,33 \| \| T \| 4,49 \| 2,33 \| 2,82 \| 3,99 \| 2,82 \| 4,65 \| 7,81 \| 4,65 \| 0,50 \| \| V \| 14,62 \| 32,56 \| 2,82 \| 2,82 \| 6,81 \| 7,97 \| 5,15 \| 2,66 \| ***16,78*** \| \| W \| 1,99 \| 0,17 \| 0,00 \| 0,17 \| 1,83 \| 2,66 \| 0,17 \| 0,00 \| 0,00 \| \| Y \| 13,95 \| 0,00 \| 3,82 \| 0,50 \| 3,49 \| 1,16 \| 1,50 \| 4,32 \| 4,32 \|   **C** | \| Position \| \| \| \| \| \| \| \| \| \| \| --- \| --- \| --- \| --- \| --- \| --- \| --- \| --- \| --- \| --- \| \| Residue \| 1 \| 2 \| 3 \| 4 \| 5 \| 6 \| 7 \| 8 \| 9 \| \| A \| 9.05 \| 4.02 \| 19.92 \| 4.83 \| 4.43 \| 3.02 \| 5.03 \| 4.43 \| 2.21 \| \| C \| 0.20 \| 0.00 \| 0.00 \| 0.00 \| 0.00 \| 0.00 \| 0.00 \| 0.00 \| 0.00 \| \| D \| 0.20 \| 0.80 \| 0.80 \| 18.71 \| 4.23 \| 1.01 \| 9.66 \| 1.61 \| 0.00 \| \| E \| 1.61 \| 0.80 \| 1.21 \| 17.51 \| 5.23 \| 2.01 \| 17.91 \| 8.85 \| 0.20 \| \| F \| 6.64 \| 1.61 \| 10.06 \| 1.61 \| 12.27 \| 23.94 \| 1.81 \| 4.63 \| 6.04 \| \| G \| 5.23 \| 0.20 \| 2.62 \| 7.44 \| 10.66 \| 3.22 \| 1.01 \| 9.46 \| 0.00 \| \| H \| 1.21 \| 0.00 \| 2.01 \| 1.01 \| 1.81 \| 1.41 \| 8.05 \| 3.42 \| 0.20 \| \| I \| 5.43 \| ***44.47*** \| 12.07 \| 2.01 \| 15.29 \| 17.51 \| 4.63 \| 1.21 \| 6.84 \| \| K \| 17.51 \| 0.40 \| 7.65 \| 7.44 \| 6.04 \| 3.82 \| 3.02 \| 11.07 \| 0.00 \| \| L \| 6.24 \| ***16.90*** \| 3.22 \| 2.21 \| 10.66 \| 16.70 \| 6.44 \| 9.26 \| ***62.98*** \| \| M \| 2.82 \| 0.60 \| 1.61 \| 0.40 \| 2.01 \| 1.21 \| 1.21 \| 1.01 \| 2.01 \| \| N \| 1.41 \| 0.00 \| 15.09 \| 3.82 \| 3.62 \| 2.21 \| 4.43 \| 8.45 \| 0.40 \| \| P \| 0.40 \| 2.21 \| 1.01 \| 9.46 \| 2.82 \| 2.21 \| 1.61 \| 1.01 \| 0.20 \| \| Q \| 3.62 \| 0.60 \| 5.84 \| 5.03 \| 2.62 \| 3.02 \| 8.05 \| 10.06 \| 0.20 \| \| R \| 9.86 \| 0.40 \| 1.01 \| 3.22 \| 2.21 \| 1.21 \| 3.42 \| 11.67 \| 0.20 \| \| S \| 8.05 \| 0.60 \| 6.44 \| 9.05 \| 3.02 \| 2.41 \| 7.24 \| 5.63 \| 0.00 \| \| T \| 4.83 \| 0.80 \| 2.41 \| 3.22 \| 3.22 \| 2.82 \| 8.65 \| 4.02 \| 0.00 \| \| V \| 9.05 \| ***25.55*** \| 3.22 \| 2.41 \| 4.02 \| 5.84 \| 5.84 \| 1.61 \| ***15.49*** \| \| W \| 1.21 \| 0.00 \| 0.20 \| 0.40 \| 3.02 \| 5.43 \| 0.40 \| 0.00 \| 0.00 \| \| Y \| 5.43 \| 0.00 \| 3.62 \| 0.20 \| 2.82 \| 1.01 \| 1.61 \| 2.62 \| 3.02 \|   **D** |
